# Acceptance of Different Self-sampling Methods for Semiweekly SARS-CoV-2 Testing in Asymptomatic Children and Childcare Workers at German Day Care Centers: A Nonrandomized Controlled Trial

**DOI:** 10.1001/jamanetworkopen.2022.31798

**Published:** 2022-09-15

**Authors:** Geraldine Engels, Johannes Forster, Andrea Streng, Viktoria Rücker, Paul Rudolph, Franziska Pietsch, Julia Wallstabe, Lars Wallstabe, Maike Krauthausen, Julia Schmidt, Timo Ludwig, Carsten Bauer, David Gierszewski, Jesper Bendig, Sandra Timme, Thomas Jans, Benedikt Weißbrich, Marcel Romanos, Lars Dölken, Peter Heuschmann, Christoph Härtel, Ildikó Gágyor, Marc Thilo Figge, Johannes Liese, Oliver Kurzai

**Affiliations:** 1 Department of Pediatrics, University Hospital Wuerzburg, Würzburg, Germany; 2 Institute for Hygiene and Microbiology, University of Wuerzburg, Würzburg, Germany; 3 Institute of Clinical Epidemiology and Biometry, University of Wuerzburg, Würzburg, Germany; 4 Leibniz Institute for Natural Product Research and Infection Biology–Hans-Knoell-Institute, Jena, Germany; 5 Department of General Practice, University Hospital Wuerzburg, Würzburg, Germany; 6 Department of Child and Adolescent Psychiatry, Psychosomatics and Psychotherapy, University Hospital Wuerzburg, Würzburg, Germany; 7 Institute for Virology and Immunobiology, University of Wuerzburg, Würzburg, Germany; 8 Clinical Trial Center Wuerzburg, University Hospital Wuerzburg, Würzburg, Germany

## Abstract

**Importance:**

Closure of day care centers (DCCs) to contain the COVID-19 pandemic has been associated with negative effects on children’s health and well-being.

**Objective:**

To investigate the acceptance of self-sampling methods for continuous SARS-CoV-2 surveillance among asymptomatic children and childcare workers (CCWs) in DCCs.

**Design, Setting, and Participants:**

This nonrandomized pilot study included children and CCWs at 9 DCCs in Wuerzburg, Germany, from May to July 2021.

**Interventions:**

Twice weekly testing for SARS-CoV-2 was conducted by self-sampled mouth-rinsing fluid (saliva sampling [SAL], with subsequent pooled polymerase chain reaction test) plus nasal rapid antigen self-test (RAgT) (group 1), SAL only (group 2), or RAgT only (group 3) in children and CCWs.

**Main Outcomes and Measures:**

Main outcomes were rates for initial acceptance and successful (≥60% of scheduled samples) long-term participation. The probability of SARS-CoV-2 introduction into DCCs was modeled as a function of age-adjusted background incidence and DCC size.

**Results:**

Of 836 eligible children, 452 (54.1%; 95% CI, 50.7%-57.4%) participated (median [IQR] age: 4 [3-5] years; 213 [47.1%] girls), including 215 (47.6%) in group 1, 172 (38.1%) in group 2, and 65 (14.4%) in group 3. Of 190 CCWs, 139 (73.2%; 95% CI, 66.4%-79.0%) participated (median [IQR] age: 30 [25-46] years; 128 [92.1%] women), including 96 (69.1%) in group 1, 29 (20.9%) in group 2, and 14 (10.1%) in group 3. Overall, SARS-CoV-2 PCR tests on 5306 SAL samples and 2896 RAgTs were performed in children, with 1 asymptomatic child detected by PCR from SAL. Successful long-term participation was highest in group 2 (SAL only; children: 111 of 172 [64.5%]; CCWs: 18 of 29 [62.1%]). Weekly participation rates in children ranged from 54.0% to 83.8% for SAL and from 44.6% to 61.4% for RAgT. Participation rates decreased during the study course (*P* < .001). The probability of SARS-CoV-2 introduction into a DCC with 50 children was estimated to reach at most 5% for an age-adjusted SARS-CoV-2 incidence below 143.

**Conclusions and Relevance:**

Self-sampling for continuous SARS-CoV-2 testing was well accepted, with SAL being the preferred method. Given the high number of negative tests, thresholds for initiating continuous testing should be established based on age-adjusted SARS-CoV-2 incidence rates.

**Trial Registration:**

German Registry for Clinical Trials Identifier: DRKS00025546

## Key Points

**Question:**

What is the acceptance and feasibility of different methods of twice weekly SARS-CoV-2 monitoring in asymptomatic children and childcare workers in day care centers?

**Findings:**

In this nonrandomized controlled trial and feasibility study with 452 children and 139 childcare workers, self-sampled surveillance testing via saliva sampling and/or nasal rapid antigen self-test for SARS-CoV-2 was well accepted and provided a high sense of safety.

**Meaning:**

These findings suggest that self-sampled continuous testing allowing continued day care for children should be established based on age-adjusted SARS-CoV-2 incidence rates.

## Teaser

This nonrandomized controlled trial investigates the acceptance of self-sampling methods for continuous SARS-CoV-2 surveillance among asymptomatic children and childcare workers at day care centers in Germany.

## Introduction

COVID-19 in young children is usually a mild upper respiratory disease with low risk of complications. The emergence and increased incidence of infections with newer SARS-CoV-2 variants has not led to substantial revisions of this finding.^1,2^

Far-reaching infection control measures were initially introduced, including closure of day care centers (DCCs) and schools,^3^ despite growing evidence in the first 2 years of the COVID-19 pandemic pointing against a relevant role of young children as important sources of infection.^4,5,6^ Closure of childcare facilities may result in developmental delay in young children^7,8^ and has been identified as a relevant stressor for families during the COVID-19 pandemic.^9^ Loss of access to nonparental childcare has been shown to be associated with increased job loss risk for mothers.^10^ Parental exhaustion, social isolation, and job loss are potential risk factors for child maltreatment.^10,11,12^

Continuous SARS-CoV-2 monitoring of asymptomatic children and childcare workers (CCWs) is a key measure for avoiding DCC closure by early detection of SARS-CoV-2 clusters. Different sampling methods have been investigated (eg, anal or buccal swab, saliva, midturbinate swab), and pooling has been proposed to save resources.^13,14,15,16^

PCR-based analyses have so far been favored due to their ability to detect low viral loads in asymptomatic individuals.^17,18^ However, limited availability, high costs, and the need for well-equipped PCR laboratories are drawbacks. For the early detection of infected asymptomatic individuals, a short time-to-result interval might be more relevant than high test sensitivity,^19^ and rapid antigen self-tests (RAgTs) have been shown to fulfil the requirements for easy-to-use, rapid, point-of-care tests with a lower but still acceptable limit of detection.^20^

In a previous study in DCCs,^16^ we found that acceptance of noninvasive saliva sampling was superior to nasal swabbing by medically trained personnel. In the present study, we focused on noninvasive sampling combined with resource-saving pooled PCR testing and compared its feasibility with self-sampled RAgTs. We evaluated these continuous surveillance approaches for SARS-CoV-2 in asymptomatic children and CCWs in DCCs and analyzed attitudes and psychosocial factors viewed as potentially influential factors. In addition, we developed a model to estimate the probability of SARS-CoV-2 introduction into the DCC to support decision-making on a reasonable start and end point for continuous surveillance.

## Methods

### Design and Conduct

The study was performed as a multicenter, longitudinal study from May 2021 to July 2021 in 9 preselected DCCs with 959 children and 190 CCWs (range: 36-165 children and 12-28 CCWs per DCC) in the city of Wuerzburg, Bavaria (approximately 130 000 inhabitants). All 9 DCCs, covering approximately 20% of all DCC-attending children in the area, had participated in a previous surveillance study.^16^

The study protocol was approved by the ethics committee of the University Hospital Wuerzburg. Written informed consent was obtained from all CCWs and parents or guardians of children. The study follows the Transparent Reporting of Evaluations with Nonrandomized Designs (TREND) reporting guideline for nonrandomized controlled trials. The trial protocol appears in Supplement 1.

**Figure 1.**
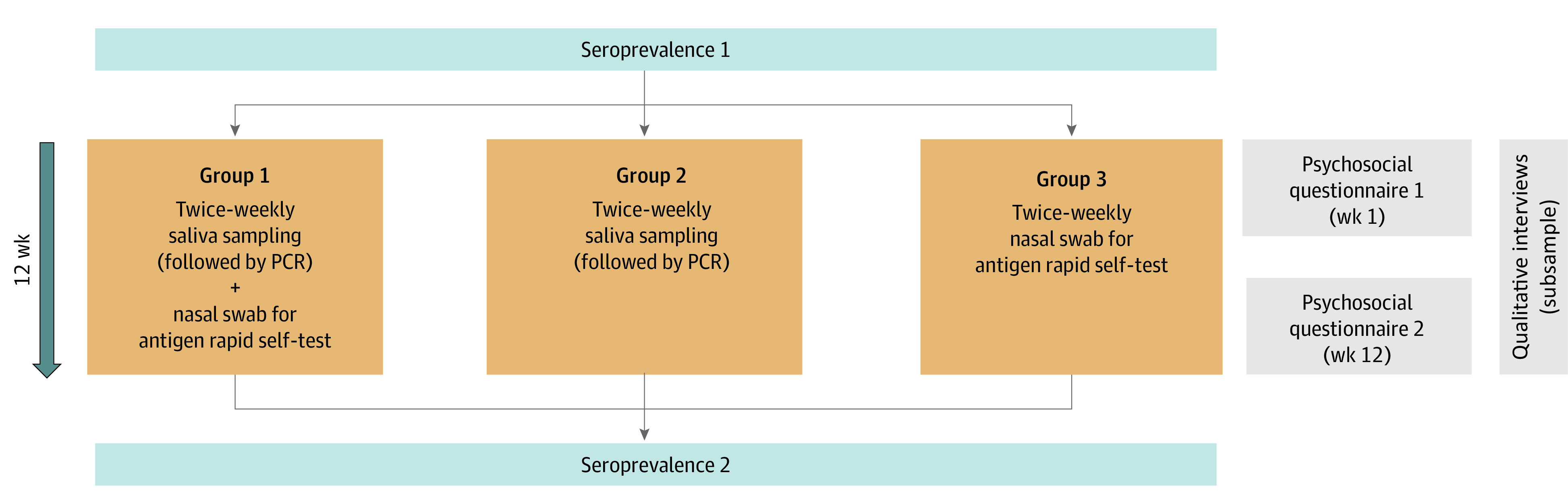
Overview of the Study Design After informed consent by parents and childcare workers, asymptomatic day care center–attending children and childcare workers participated in twice weekly self-sampling of respiratory secretions scheduled for a 12-week observation period (May to July 2021). Participants could initially choose between 3 study groups, including self-sampling of mouth-rinsing fluid (saliva sampling), followed by pooled polymerase chain reaction (PCR) testing and/or rapid antigen self-testing. (For children, this was performed by their parents.) Before and after the period of continuous testing, SARS-CoV-2 seroprevalence status of children and childcare workers was determined by finger-prick testing, and parents and childcare workers answered psychosocial questionnaires. In addition, qualitative interviews on participants’ attitudes in a subsample of parents and childcare workers were conducted.

The study design is summarized in Figure 1. After informed consent, parents of children aged 2 to 6 years and CCWs could opt for 1 of 3 approaches (ie, groups) for continuous twice weekly SARS-CoV-2 testing by home-based self-sampling for a period of 12 weeks. Group 1 performed self-sampling of mouth-rinsing fluid (saliva sampling [SAL]) for subsequent pooled PCR testing plus a nasal swab-based RAgT, both conducted on the same day; group 2, only SAL; and group 3, only RAgT. For each self-sampling method, written and online video instructions were provided. Before and after the study period, seroprevalence status was determined at the DCCs by point-of-care finger-prick testing. Details of methods and materials are provided in eAppendix 1 in Supplement 2, and the flow diagram appears in eFigure 1 in Supplement 2.

### Questionnaires and Interviews

In week 1 and week 12, CCWs and parents answered online questionnaires on sociodemographic data, health status, general attitudes toward the pandemic, and their perception of the feasibility and burden of continuous testing. More information appears in eAppendix 1 in Supplement 2.

### End Points

The primary end point was defined as the overall initial acceptance, defined as written informed consent to participate, among all eligible children and CCWs. Secondary end points included the successful long-term participation of children and CCWs (predefined as individuals with ≥60% of all scheduled samples being collected), detection of SARS-CoV-2 infections, SARS-CoV-2 serostatus, and children’s/parents’ and CCWs’ attitudes at baseline and at the end of the study. Potential factors associated with successful long-term participation were also analyzed.

### Statistical Analysis

All end points were analyzed descriptively (frequency [percentage], or median [IQR]), stratified by children and parents or CCWs. The 95% CIs for the primary end points were estimated using the Score Wilson method.^21^ As a sensitivity analysis, we adjusted for clustering and estimated the participation rates with generalized linear mixed models with logit link function and random intercept for DCC. Psychosocial data were evaluated overall and by study group and compared using the χ^2^ test or Fisher exact test (if assumptions for χ^2^ test were not met). Comparisons over time used the Cochrane Armitage trend test for weekly participation rates and McNemar test or Bowker test for categorical data. Factors associated with unsuccessful participation were determined using univariable and multivariable logistic regression analysis (eAppendix 1 in Supplement 2). The significance level was set at 5% and was not adjusted for multiple testing, as analyses were considered as exploratory. All tests were 2-tailed.

For the mathematical model of the probability of SARS-CoV-2 introduction into DCCs, age-adjusted local weekly incidences for the total DCC-relevant age group of 2 to 6 years and the corresponding incidences among children attending any of the 69 DCCs in the city of Wuerzburg (including the 9 DCCs participating in the main study) were obtained. Assuming that the introduction of a primary SARS-CoV-2 infection depends on the age-adjusted background incidence and DCC size (number of attending children), the probability of at least 1 DCC child contracting infection in 1 week was estimated using a disease occurrence model^22^ (eAppendix 1 in Supplement 2).

## Results

### Initial Study Consent and Distribution of Participants Across Study Groups

At the 9 participating DCCs, there were 836 children (range, 31-130) and 190 CCWs (range, 12-28). Parents of 452 of 836 eligible children aged 2 years or older (54.1%; 95% CI, 50.7%-57.4%) consented to regular self-sampling of their children, with 215 (47.6%) opting for group 1 (SAL plus RAgT), 172 (38.1%) for group 2 (SAL only), and 65 (14.4%) for group 3 (RAgT only) (Table 1). Of 190 CCWs, 139 (73.2%; 95%CI, 66.4%-79.0%) consented to participate; with 96 (69.1%) opting for group 1, 29 (20.9%) for group 2, and 14 (10.1%) for group 3. Across the 9 DCCs, the participation rate of children ranged from 44.3% to 80.0% in children and from 31.3% to 100% in CCWs. Participation rate adjusted for clustering were 54.1% (95% CI, 44.3%-63.5%) among children and 76.5% (95% CI, 59.7%-87.8%) among CCWs.

**Table 1.** Distribution of Study Participants in the Respiratory Surveillance Across the 3 Study Groups With Sociodemographic Data

Characteristic	Participants, No./total No. (%)			
	Total	Group 1 (SAL and RAgT)	Group 2 (SAL)	Group 3 (RAgT)
Children	452	215 (47.6)	172 (38.1)	65 (14.4)
Age, median (IQR), y	4 (3-5)	4 (3-5)	4 (3-5)	3 (2-4)
Girls	213/452 (47.1)	94/215 (43.7)	88/172 (51.2)	31/65 (47.7)
Boys	239/452 (52.9)	121/215 (56.3)	84/172 (48.8)	34/65 (52.3)
Chronic health conditions (any)	51/373 (13.7)	29/193 (15.0)	14/125 (11.2)	8/55 (14.5)
CCWs	139	96 (69.1)	29 (20.9)	14 (10.1)
Age, median (IQR), y	30 (25-46)	30 (25-45)	33 (24-52)	30 (25-37)
Women	102/109 (93.6)	74/78 (94.9)	18/19 (94.7)	10/12 (83.3)
Men	7/109 (6.4)	4/78 (5.1)	1/19 (5.3)	2/12 (16.7)
Chronic health conditions (any)	24/110 (21.8)	17/79 (21.5)	4/20 (20)	3/11 /27.3)
Total	591	311 (52.6)	201 (34.1)	79 (13.4)

Abbreviations: CCW, childcare worker; SAL, saliva testing; RAgT, rapid antigen self-test.

### Sociodemographic Characteristics of Participants and Attitudes and Concerns Regarding the Pandemic

#### Children and Parents

Of the 452 participating children, 213 (47.1%) were female; the median (IQR) age was 4 (3-5) years. The 325 parents responding to the initial questionnaire in May 2021 had a median (IQR) age of 38 (34-40) years; 237 of 323 (73.4%) were female. Of these parents, 281 of 325 (86.5%) considered access to the DCC as fairly or very important for their daily life; 116 of 323 (35.9%) and 247 of 324 (76.2%) considered SARS-CoV-2 as fairly and very dangerous, respectively, to their family or to society, and 186 of 324 (57.4%) felt fairly or strongly limited in their personal lives. Regarding the planned surveillance measures, 222 of 323 (68.7%) expected a fairly or very strong positive impact on their sense of safety. At the time of the initial questionnaire, 120 of 325 parents (36.9%) reported that they had been vaccinated against SARS-CoV-2, and 168 (51.7%) stated their willingness to be vaccinated; 7 (2.2%) reported a previous SARS-CoV-2 infection. For full details, see Table 1 and eAppendix 2 and eTable 1 in Supplement 2.

By week 12 in July 2021, 161 of 224 parents (71.9%) had been vaccinated against SARS-CoV-2, a significant difference from the start of the study (*P* < .001). The proportion considering SARS-CoV-2 dangerous for themselves and their families decreased to 65 of 223 (29.1%; *P* = .01), and fewer parents felt limited in their personal lives (29 of 223 [17.5%]; *P* < .001) (eTable 3 in Supplement 2).

#### CCWs

Of the 139 participating CCWs, 128 (92.1%) were women, with a median (IQR) age of 30 (25-46) years. Initial questionnaires were available for 112 of 139 CCWs. Of these, 71 (63.9%) felt limited in their personal life due to the pandemic; 59 (53.1%) and 84 (75.6%) considered SARS-CoV-2 as fairly or very dangerous to their family and to society, respectively, and 26 (23.6%) indicated a strong fear of contracting COVID-19. A fairly or very strong positive effect of the planned surveillance measures on their sense of safety was expected by 82 (73.2%). Overall, 37 (27.0%) were vaccinated against SARS-CoV-2; vaccination data were not available for 50 CCWs (eAppendix 2 and eTable 2 in Supplement 2). In week 12, 45 of 64 responding CCWs (70.3%) reported being vaccinated against SARS-CoV-2. Compared with week 1, fewer CCWs considered SARS-CoV-2 a danger for themselves or their families (23 of 62 [37.1%]; *P* = .001) or felt limited in their personal lives (26 [41.9%]; *P* = .01) (eTable 4 in Supplement 2).

### Results of Pooled PCR Testing, RAgTs, and Antibody Testing

#### Children

During the 12-week observation period, 5306 SAL samples from children were analyzed for SARS-CoV-2 by pooled PCR. One child tested positive, but intensified testing in the respective DCC identified no secondary cases. All 2896 RAgTs from nasal atrium swabs were reported as negative by the parents.

At week 12, SARS-CoV-2 antibodies were detected in 6 of 278 children (2.2%). Five of these six children (83.3%) already had antibodies for SARS-CoV-2 at week 1. One asymptomatic child from group 2 who had tested negative by SAL and PCR in 21 of 24 possible tests (3 tests were not performed due to absence) had a positive point-of-care antibody test at week 12.

#### CCWs

All PCRs performed on 1491 saliva samples and all 1022 RAgTs of CCWs were negative. At week 1, SARS-CoV-2 antibodies were detected in 3 of 105 CCWs (2.9%), with no additional seropositive individuals at week 12.

### Long-term Participation in Self-sampling, Views on Test Measures, and Factors Associated With Adherence

#### Children

**Figure 2.**
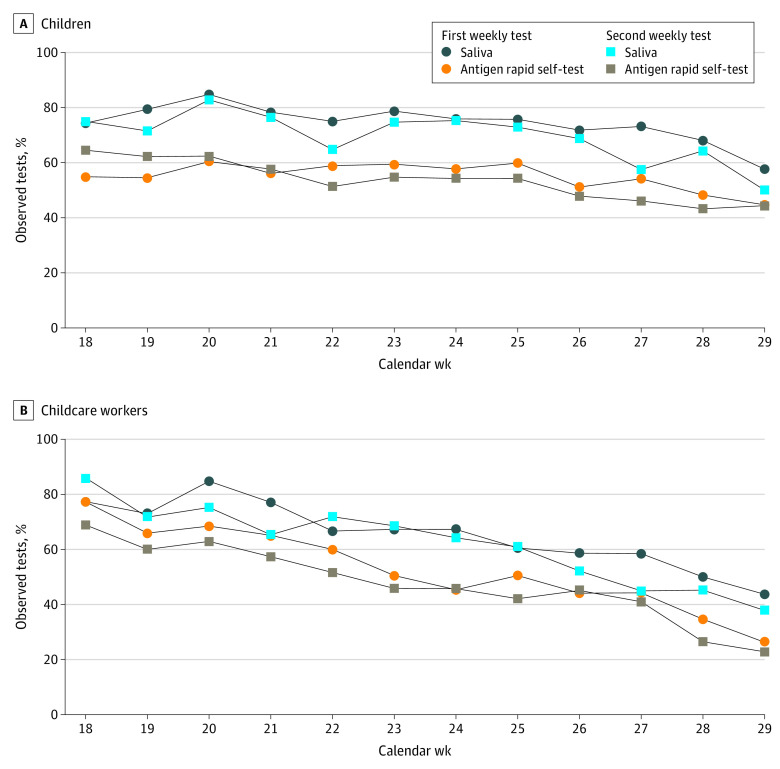
Rates of Respiratory Self-sampling by Saliva Testing or Rapid Antigen Self-testing in Children and Childcare Workers Over Time Data from children and childcare workers using the same sampling method were pooled for this presentation regardless of study group. Data were stratified by the first and second test per week, with the first test performed on Monday or Tuesday and the second test on Thursday or Friday. Participation rates are presented by showing all observed tests as a percentage of all expected tests for the respective test day (ie, excluding holidays, sick days). Study weeks 1 to 4, 5 to 8, and 9 to 12 correspond to calendar weeks 18 to 21, 22 to 25, and 26 to 29. Cochrane-Armitage trend tests for both groups were *P* < .001.

Of the participating children, 271 of 452 (60.0%) successfully completed the surveillance measures, with a range from 40.0% to 69.7% across the 9 DCCs (Table 2). These 271 children corresponded to 32.4% of all 836 eligible children (including nonparticipants) in DCCs. Within the cohort of participating children, the rates for successful participation in groups 1 (SAL plus RAgT), 2 (SAL only), and 3 (RAgT only) were 60.9% (131 of 215), 64.5% (111 of 172), and 44.6% (29 of 65), respectively. Across the 9 DCCs, the successful long-term participation rate ranged among children in group 1 (9 DCCs) from 30.0% to 100.00%, in group 2 (9 DCCs) from 41.4% to 86.7% and in group 3 (8 DCCs) from 0% to 100% (eTable 5 in Supplement 2). The weekly participation rate during the 12-week testing period ranged from 54.0% to 83.8% for SAL and 44.6% to 61.4% for RAgTs (Figure 2A and eTable 5 in Supplement 2). For both methods, participation decreased over time (*P* < .001), particularly between study weeks 9 and 12. For SAL, participation rates were 78.1% in study weeks 1 to 4, 74.7% in weeks 5 to 8, and 64.0% in weeks 9 to 12; for RAgTs, 59.4%, 56.8% and 47.5%, respectively (eTable 5 in Supplement 2).

**Table 2.** Participants With Successful Long-term Participation, Stratified by Study Group for Children and CCWs^a^

Participants	Successful long-term participation, No./total No. (%)			
	Total	Group 1 (SAL and RAgT)	Group 2 (SAL)	Group 3 (RAgT)
Children	271/452 (60.0)	131/215 (60.9)	111/172 (64.5)	29/65 (44.6)
CCWs	71/139 (51.1)	46/96 (47.9)	18 /29 (62.1)	7/14 (50.0)
Total	342/591 (57.9)	177/311 (56.9)	129/201 (64.2)	36/79 (45.6)

Abbreviations: CCW, childcare worker; SAL, saliva testing; RAgT, rapid antigen self-test.

^a^ Long-term participation defined as at least 60% of all scheduled respiratory samples provided by the individual.

Most parents considered screening useful (at week 1: 192 of 221 [86.9%]; week 12: 191 [86.4%]) and the test frequency (ie, twice a week) appropriate (week 1: 200 [89.7%]; week 12: 203 [91.8%]). They were satisfied with the self-sampling process (week 1: 180 [81.1%]; week 12: 173 [77.9%]) and reported no negative impact on the organization of their daily life (week 1: 210 [95.5%]; week 12: 208 [94.5%]). For saliva testing, 100 of 112 parents (89.3%) and 102 (91.1%) reported “very good cooperation” from their children in weeks 1 and 12, respectively, vs 46 of 93 (49.5%) and 43 (46.2%) for RAgTs in weeks 1 and 12, respectively. In week 1, 80 of 125 parents (64.0%) and, in week 12, 92 parents (73.6%) stated that their children “did not like” the RAgT. In multivariable analyses, the probability of unsuccessful long-term participation decreased with increasing child age (odds ratio, 0.60; 95% CI, 0.41-0.88; *P* = .009) (eTable 6 in Supplement 2).

#### CCWs

Overall, 71 of 139 CCWs (51.1%) successfully participated in long-term surveillance, with a range of 20.0% to 61.5% across the 9 DCCs (Table 2). Within groups 1, 2, and 3, the corresponding rates were 47.9% (46 of 96), 62.1% (18 of 29), and 51.1% (7 of 14).

CCWs’ weekly participation rates ranged from 40.9% to 81.7% for SAL and 24.6% to 73.0% for RAgTs (eTable 5 in Supplement 2). Test participation decreased continuously during the study period (Figure 2B) (*P* < .001). For SAL, participation rates were 76.1% in weeks 1 to 4, 65.6% in weeks 5 to 8, and 49% in weeks 9 to 12. Participation rates for RAgT were 65.8%, 49.4% and 35.6%, respectively (eTable 5 in Supplement 2). CCWs considered both SAL (54 of 54 [100%]) and RAgT (week 1: 47 of 50 [94%]; week 12: 50 of 50 [100%]) as uncomplicated; 54 of 62 (87.1%) at week 1 and 53 (85.5%) at week 12 considered the test frequency as appropriate.

Multivariable analyses showed CCWs to be less likely to drop out if they had opted for group 2 (SAL only: OR, 0.23; 95% CI, 0.06-0.93; *P* = .04), were older (OR, 0.95; 95% CI, 0.92-0.99; *P* = .01), or considered SARS-CoV-2 dangerous for themselves and their families (OR, 0.37; 95% CI, 0.15-0.92; *P* = .03) (eTable 7 in Supplement 2).

### Modeling the Probability of SARS-CoV-2 Introduction Into a DCC

We obtained the local SARS-CoV-2 age-adjusted incidence and compared it with the incidence among children attending any of the 69 DCCs in the city of Wuerzburg during the study period. The upper limit of the DCC incidences (eFigure 2 in Supplement 2) served as reference for estimating the probability of a child with primary SARS-CoV-2 infection attending a DCC.

**Figure 3.**
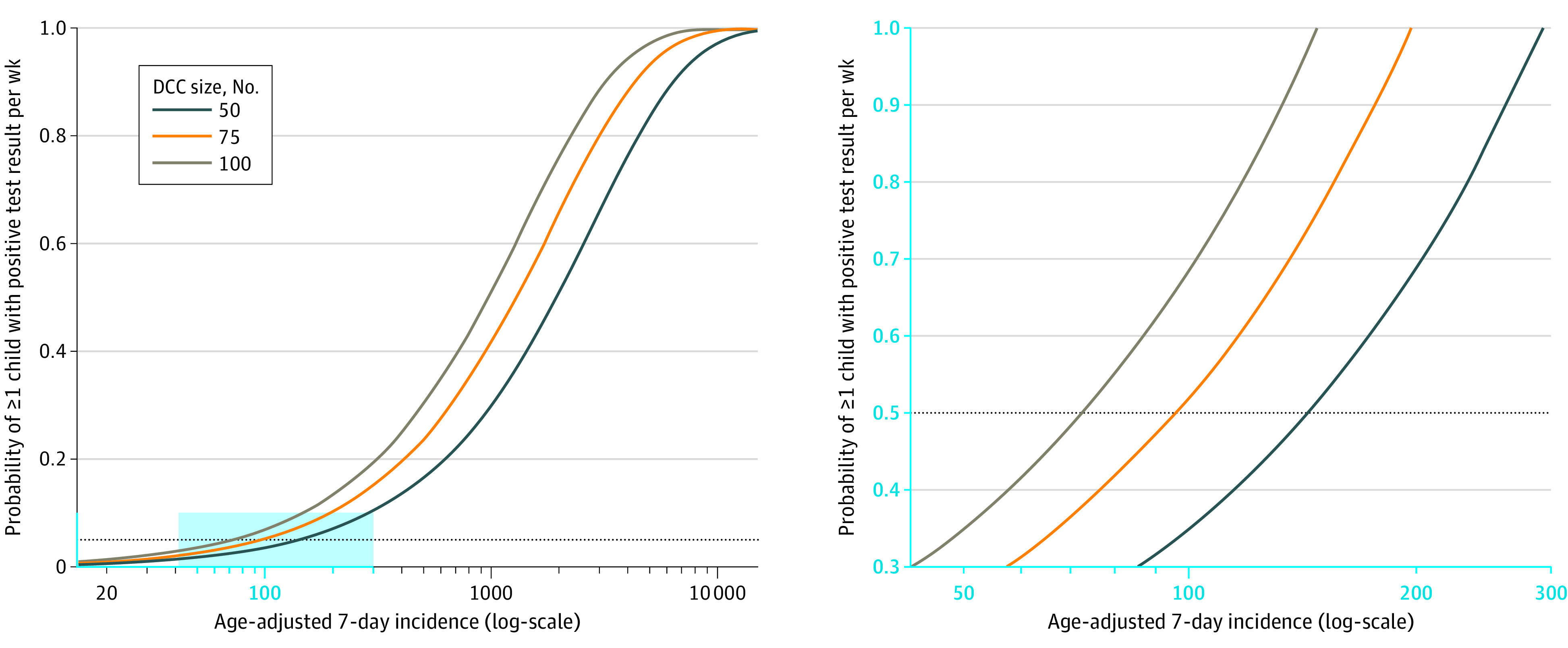
Modeling the Probability of SARS-CoV-2 Introduction Into a Day Care Center (DCC) For the probability of at least 1 child with SARS-CoV-2 (ie, primary case) entering the DCC within 1 week to remain below or at a maximum level of 5%, the age-adjusted incidence must be less than 143, 95, and 71 for DCC sizes of 50, 75, and 100, respectively. The right panel shows the blue rectangle in the left panel in more detail.

At incidences of up to 200 per 100 000, the risk of entry of a child with SARS-CoV-2 was relatively small for all DCC sizes. From incidences of 200 per 100 000 and greater, the risk increases, with a particularly pronounced increase in large DCCs (Figure 3). For DCC sizes of 50, 75, and 100 children, for instance, the age-adjusted SARS-CoV-2 incidence per 100 000 children in the corresponding population needed to exceed 143, 95, and 71, respectively, for the risk of entry of a child with SARS-CoV-2 to reach levels of at least 5%.

## Discussion

In this nonrandomized controlled trial, participation in DCC surveillance by home-based self-sampling of asymptomatic children and CCWs was accepted by more than 50% of parents and 73% of CCWs. Interestingly, despite the higher effort required, most parents and CCWs opted for the combination of SAL and RAgT. The fact that participants favored a combination of both rapid (RAgT) and highly reliable (PCR) test results might reflect a low sense of safety in that phase of the COVID-19 pandemic (May 2021).

Successful long-term self-sampling was achieved by more than 60% of the participating children and by more than 50% of the participating CCWs during the 3-month observation period. For both children and CCWs, long-term acceptance was highest in the group using the less invasive testing method with saliva sampling only. However, successful long-term testing covered only about one-third of the total child population in the 9 DCCs, consisting of participating and nonparticipating children. As previously shown,^16^ this may not be sufficient to detect all SARS-CoV-2 cases introduced into a childcare unit.

In all groups, participation declined during the study. The decline was more pronounced in younger children, possibly due to the greater parental effort needed to obtain samples. Both the decrease in the background incidence of SARS-CoV-2 and an increase in vaccination coverage of CCWs and parents after the extension of COVID-19 vaccinations to all adults in June 2021^23^ may have been associated with participants’ perception of the pandemic threat and contributed to the observed decline in willingness to participate in continuous testing. Nevertheless, in line with our previous study,^16,24^ the positive impact of testing on participants’ sense of safety did not decrease significantly between weeks 1 and 12, suggesting that CCWs and parents may have, whether intentionally or not, adjusted their participation in testing to a level they still considered sufficient for themselves and their children.

Long-term adherence to surveillance was lower for children and CCWs in groups 1 and 3, both of which included RAgTs from nasal atrium swabs, usually perceived as more invasive, less convenient, more time consuming, and less sensitive than saliva self-sampling with external PCR testing. Another approach for continuous surveillance of DCC children is the so-called lolly method (sucking a swab for 30 seconds like a lollipop, followed by sequential pool-testing). In a study by Dewald et al,^14^ this sampling approach was also well accepted, with the drawback that a retention sample is not provided simultaneously.^25^

Surveillance by sample pooling is efficient if only a small number of positive pools is expected and only few samples need to be analyzed individually. In periods with high SARS-CoV-2 incidence, it may therefore not be a reasonable strategy. The implementation of surveillance measures during periods of low SARS-CoV-2 incidence, on the other hand, requires careful evaluation of costs and benefits as the risk of transmission is low.

Our study was conducted during a period of declining background 7-day incidence (from 114 to 6 per 100 000 inhabitants) and detected only 1 asymptomatic child with SARS-CoV-2 infection in approximately 6800 samples and 3900 RAgTs. The seroprevalence results for participants and additional data from the local health department on nonparticipants from our 9 DCCs indicated that our surveillance had not missed any SARS-CoV-2 infections. In 1 additional child with a positive point-of-care seroprevalence test at the end of the study, we suspected either a false-positive test result or an infection during a period of DCC absence.

We used a mathematical model to determine a suitable lower incidence threshold for initiating continuous SARS-CoV-2 surveillance. The setting of such a limit depends on the SARS-CoV-2 incidence, the availability of resources, and political decision-makers’ risk perception. A reasonable cut-off value to begin surveillance measures might be defined close to the level beyond which a steep increase in the risk of introduction of at least 1 infected child in the DCC is to be expected. Our results show that such an increase occurs for age-adjusted incidences between 50 and 200 and that the risk is higher for larger DCC group units.

In contrast, the definition of an upper incidence limit for pooled SARS-CoV-2 testing depends on the availability of laboratory equipment and logistics, in particular fast access to retention samples. A reasonable threshold for switching to individual testing by PCR or RAgT might be close to an incidence of 2000 per 100 000, as positive pool results are then to be expected in DCCs of any size.

As a consequence of the COVID-19 pandemic, lockdowns, including of childcare centers, have been imposed by authorities in many parts of the world, with negative outcomes for children’s psychosocial health and development. Implementation of restrictions and surveillance measures will remain subject to public health and political decision-making. SARS-CoV-2 surveillance via continuous self-sampling analyzed by rapid tests or pooled saliva PCR testing can be applied if laboratory equipment, consumables, and logistics are available and sampling sets are provided for the parents and CCWs and, thus, may help to enable continuous child day care. Modeling provides support for the decision when to start (and end) continuous surveillance in asymptomatic children and CCWs based on DCC size and local age-specific SARS-CoV-2 incidences.

### Limitations

This study has limitations. Besides being restricted to one specific region in Germany, a further limitation of this study was a potential selection bias by preselection of DCCs. A considerable proportion of participants had already participated in a previous SARS-CoV-2 long-term surveillance study in the same DCCs,^16^ which might have had an impact both on initial and long-term participation rates. Participant rates varied considerably across the different DCCs for both children and CCWs, possibly due to local socioeconomic differences between the populations visiting the DCCs. Further limitations include the lack of control of home-based self-sampling and documentation of RAgT results and the exclusion of children younger than 2 years. The study was conducted during a period of declining incidences and increasing SARS-CoV-2 vaccination coverage. Both factors may have contributed to the decline in long-term participation rates. Furthermore, the low incidence levels did not allow a comparison of self-sampling methods regarding test effectiveness.

## Conclusions

In this study, home-based continuous testing for SARS-CoV-2 by PCR from saliva samples and RAgT from nasal atrium was generally well accepted by children and CCWs. Less invasive saliva sampling was the preferred test method with the highest long-term acceptance levels. Acceptance of testing declined during the course of the study, in parallel with decreasing SARS-CoV-2 incidences, increasing vaccination coverage of CCWs and parents, and a decrease in the perceived threat posed by the pandemic. If the objective is to exclude SARS-CoV-2 introduction to DCCs with a probability of at least 95%, continuous testing in DCCs should be started at an age-adjusted incidence rate between 50 and 200 per 100 000.

## Notes

Supplement 1.
Trial Protocol

Supplement 2. **eAppendix 1. **Supplemental Methods **eAppendix 2. **Supplemental Results **eTable 1. **Sociodemographic Characteristics of Participating Children and Parents, Attitudes Toward the Pandemic/Experience of Testing/Perception of Health Burden of the Pandemic, Week 1 **eTable 2. **Sociodemographic Characteristics of Participating Childcare Workers, Attitudes Toward the Pandemic/Experience of Self-testing/Perception of Health Burden of the Pandemic, Week 1 **eTable 3.** Comparison of Children's Health Status/Readiness to Participate and Parents' Perceptions of Respiratory Surveillance Measures/Attitudes Toward the Pandemic at Weeks 1 and 12 **eTable 4. **Comparison of Perceptions of Respiratory Surveillance Measures and Attitudes Toward the Pandemic of Participating Childcare Workers at Weeks 1 and 12 **eTable 5. **Overall Number and Rates of Saliva Testing per Week and per Month Throughout the Study Period for Children and CCWs **eTable 6.** Factors Influencing Children's Unsuccessful Long-term Participation **eTable 7. **Factors Influencing Successful Participation of Childcare Workers **eFigure 1. **Flow Diagram **eFigure 2. **Age-Adjusted Incidences in the City and District of Wuerzburg for the DCC-Relevant Age Group (2-6 years; orange) and Corresponding Incidences Among Children in all 69 DCCs (blue) During the Study Period
eReferences.

Supplement 3.
Nonauthor Collaborators

Supplement 4.
Data Sharing Statement
