# Error in Figure 3

**DOI:** 10.1001/jamanetworkopen.2022.40161

**Published:** 2022-10-12

In the Original Investigation titled “Acceptance of Different Self-sampling Methods for Semiweekly SARS-CoV-2 Testing in Asymptomatic Children and Childcare Workers at German Day Care Centers: A Nonrandomized Controlled Trial,”^1^ published September 15, 2022, there was an error in Figure 3. The y-axis for the graph on the right side should have had a range of 0.03 to 0.10. This article has been corrected.^1^
