# Supplementary material for: Acceptance of Different Self-sampling Methods for Semiweekly SARS-CoV-2 Testing in Asymptomatic Children and Childcare Workers at German Day Care Centers: A Nonrandomized Controlled Trial
Source: JAMA Netw Open. 2022 Sep 15;5(9):e2231798. doi: 10.1001/jamanetworkopen.2022.31798 (PMC9478779; doi:10.1001/jamanetworkopen.2022.31798)
Supplement: Supplement 1. — Trial Protocol [file jamanetwopen-e2231798-s001.pdf]

Wuerzburg child care study during the COVID-19 pandemic, Part II (Wue-KiTa-CoV 2.0):

Surveillance of SARS-CoV-2 infections in daycare centers (KiTas) by means of submission of mouthwash samples 2x/week for pooled PCR and/or antigen rapid testing at home

(pilot project)

Direction of study:

Prof. Dr. med. Johannes Liese, MSc

Prof. Dr. med. Oliver Kurzai

Date of the version:

2021-03-30

The content of this study protocol is confidential and must not be disclosed to persons who are not involved.

Version 1.1

© Copyright Template - IKE-B

TABLE OF CONTENTS

PAGE

1 GENERAL INFORMATION ... 5

1.1 Institutions involved and responsible persons ... 5
1.2 Signatures ... 7
1.3 Abbreviations ... 8
1.4 Synopsis ... 9
1.5 Flow diagram order of study course/recruitment/data acquisition ... 17
1.6 Background: COVID-19 and childcare ... 18
1.7 Questions Wue-Kita-CoV 2.0 ... 23
1.8 Risk-benefit ratio ... 24

2 STUDY OBJECTIVES ... 28

2.1 Primary hypothesis ... 28
2.2 Secondary / further hypotheses ... 28
2.3 Study design and time line ... 29

3 STUDY POPULATION ... 31

3.1 Procedure for the selection of facilities ... 31
3.2 Inclusion criteria for test subjects at the facilities ... 31

4 ORDER OF STUDY ... 32

4.1 Recruitment of study centers and test persons ... 32
4.2 Information and consent ... 32
4.3 Interview and clinical examinations ... 33

5 TEST PERSONS SAFETY, WORK SAFETY ... 40

6 DATA PROCESSING ... 41

6.1 Source data and material ... 41
6.2 Study database ... 42
6.3 Laboratory methods ... 43
6.4 Samples ... 44

7 BIOMETRIC ASPECTS ... 46

7.1 Endpoints ... 46
7.2 Procedure for data analysis ... 48
7.3 Analysis populations ... 49
7.4 Evaluation points ... 49
7.5 Case number discussion and power analysis ... 49

8 QUALITY ASSURANCE AND CONTROL ... 51

8.1 Data flow diagram / responsibilities ... 53

9 PUBLICATION OF THE RESULTS ... 54

10 TEST PERSONS INSURANCE ... 55

11 ETHICAL BASIS ... 56

11.1 Ethics vote ... 56
11.2 Test persons information and consent for sample collection ... 56
11.3 Consent to take part in the study ... 56
11.4 Usage, storage and transfer of the data ... 57

12 LITERATURE 58
13 APPENDIX 60
13.1 Participant information 60
13.2 Declaration of consent 60
13.3 Survey tools 60
13.4 Further information material 60

Preliminary note:

The present study protocol on Wue-KiTa-CoV 2.0 is based on the study protocol of the previous study Wue-KiTa-CoV by the same authors. This previous study protocol has not yet been published and is therefore not yet accessible to the general public.

Since the study was conducted identically to a great extent, these parts of the text were taken over from the previous study protocol unchanged or with only slight modifications (in particular Sections 1.6, 1.8, 3.1, 4.3.2, 4.3.3., 6.2, 8, 9, 10, 11, 12, 13.)

1 GENERAL INFORMATION

1.1 Participating institutions and persons responsible

Study lead:	Prof. Dr. med. Johannes Liese, MSc<br>Department of Pediatrics, University Hospital Wuerzburg<br>Josef-Schneider-Str. 2-D31, 97080 Wuerzburg
	Prof. Dr. med. Oliver Kurzai<br>Institute for Hygiene and Microbiology, University of Wuerzburg<br>Josef-Schneider-Str. 2-E1, 97080 Wuerzburg
Research scientists:	Prof. Dr. med. Lars Dölken<br>Institute of Virology, University of Wuerzburg<br>Versbacher Str. 7, 97078 Wuerzburg
	Dr. med Géraldine Engels<br>Department of Pediatrics, University Hospital Wuerzburg<br>Josef-Schneider-Str. 2-D31, 97080 Wuerzburg
	Dr. med. Johannes Forster<br>Institute for Hygiene and Microbiology, University of Wuerzburg<br>Josef-Schneider-Str. 2-E1, 97080 Wuerzburg
	Prof. Dr. med. Ildikó Gágyor<br>Institute of General Medicine, University Hospital Wuerzburg<br>Josef-Schneider-Str. 2-D7, 97080 Wuerzburg
	Prof. Dr. med. Marcel Romanos<br>Department of Child and Adolescent Psychiatry, University Hospital Wuerzburg<br>Margarete-Höppel-Platz 1, 97080 Wuerzburg
	PD Dr. rer. nat. Andrea Streng<br>Department of Pediatrics, University Hospital Wuerzburg<br>Josef-Schneider-Str. 2-D31, 97080 Wuerzburg
	Dr. med. Benedikt Weißbrich<br>Institute of Virology, University of Wuerzburg<br>Versbacher Str. 7, 97078 Wuerzburg
Consultation:	Dr. Hülya Düber<br>Head of the Office for Youth, Family and Social Services, Wuerzburg City Council, City of Wuerzburg<br>Karmelitenstraße 43, 97070 Wuerzburg
	Monika Kraft<br>Head of the Office for Youth, Family and Social Services, Wuerzburg City Council, City of Wuerzburg<br>Karmelitenstraße 43, 97070 Wuerzburg

	Dr. med. Johann Löw<br>Head of Local Helath Authority (“Gesundheitsamt”) Wuerzburg
	Dr. Barbara Finkenberg<br>Local Helath Authority (“Gesundheitsamt”) Wuerzburg
Biometry and data management	Prof. Dr. med. Peter Heuschmann<br>Viktoria Rücker<br>Julia Schmidt<br>Timo Ludwig<br>Institute for Clinical Epidemiology and Biometry,<br>University of Wuerzburg, Josef-Schneider-Str. 2-D7, 97080 Wuerzburg

1.2 Signatures

Study lead:

Wuerzburg, 2021-03-30

____

Prof. Dr. med. Johannes Liese, MSc
Department of Pediatrics, University Hospital Wuerzburg

Wuerzburg, 2021-03-30

____

Prof. Dr. Oliver Kurzai
Institute for Hygiene and Microbiology, University of Wuerzburg

1.3 Abbreviations

AG	Antigen
AB	Antibody
COVID-19	Corona Virus Disease 2019
ELISA	Enzyme-linked immunosorbent assay
FFP	Filtering Face Piece
KiTa	Daycare center
PCR	Polymerase chain reaction
SARS-CoV-2	Severe Acute Respiratory Syndrome Corona Virus 2
VOCs	Variants of Concern

126 ## 1.4 Synopsis

Study title	Wuerzburg child care study during the COVID-19 pandemic, Part II (Wue-KiTa-CoV 2.0):<br>Surveillance of SARS-CoV-2 infections in daycare centers (KiTas) by means of the submission of mouthwash samples 2x/week for pooled PCR and/or antigen rapid testing at home (pilot study)
Short title	SARS-CoV-2 surveillance in daycare centers in Wuerzburg by means of the submission of mouthwash samples 2x/week for pooled PCR and/or antigen rapid testing at home<br>Acronym: Wue-KiTa-CoV 2.0
Study aims	Evaluation of a surveillance concept with continuous screening for SARS-CoV-2 infections in daycare centers, based on the submission of mouthwash samples 2x/week for pooled PCR and/or antigen rapid testing at home with the following objectives:<br>**Main objective:**<br>*Usability:*<br>- Evaluation of the initial acceptance (initial consent for respiratory sampling)<br>**Secondary objectives:**<br>*Usability:*<br>- Evaluation of long-term acceptance (drop-out rates for respiratory sampling)<br>*Effectivity (as a screening instrument):*<br>- Evaluation of detection rates of SARS-CoV-2 infection in daycare centers overall (children plus child care staff) and for children as a function of local incidence in the corresponding age group.<br>**Further objectives:**<br>*Usability:*<br>- Evaluation of the acceptance in individual participant groups<br>- Evaluation of the long-term psychosocial effects<br>- Evaluation of the acceptance on the part of the children<br>- Evaluation of the test strategy "respiratory sampling (any)" or "submission of mouthwash samples 2x/week" as a long-term concept (6 months, incorporating participant data from the earlier study Wue-KiTa-CoV)<br>- Evaluation of the consequences of the test results for the participants / daycare centers

	Effectivity (as a screening instrument):<br>- Evaluation of detection rates of SARS-CoV-2 infection using different testing strategies.<br>- Evaluation of the proportion of false-positive or false-negative detections in the antigen rapid test for non-professionals (nasal swab) with parallel testing by means of PCR (mouthwash) in asymptomatic participants<br>- Evaluation of the length of time between sample collection and notification of a positive test result for PCR testing<br>- Evaluation of the detection rates of SARS-CoV-2 Variants of Concern (VOCs)<br>- Evaluation of the detection rates as a function of the composition of the daycare center groups (e.g., proportion of study participants, proportion of participants from Group 1).<br>- Determination of the SARS-CoV-2 seroprevalence (as a check for effectivity)<br>*Efficiency (evaluation of economic efficiency):*<br>- Evaluation of the direct costs (material, tests, transport)<br>*Scalability (evaluation of further usability):*<br>- Evaluation for suitability as a strategy for a regional/cross-regional 'roll out', with regard to Usability, Effectivity and Efficiency, cost-benefit assessment
Design	Pilot study (open, multicenter, longitudinal)
Population	Daycare center children aged 2 years and older (excluding after-school care children) who are cared for in predefined daycare centers (crèches and kindergartens) in Wuerzburg, as well as the child care staff at these facilities
Sample size	'Eligible': 9 daycare centers in Wuerzburg (already participated in previous study), with approx. 650 potentially suitable children from 2 years of age and approx. 180 child care staff, organized in approx. 50 daycare center groups.<br>Estimation of the initial number of participants based on the surveillance participation rate for mouthwash sampling (2x/week) as determined in Wue-KiTa-CoV:<br>Children: 67 % of approx. 650 'eligible' children = approx. 436 children<br>A participation rate of 67 % can be estimated from 650 children with a precision of 3.7 % (half-width of the Wilson-score 95 % CI).<br>Child care staff: 86 % of approx. 180 'eligible' child care staff = approx. 155 child care staff<br>A participation rate of 86 % can be estimated from 180 child

	care staff with a precision of 5.3 % (half-width of the Wilson-score 95 % CI).<br>The maximum number of samples to be expected for a screening period of 12 weeks (participation of all potentially eligible 830 individuals in Participant group 1):<br>Approx. 20,000 mouthwash samples and approx. 20,000 documented antigen rapid tests.
Inclusion criteria<br><br>Exclusion criteria	• Daycare center child aged 2 years and older or daycare center child care staff, in one of the participating facilities<br>• Written declaration of consent (parents or child care staff)<br><br>After-school care center children<br>Other daycare center personnel without close contact with the children (e.g. kitchen help, janitor, etc.)
Participant groups, procedure of the study	Parents or child care staff willing to participate in the study select one of the following screening methods in the informed consent form for detection of SARS-CoV-2 infection in asymptomatic participants:<br>**Participant group 1:**<br>2x/week submission of mouthwash samples collected at home for PCR testing<br>AND<br>in parallel 2x/week antigen rapid tests performed at home<br>**Participant group 2:**<br>2x/week submission of mouthwash samples collected at home for PCR testing<br>**Participant group 3:**<br>2x/week antigen rapid tests performed at home<br><br>The choice of groups is left to the participants.<br>Screening of participants via respiratory sampling is conducted for approximately 3 months (expected to be 12 weeks in April-July 2021).<br>Mouthwash samples are dropped off by the parents at the daycare center. They are then transported to the study center by study representatives. The sample submission is documented at the study center and samples are submitted for laboratory testing.<br>The documentation of the mouthwash sampling is carried out by the laboratory as well as by the parents (1x/week, online query).<br>If necessary, SARS-CoV-2 positive PCR results will be communicated to participating parents/child care staff via the

	study center. The laboratory informs the Department of Public Health in case of a SARS-CoV-2 infection being detected. In addition, the day care center receives the information without a name being provided that a positive test has occurred. The Department of Public Health initiates further measures (such as closing the daycare center group or the daycare center, informing those tested positive about quarantine measures, etc.).<br><br>Documentation of antigen rapid testing is done by the parents (1x/week, online query). In the case of a positive test, the study center can be contacted for collection/delivery of additional respiratory samples (throat swab, additional mouthwash if necessary) for prompt retesting with PCR (email, telephone hotline). The laboratory informs the Department of Public Health in case of detection of a SARS-CoV-2 infection in the follow-up testing via PCR. In addition, the day care center receives the information without a name being provided that a positive PCR test has occurred. A control smear test is offered in case of a discrepant test result.<br><br>The following are collected additionally from the participants (after written consent):<br>- Socio-demographic data<br>- Psychosocial aspects (online survey at the beginning/end of the screening period, in addition structured interviews in the case of participant random sampling)<br>- Questioning by the parents of the children on their acceptance of testing (at beginning/end of screening period); parent assessment (online survey)<br>- Seroprevalence testing (antibody detection) at the beginning/end of the screening period. Collection is effected in the daycare centers by medically trained study staff.<br><br>Additional offer for testing of symptomatic participants (PCR from throat swab) in the central SARS-CoV-2 testing center at the University Hospital Wuerzburg with appointment organization via study center (email, telephone hotline).
Laboratory	PCR testing from mouthwash samples for SARS-CoV-2, including 'Variants of Concern' (VOCs):<br><br>For children and child care staff: initially pooled, in case of a positive detection additionally single testing from reserve samples.<br><br>Commercially available antigen rapid tests for self-testing will be provided to study participants.<br><br>Antibody detection by means of point-of-care test (finger prick), if required with retesting (venous blood sampling; ELISA from blood serum)
Endpoints	**Primary endpoint:**<br><br>*Usability:*

	• Initial acceptance rate of the respiratory sampling (Group 1, 2, 3 combined) on the basis of the specifications on the declaration of consent (participant rate of all suitable participants)<br>**Secondary endpoints:**<br>*Usability:*<br>• Participant rate stratified by participant group (1, 2, 3)<br>• Acceptance rate of the respective participant records defined as the share of 'successful' participations in all 'planned' specimen collections (mouthwash and/or nasal swab for non-professionals) among participating asymptomatic children and child care staff of the facility. For an individual participant, participation in specimen collection is considered successful when 60 % of the planned (asymptomatic) specimen collections have been successfully completed.<br>*Effectivity (as a screening instrument):*<br>• Detection rate of SARS-CoV-2 infections in the daycare centers in total per participant/testing (daycare center children plus daycare center child care staff) and in relation to the 7-day incidence in the total population of children in the corresponding age group<br>**Further endpoints:**<br>*Usability:*<br>• Acceptance of the mouthwash sample submission (share of the tests carried out successfully in Group 1 plus Group 2)<br>• Acceptance of the non-professional antigen rapid tests (nasal swab) (share of the tests carried out successfully in Group 1 plus Group 3)<br>• Predicators of the acceptance for longer-term usage<br>• Psychosocial effects, including satisfaction and fulfillment of parents' need for safety (using questionnaires and validated scores) at the beginning and end of the study<br>• In subgroups: Long-term psychosocial effects, including satisfaction and fulfillment of parents' need for safety (using questionnaires and validated scores) including participants of both Wue-KiTa-CoV and Wue-KiTa-CoV 2.0 (6-month evaluation)<br>• Satisfaction of the children (assessment by the parents and child questionnaire in Week 1 and Week 12).<br>• Frequency of consequences in the case of positive tests (daycare center group closures / daycare center closures, total 'workdays lost' due to PCR-confirmed detections, and additional 'workdays lost' due to false-positive antigen rapid tests)

	Effectivity (as a screening instrument):<br>• Detection rate of SARS-CoV-2 infections in daycare centers per participant group (Group 1, 2, 3), separately for children and child care staff<br>• Proportion of all positive PCR tests in total (mouthwash) (Group 1 + Group 2)<br>• Proportion of all positive antigen rapid tests in total (nasal swab) (Group 1 + Group 3)<br>• Comparison of detection rates of SARS-CoV-2 infections by means of non-professional antigen rapid testing (nasal swab) versus PCR (from mouthwash) in parallel testing of asymptomatic participants (Group 1) for identification of false negative detections in the antigen rapid test<br>• Identification of false-positive antigen rapid testing through retesting by means of PCR<br>• Length of time between sample collection and notification of a positive test result for PCR testing.<br>• Detection rates of SARS-CoV-2 Variants of Concern (VOCs), in total and over time<br>• Detection rates as a function of the ‘daycare center’ group composition (proportion of study participants, proportion of Group 1 participants)<br>• Proportion of positive serologic tests overall (children/child care staff), correlation of positive PCR and seroconversion between first and second serologic testing. This is a control for effectivity of surveillance concepts<br>Efficiency (evaluation of economic efficiency):<br>• Costs for material and tests of asymptomatic participants (direct costs)<br>• Quantification of the influence of pooling on the test costs<br>• Costs of retesting positive antigen rapid tests of asymptomatic participants with PCR<br>• Transport costs (sample collection for laboratory)<br>• Parents' estimate of the average time spent sampling<br>Scalability (evaluation of further usability):<br>• Extrapolation of the number of participants, test consequences, and costs for all daycare centers in Wuerzburg<br>• Cost-benefit assessment

	• Modeling of the scalability as a function of participant rate, background incidence, test frequency, detection rate detection method, using state-based modeling methods
Statistical analyses	To estimate the primary endpoints, the 95 % confidence interval is estimated using the Wilson score method. The significance level for all analyses is set to 5 %. Since these are purely exploratory analyses, the significance level is not adjusted for multiple testing. The data are analyzed stratified by daycare center children/parents and child care staff. Initially all end points are described descriptively (frequency (percentage), mean value (SD) or median (IQR)). The development of psychosocial factors is evaluated overall and, if the number of cases is large enough, stratified by the participant group. This is done using tests for connected samples such as McNemar, ANOVA, and Friedmann. Potential predictors on the longer-term acceptance rate (drop-out rate) will be examined overall and stratified by the participant group. First in a descriptive manner using suitable tests such as Chi² test and later, if the number of cases is large enough, using logistic regression (adjusted for the participant groups). The analyses are effected by means of SAS, R or SPSS.
Time line	<u>Study period:</u> 2021-04-01 to 2021-12-31 (9 months)<br>Start of Wue-KiTa-CoV 2.0 if possible directly after completion of the Wue-KiTa-CoV surveys on site in the daycare centers (there until March 2021). Respiratory sample collection for Wue-KiTa-CoV 2.0 in daycare centers is expected to last 12 weeks in the period of April to July 2021.<br><u>Reporting</u>:<br>- Status report April 2021 ('Manual')<br>- Status report May 2021<br>- Status report June 2021<br>- Project report July/August 2021 ("meaningful")<br>- Final report Sep 2021 (provisional; main evaluation)<br>- Final report Dec 2021 (final evaluation)<br>Plus weekly <u>short reports</u> in regards to:<br>- N included children (only during recruitment phase)<br>- N documented tests per week<br>- Cumulative since project start:<br>a) Positive tests (pool/individual tests mouthwash, documented antigen rapid tests)<br>b) If appropriate, correct-positive (after PCR confirmation of antigen rapid tests)<br>c) If necessary, false-negative tests after PCR confirmation (only possible for participant group 1 tested in parallel)

1.5 Flow diagram order of study course/recruitment/data acquisition

Würzburger children support study during the COVID-19 pandemic, Part II:
Surveillance of SARS-CoV-2 infections in daycare centers by means of twice weekly mouthwash submission for pooled PCR and/or antigen rapid testing at home

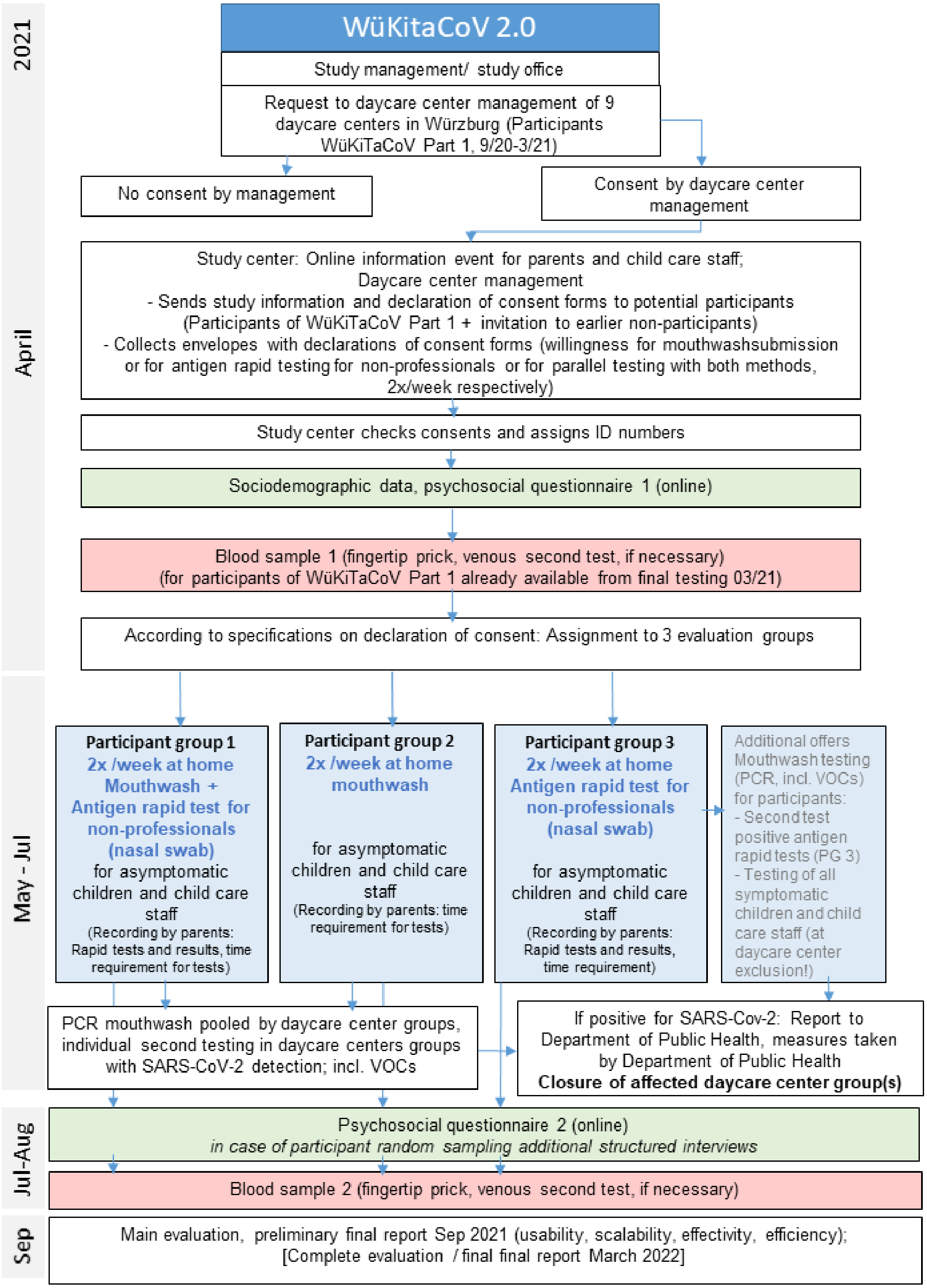

1.6 Background: COVID-19 and childcare

SARS-Cov-2/COVID-19 pandemic

Since December 2019, the new coronavirus SARS-CoV-2 has been spreading worldwide. On March 11, 2020, the WHO officially classified the outbreak as a pandemic. The SARS-CoV-2 virus belongs to the coronavirus family. Human-pathogenic coronaviruses usually cause upper respiratory tract infections in humans. The clinical manifestation spectrum ranges from normal cold symptoms to severe disease courses with possible inflammatory-cardiovascular and neurological implications [1].

The disease caused by SARS-CoV-2 is referred to as COVID-19 ("**Co**rona**vi**rus-**D**isease-20**19**") According to information from the Robert Koch Institute, the courses of the disease are non-specific, diverse, and vary widely – this can range from asymptomatic courses to severe pneumonia with lung failure, multi-organ involvement, and death. From the cumulative cases recorded in the European Surveillance System (TESSy) with symptom information (n = 100,233 from 12 European countries; as of 2020-04-21), the most common symptoms reported are impaired taste and smell, fever, cough, and sore throat [2]. The Epidemiologic Bulletin of 2020-04-23 reported that 8-10% of the cases reported in Germany are hospitalized – with the hospitalization in children being very rare. Overall, children and adolescents are less likely to develop COVID-19 compared with adults. And those who do develop the disease predominantly show mild, uncomplicated upper respiratory tract infections while severe complications are rare [3].

After an initial spread of the virus in Asia, the WHO European Region has been the epicenter of the pandemic since mid-March 2020. The European Region accounted for 63 % of global mortality due to the virus as of April 28, 2020 [4]. Meanwhile, the majority of new diagnoses are made in Russia, Central and South America, and the United States. The epidemiological situation in Africa is largely unclear.

Since fall 2020, the worldwide and increasing spread of several SARS-CoV-2 virus variants of concern ("Variants of Concern," VOCs) with increased transmissibility and, in some cases, still unknown virulence has been observed. This further complicates the epidemiological situation (www.rki.de).

The global outbreak of SARS-CoV2 has made dramatically clear that new infectious diseases can present significant and drastic challenges at any time, even to industrialized countries with excellent medical infrastructure. The fight against the pandemic in Germany was accompanied by drastic measures that had a significant impact on people's daily lives. Examples of such measures are the cancellation of major events, substantial targeting of inpatient care towards COVID-19, worldwide travel warnings issued by the German Federal Foreign Office, widespread curfew and contact restrictions, widespread closures of stores and catering establishments, and the introduction of compulsory face masks in many areas of public life.

Effects of the COVID-19 pandemic on childcare

The measures affecting the care of children are particularly drastic. They have significant psychosocial consequences both for the children and for the families. Relevant professional associations therefore pointed out that the closure of daycare centers, children's playgrounds, schools and sports facilities results in the deprivation of essential prerequisites for healthy physical, mental and social development [5]: It has been argued that, since the course of the disease in children and adolescents is usually mild, school closures are not primarily intended to protect children but to contain the pandemic and ultimately to protect the vulnerable elderly population. It has also been pointed out that school closures contradict children's right to education (United Nations Convention on the Rights of the Child, Article 28) [6]. In particular, in view of the need to simultaneously care for children at home and meet work obligations while working remotely, parents are also sometimes unable to adequately fulfill either their professional responsibilities or care for their children, or both. As a result, the closure of childcare facilities and schools also has a significant economic impact. Furthermore, in many places support and assistance services (youth welfare offices or social institutions) are largely absent for families because of the distances involved. Outreach services can no longer be guaranteed. Youth welfare offices cannot adequately fulfill their role as guardians of children. And there is an increased risk that children's well-being will be endangered.

Clinical manifestation of SARS-CoV-2 infection in children

SARS-CoV-2 infection usually results in milder clinical symptoms in children than in adults:

A systematic review based on 14 publications from China showed that of 2228 children, only 6 % had a severe course (72 % mild, 22 % moderate). Only two deaths were described in the papers analyzed (one 14-year-old child, one newborn) [7]. A further systematic review based on 45 published case series and case reports shows that children account for 1-5 % of the diagnosed COVID-19 cases and generally have a milder disease course than adults. Deaths in children were only found in isolated cases in this work as well [8].

A multi-systemic inflammatory disease (Kawasaki-like syndrome) with circulatory dysfunction and macrophage activation syndrome has been described in children as a rare severe complication of a SARS-CoV-2 infection [9,10].

Also first analyses from Europe (Italy) confirm a generally mild course of SARS-CoV-2 infection in children [11]. In an analysis of 168 laboratory-confirmed pediatric cases (median: 2.3 years), the majority of whom were hospitalized (67.9 %) and had comorbidities to a high proportion (19.6 %), only two of the children received intensive care.

In Germany, 0.1 % of all recorded COVID-19 cases were between 0 and 4 years old, and 0.23 % were between 5 and 14 years old. The age-related incidence for 0 to 14-year-olds ranged from 45 to 57/100,000 population. It was thus significantly lower than in all other age groups (167-384/100,000 population) [12]. The Robert Koch Institute (RKI) concludes that in the majority of available studies, children are less likely to be affected by a SARS-CoV-2 infection than adults. According to the RKI, the symptoms of the disease often seem to be less pronounced in children than in adults. And according to initial studies, asymptomatic courses are also relatively common (in up to 28 % of the cases).

Role of children and daycare centers in the spread of SARS-CoV-2

The role of children in the spread of SARS-CoV-2 has not yet been finally clarified. In reports of clusters in France and in Australia, no transmission from infected children to further adults or children was observed [13,14]. In a large surveillance study in Iceland, children were affected less frequently in both a risk population (6.7 versus 13.7 %) and a population screening (0 % vs. 0.8 %) than adults [15]. Similar results were seen in the study of 2812 residents of the town of Vo (Veneto, Italy) during an outbreak: There was no case here in children younger than 10 years, while SARS-CoV-2 was detected in 2.8 % of adults [16]. In a Japanese study examining suspected cases and contacts, the attack rate is lowest in the age groups of 0-10 and 10-20 [17]. Observations from Sweden, where child care was restricted and yet children are underrepresented among those detected infected, may also indicate a lower rate of infection among children. Authors of a systematic review concluded - with a poor data base - that school closures made little contribution to combating the SARS-CoV-2 pandemic [18].

Other analyses show, however, that children are infected with the same frequency as adults [19]. Early in the course of the pandemic, it was also suggested that children might be efficient carriers of SARS-CoV-2 because of a lack of symptoms and concurrent viral replication. Appropriately, the efficient spread of SARS-CoV-2 in a school in France was described [20]. In this school with predominantly older pupils, the attack rate was 40.9 % for students/teachers and 10.9 % for relatives. Analysis of viral detections from respiratory materials did not indicate that the viral load in the upper airways of children with evidence of SARS-CoV-2 is quantitatively lower than that of adults to a relevant extent [21]. The authors therefore doubt a lower infectivity of children. They do indicate, however, that due to the lower respiratory volume and the lower force of the cough bursts of infants, the infectivity of children might be lower despite the same viral load.

Overall, the prevailing view is that children usually show asymptomatic or predominantly mild courses of COVID-19 [6]. Whether they are also carriers of SARS-CoV-2 more rarely has not yet been determined. A modeling study on the effectivity of sociopolitical control and abatement measures against SARS-CoV-2 transmission in 41 (mostly European) countries, initially published as a pre-print in June 2020, comes to the surprising conclusion that - compared with other measures such as business closures and curfews - school closures were, on average, the most effective measure for reducing the transmission rate [22].

Measures to fight the COVID-19 pandemic in child care settings

All schools and daycare centers in Bavaria were closed on March 16, 2020 to contain the spread of SARS-Cov-2. Basis is the assumption that oligo-symptomatic and asymptomatic children are also infectious and contribute to the spread of the virus in the community by attending community facilities. The scientific basis for this is almost exclusively based on analyses of influenza outbreaks and may not be applicable to the SARS-CoV-2 situation [6].

After the state-wide closures, only emergency care was maintained – which was available to a widely varying extent. The regulations governing the right to use emergency care have also been changed multiple times over time. Facilities in which SARS-CoV-2 has been detected have as a rule been closed by the respective Department of Public Health for at least two weeks. Reopening of daycare centers took place step-by-step in May, June and July 2020. The reason for the hesitant reopening is, in particular, that common and proven protective measures cannot be implemented in daycare centers. This encompasses, in particular,

systematically maintaining a distance of 1.5 m from other people as well as wearing masks covering mouth and nose coverings – especially in situations where distancing is not possible. Neither of these measures can be reasonably implemented – especially when it comes to the care of young children.

Since the implementation of hygiene concepts is very difficult in childcare facilities, the effective and reliable surveillance of children in these facilities plays a significant role. However, even close monitoring approaches that are simple and feasible for adults cannot be applied directly to children. This is because of their lack of comprehension and cooperation. Various concepts for effective monitoring of children in childcare facilities in Germany are currently being developed (Hübner et al. Teststrategien zur COVID Diagnostik in Schulen [Test strategies for COVID diagnostics in schools], 2021-02-28; https://dgpi.de/publikationen/covid-19-publikationen/). Depending on the prevalence of COVID-19 in the population, such measures may need to be implemented extensively and over longer periods of time in the future. In addition to the high effectivity of a monitoring measure for the detection of SARS-CoV-2 infections, the longer-term acceptance, in addition to the initial acceptance, of the measure by all stakeholders (children, family, child care staff, funding institution) is crucial here. A theoretically highly effective measure for the detection of infections, such as daily swabs of all daycare center children and testing by means of PCR, will not lead to the desired success if there is a lack of acceptance by those involved. Conversely a method that is expected to have a high level of acceptance, such as surveillance, not of the daycare center children themselves but of their household members for respiratory symptoms, or the use of (possibly less sensitive) saliva tests, could also show too little effectivity with regard to the goal of preventing the spread of infection in the daycare center, Therefore the feasibility and acceptability of various potential monitoring measures should first be evaluated before evaluating the effectivity.

At the end of February 2021, the first antigen (AG) rapid tests for self-testing "for non-professionals" for SARS-CoV-2 were approved in Germany. Those tests detect infectious individuals well within approximately 15-30 min, especially in the case of high viral loads. Their usage for extensive screening of asymptomatic children in schools and childcare facilities (KiTas) is, however, controversial, due to the expected unacceptably high numbers of false negative and false positive results. It is rejected by the leading pediatric societies in Germany and extensive use is only recommended under close scientific monitoring (Hübner et al. Teststrategien zur COVID Diagnostik in Schulen [Test strategies for COVID diagnostics in schools], 2021-02-28; https://dgpi.de/publikationen/covid-19-publikationen/). Furthermore, in this position paper the pediatric societies recommend the scientific evaluation of innovative concepts, such as pool testing of respiratory materials, which can be obtained in an uncomplicated way by non-professionals, and their evaluation also with regard to logistic feasibility and cost-effectivity for PCR-based testing strategies (highest specificity and sensitivity).

Objective of the previous feasibility study (Wue-KiTa-CoV)

In the feasibility study 'Wue-KiTa-CoV' funded by the German Federal Ministry of Education and Research (BMBF) as part of InfectControl 2020 (CoVMon 03COV16A project; study direction Prof. Kurzai and Prof. Liese), four different PCR-based concepts for the surveillance of SARS-CoV-2 infections and their spread in daycare center children and child care staff are tested for acceptance by the participants and their practical feasibility and compared with

each other: in Module 1, 2x/week resp. in Module 2 1x/week a nasal swab (mid-turbinate swab) is performed by medically trained study staff in asymptomatic participants in daycare centers, in Module 3 2x/week mouthwash is submitted in daycare centers for PCR testing, in Module 4 no testing is performed in daycare centers, but symptomatic daycare center children, child care staff and their household members are offered PCR testing by means of throat swab. In addition to the acceptance of the respective measures and their technical and logistical implementation, the psychosocial impacts of the various surveillance protocols will also be assessed through accompanying interviews with children, families, and care staff. And the cost per daycare center child is calculated on the basis of direct costs (material, personnel, testing), and seroprevalence tests for SARS-CoV-2 antibodies are performed.

The overarching goal of the Wue-KiTa-CoV feasibility study is to identify surveillance concepts with good acceptance.

A total of 9 daycare centers in the Wuerzburg area are involved. The practical implementation in the daycare centers in Wuerzburg began in October 2020 and will be completed in March 2021. The study period included 3 months of respiratory sampling. The last respiratory sampling occurred at the end of February 2021. The main evaluation phase begins in April 2021. The first data on acceptance are available.

First results were submitted as congress abstracts at the end of February/beginning of March 2021. In total the parents of 442 (57 %) of 772 eligible daycare center children (1-6 years), and 150 (82 %) of 182 child care staff agreed to participate in the Wue-KiTa-CoV respiratory surveillance. The highest initial acceptance was for mouthwash collection (67 % of parents, 86 % of child care staff). A supplemental survey of nonparticipants revealed the most common reason for declining study participation or respiratory sampling was the fear of a negative experience for the child. Only 3 % of nonparticipants expressed 'Corona skepticism'.' A relevant proportion of the child care staff expressed strong concern about infecting others with SARS-CoV-2 (42 %) or becoming severely ill with it themselves (20 %). The drop-out rate amounted to less than 5 % during the 12-week screening period in the mouthwash sampling. In all modules, 70 %-90 % of participants indicated a high or very high willingness to continue surveillance, if necessary, using 2x/week mouthwash sample collection.

Motivation for the pilot study proposed here (Wue-KiTa-CoV 2.0)

The practical implementation of Wue-KiTa-CoV in the daycare centers comes to an end at the end of March. In the follow-up study Wue-KiTa-CoV 2.0, the practical implementation of respiratory sampling will be resumed again in the relevant Wuerzburg daycare centers from April 2021. This will happen while retaining most of the accompanying examinations, however, with a new test concept based on the results of the first study.

Wue-KiTa-CoV 2.0 …

a) ... Is based on the initial participant preference shown in Wue-KiTa-CoV for mouthwash submission for PCR as a screening tool in daycare centers,

b) .... In addition addresses the current situation regarding the availability of non-professional antigen rapid tests, which changed at the end of February 2021,

c) ... Also addresses the current recommendations of the pediatric specialty societies (2021-02-28) regarding the evaluation of innovative strategies (pooling, easy-to-obtain respiratory samples),

d) .... Considers the changed situation since December 2020 regarding the rapid spread of VOCs also now in Germany.

e) ... May also evaluate, if appropriate, the initial impacts of the changed situation regarding the rapid SARS-CoV-2 vaccination of daycare center staff. Vaccinations have been possible since the end of February 2021.

Wue-KiTa-CoV 2.0 is intended to …

... Evaluate in a comparative approach 2x/week mouthwash collection and/or 2x/week antigen rapid testing 'for non-professionals';

... In the process initially analyze mouthwash samples as pooled by means of PCR, and only if the pooling result is positive should reserve samples be retested individually by means of PCR.

... Record the incidence of SARS-CoV-2 infections including VOCs in daycare centers, absolutely and in children in relation to the background incidence of the corresponding age group;

... Evaluate the psychosocial effects of the vaccinations of child care staff.

Overarching target criteria are the evaluation in terms of "Usability", "Effectivity", "Efficiency" and "Scalability" (broader applicability).

In regards to the last point, a "manual" for usage in the daycare centers outside of a study should also be created. This could help to prepare the planning of a possible follow-up study in case of a favorable evaluation of the target criteria (if necessary, scientific support of a "roll-out" of the suitable test strategy in trans-regional application).

Objective of a possible subsequent study

In the case of a regional or trans-regional 'roll-out' decided by the health authorities based on the results of the pilot study, the chosen surveillance concept should be further optimized and further reviewed in regards to its effectivity in the early detection of SARS Cov-2 infections in daycare center children. The ultimate goal is to interrupt the spread of the virus in the daycare centers at an early stage, thus maintaining child care in the daycare centers as continuously as possible. For example, daycare centers in regions without implementation of a surveillance concept could serve as a control group in a 'roll-out'.

1.7 Questions Wue-Kita-CoV 2.0

The following question is to be answered in particular as part of the pilot study described below:

I. Do the children in care, their parents and the child care staff accept the SARS-CoV-2 surveillance approaches which are based on regular, easy-to-perform self-administer sampling of child care staff and daycare center children at home? Both initially and in longer-term implementation ('Usability')? To what extent does the acceptance differ between mouthwash collection for PCR testing and antigen rapid testing for non-professionals?

II. How effective are such approaches in terms of detecting SARS-CoV-2 in daycare centers, depending on the type of testing used and the background incidence ("Effectivity")?

III. What is the cost-benefit ratio, especially under the inclusion of pooling of mouthwash samples for PCR analyses ("Efficiency")?

IV. How suitable are such concepts for trans-regional application ("Scalability")?

In addition, the following questions are, amongst other, addressed:

V. How frequently can SARS-CoV-2 VOCs be detected in asymptomatic participants - do VOCs show altered propagation behavior in daycare centers?

VI. How often does systematic testing of asymptomatic daycare center children by parents using the antigen rapid non-professional tests lead to false-positive or false-negative results, compared to mouthwash pooling (at a positive test followed by individual testing)?

VII. How much does the detection rate depend on the percentage of surveillance participants in the daycare center group?

VIII. What sociodemographic factors are potential *predictors of longer-term participant acceptance* for surveillance approaches to child care institutions?

IX. What are the *psychosocial effects* of long-term infection monitoring on children, their families, and child care staff?

X. How high is the *SARS-CoV-2 seroprevalence* among children and child care staff in Wuerzburg and how does it change over the study period?

XI. If appropriate: Does vaccination lead to an individually higher sense of safety and satisfaction for child care staff?

1.8 Risk-benefit ratio

Overall, the risks of the study for the participants are to be considered to be low.

The samplings are examinations in which normally only minor side effects are to be expected. This includes an unpleasant sensation during the nasal swab (antigen rapid test) or a possible (optional) throat swab and a small hematoma resulting from the fingertip prick.

Risks when collecting the nasal swab:

Relevant complications of nasal swab collection in antigen rapid tests approved for self-testing are very rare. There may be an unpleasant sensation when taking the sample. It is an objective of this study to determine to what extent the repeated collection of nasal swabs is tolerated by the children / child care staff. These are subjectively perceived as unpleasant in very different ways (see Study objectives).

Risks when collecting the mouthwash samples:

No relevant risks arise here, since only drinking water is used for rinsing, if necessary.

Risks associated with fingertip prick (additional measure):

In rare cases a hematoma may occur at the prick point and, very rarely, infection of the prick point may occur.

Risks associated with venous blood sampling (pertains only to children/child care staff with abnormal and borderline point-of-care diagnostic findings from the fingertip):

- **Bruising (hematoma)** at the prick site due to further injury of the pricked vein or surrounding vessels
- In rare cases, **injury to arteries** and resulting bleeding
- In very rare cases, **infection** of the prick point
- In very rare cases, **injury to nerves** with loss of sensation and/or movement, and a very low risk of permanent damage.

All blood collections are performed by medical personnel under the supervision of a physician.

Infection risk through SARS-CoV-2 for the study population

The initial infection risk of the study population is identical to that of all children cared for in daycare centers in Wuerzburg. The implementation of the surveillance concepts is expected to result in earlier and more efficient detection of emerging cases. This may result the in secondary risk of infection (infection through transmission of SARS-CoV-2 in the daycare from an unrecognized infected person) possibly being lower for the study population than in facilities not participating in the study.

The epidemiological situation in regards to the spread of SARD-COV-2 is monitored at all times. Any detection of SARS-CoV-2 in a sample collected as part of the study will be reported to the appropriate Department of Public Health in accordance with regulatory requirements. The Department of Public Health makes the decision in each case to what extent quarantine measures and closures of the daycare or individual groups in the daycare are necessary. If the virus is detected in one of the study daycare centers, the parents and child care staff will be informed immediately about the need for close self-observation and immediate testing in case of symptoms. An advisory hotline by telephone in the study center is available for all custodians and child care staff.

No additional risk is created to and in the daycare centers through the testing conducted as part of the study, as all sampling/testing, which may involve infectious aerosols, is conducted

at home. Mouthwash samples will be transported in suitable transport containers for potentially infectious material by parents to the daycare centers, where they will be collected for laboratory testing. Testing is performed in a timely manner to ensure notification in the event of infection prior to the next scheduled daycare visit. The used non-professional antigen rapid tests should be disposed of by the parents in accordance with the manufacturer's instructions.

As part of the study, all symptomatic children and child care staff who are excluded from attending daycare centers due to the Bavarian hygiene framework for daycare centers will also be offered testing by means of throat swab at the testing center at the University Hospital of Wuerzburg.

Benefit:

So far there is no foreseeable end to the SARS-CoV-2 pandemic. Predictable, reliable, and robust child care plans must be developed accordingly. These concept variants can support decisions regarding the installation of measures in the event of renewed local or trans-regional foci of infection.

In the present pilot study, different surveillance concepts are compared in asymptomatic daycare center children or child care staff. In addition, all symptomatic participants (daycare center children and child care staff) will also be offered throat swab testing if defined symptoms are present. This will make it possible to compare the feasibility and acceptability of the different approaches. The aim is then to develop optimal concepts for regional and trans-regional surveillance of daycare centers during the SARS-CoV-2 pandemic in the medium and long term and to further evaluate their effectivity. The results also allow the definition of realistic surveillance concepts for future pandemic respiratory viruses.

Over its course the study will compare mouthwash submissions for PCR testing with commercially available antigen rapid tests for home ("non-professional") testing. These antigen rapid tests show a false-positive result in about 1-2 % of the tests. With the effect that daycare center children and daycare center child care staff are unnecessarily discouraged from attending the daycare center. The study here allows a better estimation on the effectivity of the use of such rapid tests in asymptomatic individuals.

The systematic testing means that there is an increase in the overall safety for the daycare center children as well as for the child care staff. The availability of medical contact persons within the framework of a study hotline, ensures the greatest possible safety for the custodians and child care staff.

Of particular importance is the fact that the study design, through early detection of SARS-CoV-2 infection, may allow child care to be upheld even if an isolated case occurs. Although the decision to close a facility is made solely by the Department of Public Health department independently of the study, the likelihood of spontaneous center closures which can result in uncertainty and need for child care at home can be reduced.

In order to maintain child care initially when an individual case occurs, the following procedure of the local Department of Public Health would be conceivable, for example, as a model upon the first detection of an infection in a participating daycare center in the context of this surveillance study:

Department of Public Health:

- Orders appropriate isolation and quarantine measures for the affected child in accordance with currently existing regulations,

- The affected daycare center group and all other daycare center groups of the facility remain open for the time being

Possible modifications of the study surveillance for this situation would be:

The test frequency for any additional study participants from the affected daycare group will be increased in the following 2 weeks (3 instead of 2 tests/week, also additional smears if necessary). Test frequency for study participants from other daycare center groups will not be increased.

- The non-study participants from the affected daycare center group will be offered additional testing (swabs)

Department of Public Health:

- Only in the event of further children or child care staff testing positive at the same time or in succession would a decision then be made about any additional testing and quarantine measures that may be necessary. This includes daycare center group closures/daycare center closures.

Such an approach would require that the Department of Public Health informs not only the management of the daycare center but also the parents of all daycare center children and the daycare center child care staff in advance about the occurrence of the case and the intention of keeping open of the affected daycare center group. This would ensure that everyone can decide individually to suspend daycare attendance or care work for the time being, if necessary, due to their own concerns. At this moment, it is not yet clear whether legal certainty exists for such a possible approach by the Department of Public Health. If the Department of Public Health adopts such an approach or a similar one, a timely adjustment to the study protocol/surveillance regarding testing frequency/additional swabs would be possible in this regard.

From an infection epidemiology perspective, a potential benefit of the pilot study is also the early identification of potentially infectious children/child care staff. This allows chains of infection to be interrupted efficiently and prevents further illnesses.

As a result of the study, it will also be possible to make initial statements about which of the investigated surveillance concepts for childcare centers are suitable for longer-term use, in terms of acceptance, practical feasibility, effectivity, and efficiency. In addition, a 'manual' (in the sense of "Standard Operating Procedures") is to be developed, which could be suitable for the regional and trans-regional application of the surveillance concepts in the field of public health ("roll out").

2 STUDY OBJECTIVES

In the pilot project Wue-KiTa-CoV 2.0, different surveillance concepts (mouthwash collection, antigen rapid tests for home use) for the early detection of SARS-CoV-2 in daycare centers are being evaluated as examples in the Wuerzburg area. Central elements of surveillance are sample collection/testing 2x/week of asymptomatic daycare center children (by the parents at home) and daycare center child care staff, as well as the testing of mouthwash samples initially pooled, with subsequent individual testing only if the pool is positive. The surveillance concepts will be evaluated primarily with respect to "usability" (assessment of suitability for use). This is the case in particular for initial and longer-term acceptance, and their "effectivity" as a screening tool for the early detection of SARS-CoV-2 infections.

Additionally, potential predictors of longer-term acceptance as well as the psychosocial impact of permanent monitoring on children, families, and child care staff will be examined in regards to “usability”. Other objectives include evaluation in terms of cost-effectivity ("efficiency”). This means with regard to the relevant cost factors associated with each surveillance protocol, as well as the evaluation of broader applicability ("scalability"). In addition, further data will also be collected, for example frequency of detection of VOCs in daycare center children, seroprevalence data on daycare center children in the Wuerzburg area (as a control for the "effectivity" of testing). Data will also be collected on the proportion of child care staff vaccinated against SARS-CoV-2.

2.1 Primary hypothesis

[1] Usability: Screening for SARS-CoV-2 of asymptomatic children and child care staff in childcare centers (2x/week, by mouthwash and/or antigen rapid test) is initially accepted by the majority of parents/child care staff.

2.2 Secondary / further hypotheses

[2] Usability: Screening for SARS-CoV-2 of asymptomatic children and child care staff in childcare centers (2x/week, by mouthwash collection and/or antigen rapid test) is accepted by participants without a relevant drop-out rate over several months.

[3] Effectivity: Screening for SARS-CoV-2 of asymptomatic children and child care staff in childcare centers (2x/week, by mouthwash and/or antigen rapid test) allows early detection of SARS-CoV-2 infection in daycare centers.

[4] Effectivity: Pooling of mouthwash collection (PCR) with single testing in case of a positive pool is a more reliable method for early detection of SARS-CoV-2 infection in daycare centers than antigen rapid self-testing tests

[5] Usability: A long-term screening for SARS-CoV-2 of asymptomatic children and child care staff in childcare centers (2x/week, by mouthwash and/or antigen rapid test) increases the perception of safety and satisfaction among children, their families and the care staff.

2.3 Study design and time line

2.3.1 Design

Pilot study (open, multicenter, longitudinal)

2.3.2 Time line

In April 2021, information will be provided to staff and parents in the daycare centers already participating in Wue-KiTa-CoV and consent will be obtained for Wue-KiTa-CoV 2.0. The implementation is expected to start after the Easter holidays, and in parallel in all facilities. Practical implementation of the study in the daycare centers is expected to end at the beginning of the Summer vacations (Aug 2021). This will probably be after 12 weeks under surveillance.

The following bar chart provides an overview of the details of the study schedule and for the individual work steps.

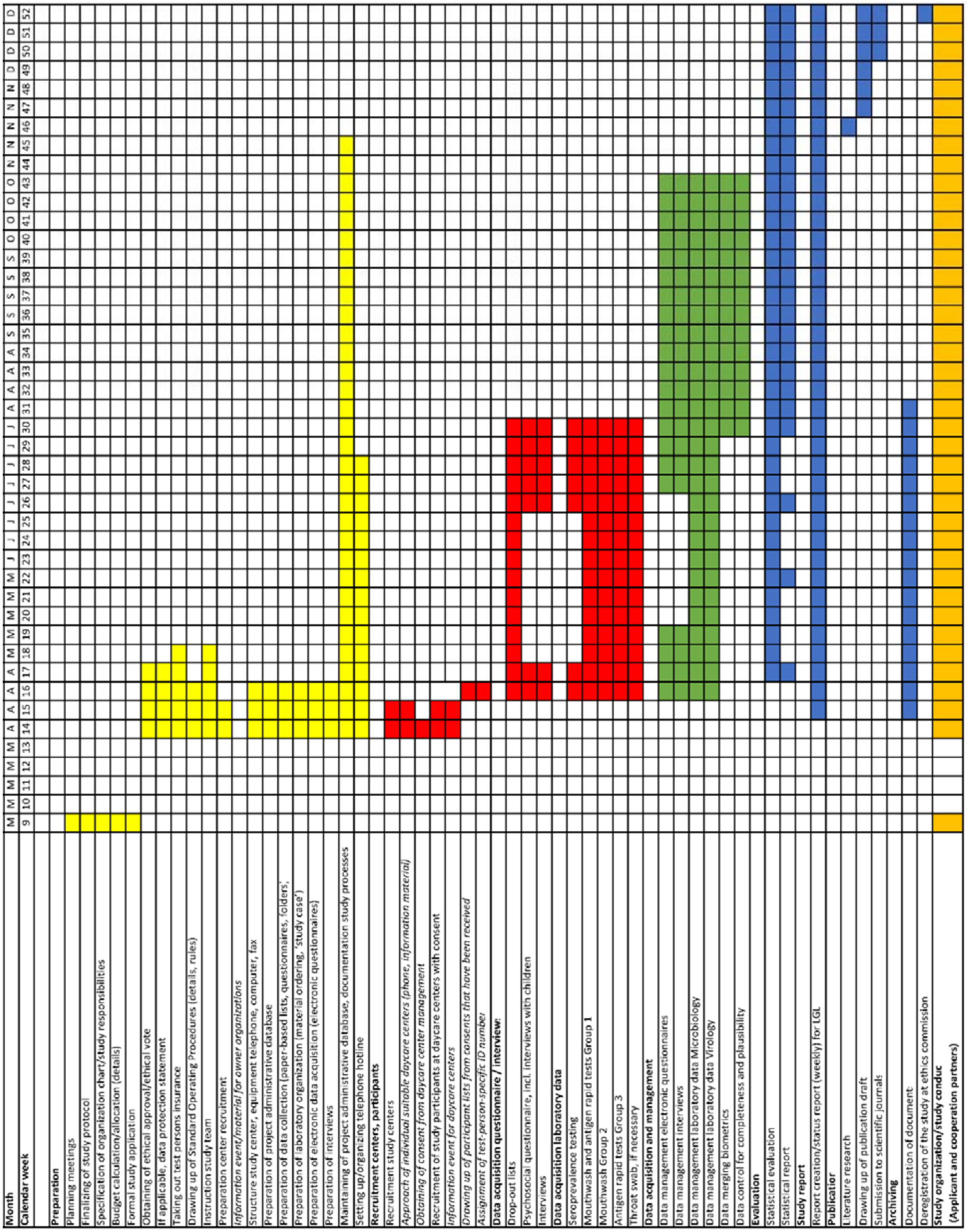
Month
M M M M M A A A A M M M M M J J J J J J J J A A A A S S S S S O O O O N N N N N D D D D
Calendar week
9 10 11 12 13 14 15 16 17 18 19 20 21 22 23 24 25 26 27 28 29 30 31 32 33 34 35 36 37 38 39 40 41 42 43 44 45 46 47 48 49 50 51 52
Preparation
Planning meetings
Finalizing of study protocol
Specification of organization chart/study responsibilities
Budget calculation/allocation (details)
Formal study application
Obtaining of ethical approval/ethical vote
If applicable, data protection statement
Taking out test persons insurance
Drawing up of Standard Operating Procedures (details, rules)
Instruction study team
Preparation center recruitment
Information event/material for owner organizations
Structure study center, equipment telephone, computer, fax
Preparation of project administrative database
Preparation of data collection (paper-based lists, questionnaires, folders)
Preparation of laboratory organization (material ordering, 'study case')
Preparation of electronic data acquisition (electronic questionnaires)
Preparation of interviews
Maintaining of project administrative database, documentation study processes
Setting up/organizing telephone hotline
Recruitment centers, participants
Recruitment study centers
Approach of individual suitable daycare centers (phone, information material)
Obtaining of consent from daycare center management
Recruitment of study participants at daycare centers with consent
Information event for daycare centers
Drawing up of participant lists from consents that have been received
Assignment of test-person-specific ID number
Data acquisition questionnaire / interview:
Drop-out lists
Psychosocial questionnaire, incl. interviews with children
Interviews
Data acquisition laboratory data
Seroprevalence testing
Mouthwash and antigen rapid tests Group 1
Mouthwash Group 2
Antigen rapid tests Group 3
Throat swab, if necessary
Data acquisition and management
Data management electronic questionnaires
Data management interviews
Data management laboratory data Microbiology
Data management laboratory data Virology
Data merging biometrics
Data control for completeness and plausibility
Evaluation
Statistical evaluation
Statistical report
Study report
Report creation/status report (weekly) for LGL
Publication
Literature research
Drawing up of publication draft
Submission to scientific journals
Archiving
Documentation of document
Deregistration of the study at ethics commission
Study organization/study conduc
(Applicant and cooperation partners)

3 STUDY POPULATION

3.1 Procedure for the selection of facilities

The study will be conducted at a total of 9 childcare facilities of the approximately 80 corresponding facilities in the city of Wuerzburg that have already participated in the Wue-KiTa-CoV study. For logistical reasons, the pilot project preferentially included larger facilities (≥ 50 children in care). The selection of the 9 facilities for the Wue-KiTa-CoV study was made in cooperation with the Youth, Family and Social Services Department of the City of Wuerzburg. In addition to the size of the facility, the selection criteria also included the approval of the facility management for the carrying out the study.

3.2 Inclusion criteria for test subjects at the facilities

- Daycare center children from 2 years of age (excluding after-school care children) who are cared for at one of the predefined daycare centers in Wuerzburg as well as child care staff at these centers
- Written consent of both parents/custodians or child care staff for 2x/week sampling (mouthwash samples, antigen rapid tests) of the daycare center child or child care staff, and documentation of sampling
- Optional: Written consent on further surveys (online surveys, and, if necessary, telephone interview, seroprevalence testing)

Exclusion criteria for test subjects at the facilities

- Written consent missing
- Children aged <2 years at the start of the study, after-school children
- Other daycare personnel without regular close contact with the daycare center children (such as kitchen help, janitor, etc.)

Remarks

- SARS-CoV-2 vaccinations of child care staff are NOT an exclusion criterion (vaccinations are queried at the beginning and end of the study; vaccination status is considered in the analysis of seroprevalence data).

- Previous known SARS-CoV-2 disease in daycare center children or child care staff is NOT an exclusion criterion (will be queried at the beginning of the study; previous SARS-CoV-2 disease will be considered in the analysis of seroprevalence data).

- Children with pre-existing conditions are NOT excluded from the start.

4 ORDER OF STUDY

4.1 Recruitment of study centers and test persons

The study is conducted in 9 selected daycare centers in Wuerzburg. The selection of daycare centers (independent of the organization supporting the facility) took place as part of the previous study (Wue-KiTa-CoV) in close coordination with the social services department of the city of Wuerzburg after previous coordination with management and the owners. Potentially suitable daycare centers were pre-selected at that time and management was informed. The criteria for the selection of facilities at that time are listed under 3.1. If one of these 9 daycare centers does not want to participate in Wue-KiTa-CoV 2.0, no further center is recruited.

Participating daycare centers will receive a short questionnaire at the beginning of Wue-KiTa-CoV 2.0 to verify previous information on the structure and organization of the daycare center (number of children cared for, number of child care staff, number of groups, group size, number of child care staff / group, number of rooms, floor space, special hygiene concepts due to SARS-CoV-2).

The general aim is to recruit all children / care groups of a daycare center as subjects for Wue-KiTa-CoV 2.0, if possible. An isolated recruitment of individual children or individual groups does not make sense because the interaction and the use of common areas as well as a possible stigmatization of individual test subjects. All potential participants (including the previous participants of Wue-KiTa-CoV) will be contacted in writing regarding possible study participation. If initially less than 30% of the potential participants in one of the intended daycare centers would like to participate in the screening program, the study will be discontinued at this daycare center after this initial acceptance rate has been determined (discontinuation criterion). This is because a well-founded scientific evaluation of the screening program does not appear possible if only a few individual persons participate. (After the results of the previous study, this is considered unlikely in the current situation, because the majority of previous participants in a preliminary survey were very motivated to participate in continued surveillance using 2x/week mouth water rinsing collection, see Page 20/21).

In addition, online information events about the study are provided. On a study website corresponding information and videos with instructions for the planned sample collection trough the parents are stored.

If possible, the study is to be conducted at the predefined 9 daycare centers for the full 12-week period. The start of sampling should occur simultaneously, if possible, at all daycare centers after participant recruitment is complete. This way sampling at each center can occur during the same 12-week observation period (this is necessary because of the very dynamic nature of SARS-CoV-2 infections).

4.2 Information and consent

After approval by the owners and management of the daycare center to conduct the study Wue-KiTa-CoV 2. 0, information/consent forms on the planned study will be handed out to all families and child care staff by the daycare center management. The parents/custodians and

child care staff will be asked for consent to participate in the study (in addition separate consent parts for the participation in (i) respiratory sampling including its documentation, (ii) additional blood sampling for seroprevalence testing, (iii) for participants in the previous study, consent to include the already available data in the current study. Both parents/custodians of a child must consent to the sampling. Parents/custodians are asked to complete the consent forms and return them to the daycare center and to hand them in in a sealed envelope pre-addressed to the study center in the daycare center.

Participation is voluntary; parents and/or child care staff do not have to inform the daycare center about participation/non-participation in the study (exclusion of a possible disadvantage/discrimination). No information on the participation or findings with name will be shared with the daycare center by the study center.

If written consent is provided, basic sociodemographic data will be collected from participants (e.g., age and gender of daycare center child/child care staff, number of persons in the household of daycare center child/child care staff, parents' highest school-leaving qualification, etc.). In addition, some basic attitudes toward the SARS-CoV-2 pandemic (personal assessment of the danger of the pathogen, attitude toward SARS-CoV-2 vaccination; for child care staff, SARS-CoV-2 vaccination status) are also queried.

Study staff will transport the sealed envelopes containing the consent forms to the study center. The documents are registered and archived at the study center. Each household/participant of the household is assigned a study ID number (pseudonymization). The parents of siblings in the daycare center receive a written notification to which daycare center child the respective ID number is assigned. The subsequent assignment of the pseudonymized online surveys to the respective child are therefore possible.

The share of potential study participants (children, child care staff) who do not participate because of missing consent is recorded.

The written consent for respiratory sampling and, if applicable, serological testing (blood sampling at the beginning and end of the study (see 6.4.3)), are recorded. Participation in surveys, interviews, or the serologic tests is not a mandatory requirement for the further participation in the respiratory surveillance.

For participating parents, child-friendly information about the aims and contents of the study will be provided in the respective surveillance groups, as far as this is reasonable and possible according to the age of the children.

4.3 Interview and clinical examinations

As part of the study three screening approaches (4.3.1) for surveillance of SARS-CoV-2 for asymptomatic daycare center children and child care staff are compared. The examinations take place simultaneously over a 12-week period in all facilities. Regardless of the type of screening, participating children, parents, and child care staff are interviewed regarding the psychosocial implications of the respective strategy (4.3.1), and children and child care staff who have also consented to blood collection will have their immune status assessed with regard to SARS-CoV-2 by serologic testing.

4.3.1 Screening concepts (SARS-COV-2)

4.3.1.1 Group 1: Mouthwash sample submission (for PCR) 2x/week plus simultaneously 2x/week nasal swab for antigen rapid testing at home

From all asymptomatic daycare center children and child care staff participating in Group 1, a mouthwash sample and a nasal swab is taken 2x/week at home (by parents in the case of children) and tested for SARS-CoV-2 (antigen rapid test at home according to manufacturer's instructions, mouthwash after submission to the daycare center in the laboratory by means of PCR, initially pooled, in case of a positive pool retested individually).

The laboratory result for the mouthwash sample is available within 24 h after collection. Participants are not notified if the result is negative in the pool or in the case of individual retests. The 'Case module' comes into effect in the case of a positive result (see 4.3.1.7).

In the case of the antigen rapid test at home the result can be read by the parents/child care staff after 15-30 minutes. If SARS-CoV-2 is detected in the antigen rapid test, the daycare center child or child care staff should not initially visit the daycare center. To confirm the antigen rapid test result a PCR test for SARS-CoV-2 from a throat swab should be performed on the same day. Appointments to this purpose can be made on the same day at the testing center of the University Hospital (test section building D20). Parents are asked to also hand in the mouthwash sample taken in the morning at the same time there for additional testing. The 'Case module' comes into effect in the case of a positive result (see 4.3.1.7). In case of a discrepant test result (positive antigen rapid test but negative PCR), another control smear test for the affected person is offered in the testing section of the University Hospital. This is made on the basis of individual counseling and taking into account exposure, symptoms, and symptoms in contact persons.

The submission of the mouthwash sample and the result of the findings are recorded in the laboratory/study center. Once a week the performance of all sample collections, the read result of the antigen rapid test, the presence/absence at the daycare center, the time estimated by the parents for sample collection/labeling/packaging as well as any 'workdays lost' due to a positive antigen rapid test are queried from the parents/child care staff.

The examinations take place for a 12-week period.

4.3.1.2 Group 2: Mouthwash sample submission (for PCR) 2x/week

Of all asymptomatic daycare center children and daycare center child care staff participating in Group 2, a mouthwash sample is taken 2x/week at home (for children by their parents) and tested for SARS-CoV-2 (mouthwash sample after submission in the daycare center in the laboratory by means of PCR, initially pooled, in the case of a positive pool result, retesting is carried out individually).

The laboratory result for the mouthwash sample is available within 24 h after collection. Participants are not notified if the result is negative in the pool or in the case of individual retests. The 'Case module' comes into effect in the case of a positive result (see 4.3.1.7).

The submission of the mouthwash samples and the result of the findings are recorded in the laboratory/study center. Once a week the performance of all sample collections, the presence/absence at the daycare center, the time estimated by the parents for sample collection/labeling/packaging are queried from the parents/child care staff.

The examinations take place for a 12-week period.

4.3.1.3 Group 3: Antigen rapid testing at home 2x/week

Of all asymptomatic daycare center children and child care staff participating in Group 3, a nasal swab is taken 2x/week at home (for children by their parents) and tested for SARS-CoV-2 (antigen rapid test at home according to manufacturer's instructions).

In the case of the antigen rapid test at home the result can be read by the parents/child care staff after 15-30 minutes. If SARS-CoV-2 is detected in the antigen rapid test, the daycare center child or child care staff should not initially visit the daycare center.

For participants with a positive antigen rapid test, additional follow-up examinations are offered. To this purpose, the study center can be contacted and a same-day appointment for PCR testing via throat swab can be arranged at the test center of the University Hospital. This ensures that the PCR result is available before the next daycare center visit, as far s possible. There is no notification to the Department of Public Health of an antigen rapid test reported as positive without the confirmation of a PCR test.

After 24 h, the result of the additional PCR test performed is available and the 'Case module' comes into effect in the case of a positive result (see 4.3.1.7.). In case of a discrepant test result (positive antigen rapid test but negative PCR), another control smear test for the affected person is offered in the testing section of the University Hospital. This is made on the basis of individual counseling and taking into account exposure, symptoms, and symptoms in contact persons.

The performance of all sample collections, the read result of the antigen rapid test, the presence/absence in the daycare centers, the time estimated by the parents for the sample collection and, if applicable, 'workdays lost' due to a positive antigen rapid test are queried online 1x/week from the parents/child care staff.

The examinations take place for a 12-week period.

4.3.1.4 Offer of additional examinations for symptomatic participants

Symptomatic participants (daycare center children and child care staff of the facilities), who are excluded from daycare center attendance according to the regulations of the Bavarian Hygiene Framework for Daycare Centers, are not allowed to visit the daycare center in the case of *potential symptoms* for COVID-19 as defined in the Hygiene Framework. In addition, all parents and child care staff will have the symptoms of COVID-19 explained to them by means of appropriate informational materials on the study website and via posting at the daycare center.

Symptomatic participants are offered an appointment for a timely throat swab for SARS-CoV-2 at the COVID-19 examination center at the University Hospital of Wuerzburg, House D20. In the COVID-19 examination center, the onset of disease and symptoms are additionally queried in a standardized questionnaire and transmitted to the study center.

If this is not accepted by the study participants, they are requested to inform the study secretariat of the result of any SARS-CoV-2 test performed by a third party (e.g., general practitioner).

Case module 4.3.1.7 will be followed if SARS-CoV-2 is detected in symptomatic study participants (daycare center children and child care staff).

Supplemental screening services are only valid as part of the study for the period during which respiratory screening is performed in asymptomatic participants (approximately 12 weeks). Medical care of symptomatic individuals will not take place as part of the study. It will

rather be the responsibility of the appropriate resident physicians/pediatricians of the affected individuals.

4.3.1.5 Sampling and processing

Samplings

As part of the study, parents or child care staff will collect respiratory samples from asymptomatic daycare center children or themselves (asymptomatic child care staff) at home on the scheduled day of testing according to the informational materials, online training, and videos provided by the study site or, in the case of antigen rapid testing, additionally according to the manufacturer's instructions.

The mouthwash sample collection are usually submitted to the daycare center (except for Group 1 in case of a positive rapid test, in which case they are submitted together with a throat swab at the examination center at the University Hospital). CE-marked antigen rapid tests are provided for self-testing by means of nasal swab, according to special approval for non-professional use (see BfArM - Antigen-Tests for SARS-CoV-2).

Since respiratory sampling is done at home, droplet and aerosol formation such when collecting samples at the daycare center is avoided. Screening by mouthwash sampling can only be used in children ≥ 2 years of age who are able to actively provide saliva. If possible, sampling should be done before taking breakfast/brushing teeth.

Throat swabs are taken at the COVID-19 examination center (University Hospital Wuerzburg, D20).

Pooling of mouthwash samples with retests

The processing, examination and reporting of the mouthwash sample is carried out at the Institute for Hygiene and Microbiology and/or the Institute for Virology at the University of Wuerzburg. This ensures that the result is received and reporting of findings and notification of results for SARS-CoV-2 is all done within 24 h the of mouthwash collection. In the case of a positive result, the respective participant will be contacted directly by phone and the Department of Public Health will be informed in accordance with Section 7 Infektionsschutzgesetz [Infection Protection Act] (4.3.1.6). Also the daycare center will be informed without a name being given that a case has occurred in the daycare center.

The pooling of PCR samples is carried out with the aim of increasing test capacities and saving costs. In accordance with the position paper of the working group for laboratory capacity at the RKI, pooling is used in the context of Wue-KiTa-CoV 2.0 "in the context of screening and surveillance investigations (e.g., testing of asymptomatic employees in medical facilities or for occupational health examinations) and/or surveillance examinations (e.g., indicator populations) with an expected very low prevalence or regionally correspondingly low 7-day incidence." (https://www.rki.de/DE/Content/InfAZ/N/Neuartiges_Coronavirus/Laborkapazitaeten.pdf?__blob=publicationFile).

The pooling strategy will be reviewed during the duration of the study depending on the local incidence and local age group-specific incidence of SARS-CoV-2. It will be adapted as necessary. The laboratory capacity working group considers a pooling of 5 individual samples possible. Much larger pools (up to 64) are described in the corresponding specialist

literature. From a pool size of about 15, relevant limitations of the sensitivity are to be expected (see https://ladr.de/sites/all/themes/cont/files/_01_bilder/01_medizin/02_beratung/infektiologie/200515_ALM-POSITIONSPAPIER_SARS-CoV-2-POOLING.pdf).
Within the framework of Wue-KiTa-CoV 2.0, pools of size 4-16 are to be used.
The respective pool size is documented.
Samples from daycare center children as well as samples from daycare center child care staff are first analyzed by pooling. It is NOT mandatory that the samples in the pool belong to the same daycare center or daycare center group. This is because the pool is dissolved in the event of a positive result and all participants in the positive pool are retested individually using the reserve samples.
The reserve samples are prepared in the laboratory. A minimum of 100 µl is needed to set up the pool, and 100-200 µl is needed for the reserve sample (based on previous experience from the previous study, mouthwash containers are reliably filled with 4-8 ml of mouthwash).

See Section 6.3.1 on the laboratory details.

Sample storage, additional tests

Random samples of the reserve samples from 3 specified time points will be stored until the end of the study. Otherwise the samples will be stored for approximately 1 week and then disposed of as directed.

In the declaration of consent form, it is pointed out that respiratory residual material can be stored and, if necessary, retested for scientific purposes at a later time by means of multiplex PCR for the presence of other common viral pathogens of respiratory infections (e.g. influenza virus, RSV, rhinovirus, other coronaviruses, adenovirus). Since these follow-up tests will not take place until several months after completion of the tests in the daycare centers, no further feedback of the results to the test persons or their parents/custodians is planned.

4.3.1.6 Communication of the proof of SARS-COV-2

If SARS-CoV-2 is detected by the laboratory (Virology/Microbiology) in symptomatic and asymptomatic participating children or child care staff, the laboratory will submit its findings to the Department of Public Health in accordance with Section 7 of the Infection Protection Act.

In addition, in the case of SARS-CoV-2 positive findings, the directly affected study participants will be informed by a physician from the study center and will receive a standardized information letter.

There is no information of the name by the laboratory/study center to the daycare centers. The daycare center is informed though that a positive result has been obtained from the daycare center, which has been forwarded to the Department of Public Health, and that - if this has not yet been done by the Department of Public Health- the daycare center should actively contact the Department of Public Health in order to prevent delays in outbreak management. Further informing of the daycare centers is effected place via the Department of Public Health.

4.3.1.7 Case module

In case of detection of SARS-CoV-2 in children or child care staff participating in the study in individual tests or in individual retesting of reserve samples, the laboratory will report to the

Wuerzburg Department of Public Health in accordance with Section 7 of the Infection Protection Act. Individuals who test positive are subject to the instructions of the Department of Public Health.

The decision as to whether the care facility of the child/child care staff who tested positive will remain open is the responsibility of the Wuerzburg Department of Public Health.

If the daycare center group should remain open for the time being in special agreement with the Wuerzburg Department of Public Health, there is the option of increasing the testing frequency of the study participants in the affected daycare center group and offering additional testing to non-study participants in the daycare center group as part of the study (see: Page 25/26).

Re-admission to the childcare facility is regulated in the Bavarian Hygiene Framework for Daycare Centers and is subject to the instructions of the Department of Public Health.

For control purposes, the Department of Public Health informs the study center about the total number of positively tested daycare center children/child care staff per daycare center and week (aggregated data only, no individual data). In addition, the Department of Public Health also provides the study center with the corresponding data from those daycare centers in Wuerzburg not participating to enable a descriptive comparison with the participating daycare centers.

4.3.2 Psychosocial dimension (questionnaire)

The successful implementation of the planned screening measures largely depends on acceptance by the parents, children, and institutions. So far there is little systematic data on the extent of willingness to participate as well as the acceptance of such a preventive measure related to the SARS-CoV-2 pandemic. Testing the feasibility of different screening records must therefore be accompanied by quantitative research and qualitative surveys of all participants. In addition, the pilot study offers the opportunity to assess factors influencing the acceptance of longer-term monitoring. This is in order to identify potential targets for accompanying interventions to increase acceptance.

Wue-KiTa-CoV 2.0 will collect questionnaire-based data at two points in time (at the beginning and after implementation of the measures) on the acceptance of the respective surveillance protocol from parents/custodians, children in daycare centers, and daycare center child care staff. Potential predictors of longer-term acceptance among participants will be examined:

a) Sociodemographic factors (such as education status, household size)

b) Expectations/attitudes of parents/custodians and child care staff regarding the pandemic

c) Expression of psychological symptoms (especially anxiety and depression). Questionnaires with validated psychometric instruments/scores (such as PHQ4, EQ-5D, and the anxiety/depression subscale of the CBCL 1½ -5 [23-25]) will be used, as well as a sociodemographic battery already in use in cooperation with the Robert Koch Institute as part of the CORONA HEALTH APP study (PI: Rüdiger Pryss, Co-PI: M Romanos).

Furthermore, qualitative interviews will be conducted with a subsample of parents/custodians as well as with child care staff from all facilities. This is to explore in greater depth the findings about expectations, desires, and reservations of the study participants regarding the

measures to be introduced. At the end of the intervention, follow-up interviews will explore reasons for acceptance or lack of acceptance in order to identify and, if possible, minimize potential barriers and obstacles of an implementation. The same interview partners will also be invited to the second interviews. Only in case of their non-participation are new participants recruited. The invitation to qualitative interviews takes into account as many different socio-demographic characteristics as possible when selecting interview partners. This is in order to achieve maximum contrast in terms of age, gender, education, marital status and place of residence (purposive sampling). This procedure is based on the assumption that the sociodemographic characteristics described above will have an influence on the aspects/topics to be identified in the interview material.

A semi-structured interview guideline is used for the interviews. The interviews are conducted by phone. The duration is estimated at approximately 30 minutes. The conversations are tape-recorded and then pseudonymized and transcribed. The evaluation is carried out content-analytically in a mixed deductive-inductive approach according to Kuckartz [26] using the MAXQDA software. The data collected during the interviews will be analyzed in parallel with the interviews. Additional study participants will be included until no new aspects/topics are to be expected anymore (data saturation) [27,28].

Children 3 years of age and older will be asked a question about the experience of respiratory sampling(s) by parents at two time points. Additionally parents are asked for their assessment of the child's readiness to participate.

4.3.3 Serology

For serological testing for the presence of antibodies against SARS-CoV-2, a few drops of blood will be taken from a prick of the fingertip of the subjects (children in care, as well as child care staff) at the beginning and at the end of the study. Blood collection is performed by trained personnel and under medical supervision.

Only in the case of positive or borderline findings, may a venous blood sample be taken for confirmation if consent is given (see 4.2).

Serological testing is performed by point-of-care testing on site and at the Institute of Hygiene and Microbiology and/or the Institute of Virology at the University of Wuerzburg.

If sample material is still available after testing, it is preserved for 10 years. Antibody tests for the detection of antibodies against SARS-CoV-2 are currently being further developed under high innovation pressure. The samples can thus be re-examined using future, improved procedures.

All study participants (or parents/custodians in the case of children) will be notified of the results of the serological testing.

For details on the test system: see Sections 6.3 and 6.4.

5 TEST PERSONS SAFETY, WORK SAFETY

Additional (optional) throat swabs for symptomatic daycare center children and daycare center child care staff, as well as blood sample collections, are always performed by medically trained personnel who can initiate appropriate emergency measures in the very rare event of complications.

The mouthwash collection and nasal swabs for the antigen rapid test, which can be performed by the parents after having received information by the study staff, should generally be performed on the collection days specified for each daycare center before the daycare visit (mouthwash collection before breakfast/before brushing teeth; in Group 1, parallel antigen rapid testing). Parents will have the testing explained to them prior to the first use in online information sessions using training videos that will also be made available for retrieval from the study website. A study hotline will be available for questions about sampling. A written instruction for sample collection including emergency number is handed out to the parents.

Transport of respiratory sample material

Antigen rapid tests:

Only approved antigen rapid tests for nasal swabs are used, in accordance with Special approval for non-professional use (see BfArM - Antigen tests for SARS-CoV-2). The performance and documentation of the results takes place at home, there is no transport of the used antigen rapid test. Parents dispose of the used antigen rapid tests according to the manufacturer's instructions.

Mouthwash tubes:

Information material and instructions on the correct collection and packaging are provided for parents on the website and in printed form. There is also an explicit reference made to the fact that tubes that may be wet on the outside should be dried before being placed in the prepared outer packaging. If applicable, mouthwash samples that have not been closed correctly are already secured against leakage by the outer packaging at home.

The sample tubes placed in the outer packaging are submitted by the parents together with the outer packaging at the daycare center in a suitable collection container. Hand disinfection materials are routinely available in the daycare center.

Collection of the collection container at the end of the daycare center admission time is arranged by the study center. The transport of the sample tubes takes place in the closed transport boxes with secondary packaging inside (transport regulation UN3373). Unpacking only takes place in the laboratory under safety precautions.

The German regulations and directives BioStoffV and TRBA also apply for transport and analytics.

6 DATA PROCESSING

6.1 Source data and material

Study data are obtained from participant lists, paper-based and electronic questionnaires, and from sample material.

Recruitment, registration

The study center will provide participating facilities with the study information material, test subject information and consent forms. The facilities will also receive pre-addressed sealable envelopes to the study center for parents/custodians or daycare center child care staff so they can submit the documents.

The participating facilities collect the envelopes with the declarations of consent from the parents and pass the collected documents on to the study staff. These documents are reviewed and archived at the study center, and daycare center-specific participant lists are created. Each participant is assigned a study number with the consent form (pseudonymization).

The study-specific online survey is stored pseudonymously via the EDC (Electronic Data Capture) system REDCap®.

The EDC system is hosted on servers at the University of Wuerzburg under the responsibility of the University of Wuerzburg Computer Center. The 'Security Policy' conforms to the standard of the University of Wuerzburg Access to the stored data is guaranteed via role and rights management of the EDC system.

Interview at the start of the study (Week 1) and end of the study (Week 12)

Participants provide their email address when consenting to the study. At two pre-defined time points (Week 1 and Week 12), they are interviewed using the REDCap® EDC system. To this purpose, the participants are sent access data (pseudonym and password) by e-mail. These can be used to verify themselves in the EDC system and then complete the surveys using eCRF.

Sample collection / tests

Pre-labeled materials will be provided to the parents in the case of mouthwash samples and antigen rapid tests taken out by the parents. The sample tubes with the mouthwash samples are submitted by the parents to the daycare center in transport materials which were provided to them. From there, collection and transport to the laboratory is carried out by the study center.

The time of sample collection and the results of the antigen rapid tests are queried online 1x/week via REDCap® for each participant and registered in the study database and are used for the documentation of the tests performed.

Blood samples for seroprevalence testing are collected, labeled, and documented by medically trained personnel. The samples are forwarded to the appropriate analysis sites at the Institutes of Microbiology or Virology at the University of Wuerzburg.

Laboratory samples are identified by name because feedback on conspicuous results should be provided to parents/daycare center child care staff and must be provided to the Department of Public Health if SARS-CoV-2 is detected.

In addition, in the case of missing mouthwash samples or missing information on the antigen rapid test, the presence of the participating children in the daycare centers (present / planned absent (e.g. vacation) / unexpected absent (e.g. due to illness) and the successful collection of the sample (nasal swab or mouthwash sample) are queried.

Children aged 3 years and older will be interviewed by parents at the Week 1 and Week 12 time points. This will be done using a simple online questionnaire ('smileys') about sampling. Parents will also be asked for their assessment of children's acceptance of the measures. The data are recorded in REDCap®.

Data on the acceptance of the measures by child care staff and parents will be collected quantitatively in questionnaires on all participants and qualitatively in semi-structured guided interviews in all groups of participants (after separate consent). These interviews are conducted by the staff of the Institute of General Medicine in collaboration with the Department of Child and Adolescent Psychiatry.

Basic data daycare centers

In addition, basic data on the daycare centers already available from Wue-KiTa-CoV will be updated by means of a daycare center questionnaire (including the total number of cases per daycare center children + child care staff, group sizes, number of child care staff/group) and sent to the study center and forwarded to the Institute for Clinical Epidemiology and Biometry for evaluation.

Information on days absent per daycare center and week is anonymously documented by the daycare center staff for the individual care units. The figures are sent to the study center and forwarded to the Institute for Clinical Epidemiology and Biometry for evaluation.

The number of mouthwash samples collected and antigen tests performed will be recorded at the study center based on the mouthwash samples provided and the rapid tests documented by parents/child care staff (to determine material costs, testing costs). Parents of participating daycare center children are asked to estimate their additional time required for sample collection/labeling/packaging.

6.2 Study database

The records on the subjects are managed centrally in the study secretariat. Only pseudonymized data will be collected (study center at the Institute of Hygiene and Microbiology, electronic questionnaires at the Institute of Epidemiology and Biometry).

The analyses of the sample material are performed at the University of Wuerzburg in the Institutes of Microbiology and Virology, respectively, and the results are recorded in Excel databases. The laboratory results are transmitted to the study center via a specially set up, protected exchange drive and forwarded in pseudonymized form to the Institute for Clinical Epidemiology and Biometry for evaluation.

The data from the questionnaires and the laboratory results will be merged pseudonymously for evaluation using the study number by the Institute of Clinical Epidemiology and Biometry.

The data required for the collection and evaluation are saved in two separate systems. Each participant is assigned a pseudonym so that on the surveys only the pseudonym and no personally identifying data is used. Through an assignment list and using a pseudonym the personally identifying data can be assigned to the research data. During the period of this assignment, the research data are considered "personal data" and data protection laws must be observed accordingly. To this purpose, a database is specially programmed by the central data processing unit (IKE-B). It will store the contact data and e-mail addresses and the associated pseudonym.

6.3 Laboratory methods

6.3.1 Virus identification

a) Polymerase chain reaction (PCR)

Pools with equal volumes per sample (approx. 100 µl), homogenized, RNA extraction (most likely Microgen RNA kit), PCR.

Pools are tested by RT-qPCR capacity-dependent among others with the following devices with CE marking: RotorGene (Qiagen), BDmax (BectonDickinson), NeuMoDX (Qiagen)

Single samples in the case of pool splitting are split by GenomEra CDX PCR ("rapid PCR").

The results of the PCR of the pools - after sample transport, pooling and PCR - will not be available until the afternoon. A technique with a very short time-to-result is therefore needed to split the pool as well as to communicate the positive finding to the test subject and the Department of Public Health. Using GenomaEra this is very short at 45 minutes (up to 8 samples). This means that using this equipment to split the pools allows the finding to be established and communicated while doing justice to the time requirements.

Pooling results including retesting from reserve samples are available within 24 hours.

An internal validation of the system will be performed prior to study initiation using 'spiked' samples of mouthwash water. If necessary, the sample quality is checked at two time points. This is done by detecting the number of copies of the albumin gene.

VOC testing:

All positive samples (mouthwash and throat swabs) will be analyzed for an N501Y mutation by melting curve analysis. Subsequently, PCR examines the sample for the Δ69-70 deletion (variant B.1.1.7). For N501Y mutations without Δ69-70 deletion, whole genome sequencing is performed to detect other variants.

b) CE-marked antigen rapid tests for self-testing (“for non-professionals”)

Antigen testing is performed by a yet to be determined test that is approved for non-professional testing i) according to BfArM and ii) approved for testing from a swab of the anterior nasal wall.

6.3.2 Antibody detection

Enzyme-linked immunosorbent Assay (ELISA), Point-of-Care –Test (lateral-flow ELISA)

Screening in daycare center with rapid test after finger tip punctuation: Abbott Panbio COVID-19 IgG/IgM (Target: Nucleocapsid, for test selection we refer to our own preliminary work: https://www.medrxiv.org/content/10.1101/2021.02.07.21251062v1), verification: ELISA Virion/Serion agil IgG (target spike protein) und Roche Elecsys Anti-SARS-CoV-2 IgG (target nucleocapsid). Through the screening vaccination responses should not be detected. All daycare center child care staff who are positive during screening will be asked their vaccination status to interpret the result. Thanks to the used serological tests with different targets, discrimination between an infection and a vaccination response is possible. All participants who screen positive will be asked about any previous PCR-confirmed Covid infections.

6.4 Samples

6.4.1 Nasal swabs for antigen rapid tests

Details on the implementation of sample collection:

Nasal swabs are taken as nasal concha swabs according to the manufacturer's instructions for each test. As a rule, the child's head is tilted back slightly, the swab is inserted into one nostril from the front and rotated 3 times. This sampling will be done by parents at home according to the manufacturer's instructions and standardized to the extent possible (via training of parents through printed informational materials, online training, and retrievable videos on the study website). There will also be specific instructions in addition to the manufacturer's instructions for sampling in children

There is no reuse of used materials.

6.4.2 Mouthwash samples

Details on the implementation of sample collection:

This sampling is done by parents at home and is standardized as much as possible (via training of parents through printed informational materials, online training, and retrievable videos on the study website).

Parents are given prepared labels and sample tubes (50 ml) for saliva samples. To collect the sample, a tablespoon of tap water or still water is taken into the mouth, rinsed, and the mouthwash is returned to the sample tube. The sample tube is labeled and packed in a sample bag and placed in a suitable collection container in the daycare center until the end of the daycare center admission time.

There is no reuse of used materials.

6.4.3 Blood samples

Details on the implementation of sample collection:

Blood samples are taken as fingertip pricks by using a safety lancet (disposable material). After disinfection and prick, the blood is taken up into a capillary from which it is applied to the point-of-care test.

In the case of abnormal or borderline findings (approximately 1 % of findings), a venous blood sample (1 ml, according to WHO- guideline [29]) is additionally offered. It is taken by a physician under specialist supervision after written consent.

7 BIOMETRIC ASPECTS

7.1 Endpoints

Primary endpoint:

Usability:

- Initial acceptance rate of the respiratory sampling (Group 1, 2, 3 combined) on the basis of the specifications on the declaration of consent (participant rate of all suitable participants)

Secondary endpoints:

Usability:

- Participation rate stratified by participant group (Group 1, 2, 3)
- Acceptance rate of the respective participant records defined as the share of 'successful' participations in all 'planned' specimen collections (mouthwash and/or nasal swab for non-professionals) among participating asymptomatic children and child care staff of the facility. For an individual participant, participation in specimen collection is considered successful when 60 % of the planned (asymptomatic) specimen collections have been successfully completed.

Effectivity (as a screening instrument):

- Detection rate of SARS-CoV-2 infections in the daycare centers in total per participant/per testing (daycare center children plus daycare center child care staff) and in relation to the 7-day incidence in the total population of children in the corresponding age group.

Further endpoints:

Usability:

- Acceptance of mouthwash submission (proportion of successful tests in Group 1 plus Group 2)
- Acceptance of the non-professional antigen rapid tests (nasal swab) (share of the tests carried out successfully in Group 1 plus Group 3)
- Predicators of the acceptance for longer-term usage
- Psychosocial effects, including satisfaction and fulfillment of parents' need for safety (using questionnaires and validated scores) at the beginning and end of the study
- In subgroups: Long-term psychosocial effects, including satisfaction and fulfillment of parents' need for safety (using questionnaires and validated scores) including participants of both Wue-KiTa-CoV and Wue-KiTa-CoV 2.0 (6-month evaluation)
- Satisfaction of the children (assessment by the parents and child questionnaire in Week 1 and Week 12).
- Frequency of consequences in the case of positive tests (daycare center group closures / daycare center closures, total 'workdays lost' due to PCR-confirmed detections, and additional ‘workdays lost' due to false-positive antigen rapid tests)

Effectivity (as a screening instrument):

- Detection rate of SARS-CoV-2 infections in daycare centers per participant group (Group 1, 2, 3), separately for children and child care staff
- Proportion of all positive PCR tests in total (mouthwash) (Group 1 + Group 2)
- Proportion of all positive antigen rapid tests in total (nasal swab) (Group 1 + Group 3)
- Comparison of detection rates of SARS-CoV-2 infections by means of non-professional antigen rapid testing (nasal swab) versus PCR (from mouthwash) in parallel testing of asymptomatic participants (Group 1) for identification of false-negative detections in the antigen rapid test.
- Identification of false-positive antigen rapid testing through retesting by means of PCR
- Length of time between sample collection and notification of a positive test result for PCR testing.
- Detection rates of SARS-CoV-2 Variants of Concern (VOCs), in total and over time
- Detection rates as a function of the ‘daycare center’ group composition (proportion of study participants, proportion of Group 1 participants)
- Proportion of positive serologic tests overall (children/child care staff), correlation of positive PCR and seroconversion between first and second serologic testing. This is a control for effectivity of surveillance approaches.

Efficiency (evaluation of economic efficiency):

- Costs for material and tests of asymptomatic participants (direct costs)
- Quantification of the influence of pooling on the test costs
- Costs of retesting positive antigen rapid tests of asymptomatic participants with PCR
- Transport costs (sample collection for laboratory)
- Parents' estimate of the average time spent sampling

Scalability (evaluation of further usability):

- Extrapolation of the number of participants, test consequences, and costs for all daycare centers in Wuerzburg
- Cost-benefit assessment

  Modeling of scalability as a function of participant rate, background incidence, test frequency, detection rate detection method, etc., using state-based modeling techniques.

Exploratory analyses regarding potential predictors of longer-term acceptance

- Longer-term acceptance with respect to respiratory sampling as a function of
  - Child’s age and sex
  - Family size
  - Education status

- Screening concept
- Personal assessment of the danger of SARS-CoV-2 infections
- Personal attitude towards SARS-CoV-2 vaccination

Exploratory analyses regarding psychosocial effects: Data collection with survey, based on the content of the Corona app of the RKI and qualitative interviews at the beginning and at the end of the intervention

- Parents' satisfaction with screening measure (Week 1, 12)
- Parents' sense of safety with screening measure (Week 1, 12)
- Subjective stress level through screening measure (Week 1, 12)
- Family climate worsened through screening measure (Week 1, 12)
- Everyday organization impaired through screening measure (Week 1, 12)
- Personal consequences assessed as negative occurred due to screening measure results (e.g., quarantine due to SARS-CoV-2 positive test) (Weeks 1, 12)
- Psychological symptoms using validated questionnaires (Week 1, 12)

Qualitative interviews: in-depth recording of expectations and attitudes of study participants before and after implementation of the screening measures.

- Before the intervention: Expectations, wishes, and reservations of study participants regarding screening measures
- After the intervention: Experiences with screening measures, reasons for acceptance or lack of acceptance, potential barriers and obstacles to implementation.

Child surveys on the acceptance of the measure (child questionnaire)

- Acceptance of the children (based on a question asked by parents - answer by means of 'smiley' questionnaire; and according to parent assessment (Week 1, 12).

Exploratory analyses regarding costs of the measure

- Average cost per sampling and index child, per week
- Average estimated parent/child care staff time per week for sampling/labeling/packaging (Week 1, 12).
- Average cost per detection of SARS-CoV-2 infection (number and cost of tests performed to detect SARS-CoV-2 infection).

Supplemental analyses of child care staff immunization status

SARS-CoV-2 vaccination status among daycare center child care staff (Week 1, 12).

7.2 Procedure for data analysis

To estimate the primary endpoints, the 95 % confidence interval is estimated using the Wilson score method. The significance level for all analyses is set to 5 %. Since these are purely exploratory analyses, the significance level is not adjusted for multiple testing. The data are analyzed stratified by daycare center children/parents and child care staff. Initially all

end points are described descriptively (frequency (percentage), mean value (SD) or median (IQR)). The development of psychosocial factors is evaluated overall and, if the number of cases is large enough, stratified by the participant group. This is done using tests for connected samples such as McNemar, ANOVA, and Friedmann. Potential predictors on the longer-term acceptance rate (drop-out rate) will be examined overall and stratified by the participant group. First in a descriptive manner using suitable tests such as Chi² test and later, if the number of cases is large enough, using logistic regression (adjusted for the participant groups). The analyses are effected by means of SAS, R or SPSS.

7.3 Analysis populations

For the main analysis, the data of all subjects are included in the analysis.

7.4 Evaluation points

Overview data on the study status and the tests performed for status reports are extracted from the study database on a weekly (short reports) or monthly basis. The main analysis of the study data will not begin until respiratory sampling is completed for all study sites (expected in mid/late July). An interim evaluation is scheduled for a project report in July (July/August). Full evaluation/final report will be completed by the end of 2021.

7.5 Case number discussion and power analysis

The primary endpoint is the initial acceptance rate (overall for Group 1, 2 and 3).

Eligible‘: 9 daycare centers in Wuerzburg, with approx. 650 potentially suitable children from 2 years of age and approx. 180 child care staff, organized in approx. 50 daycare center groups

Estimation of the initial number of participants based on the surveillance participation rate for mouthwash sampling (2x/week) determined in Wue-KiTa-CoV:

Children: 67 % of approx. 650 'eligible' children = approx. 436 children

A participation rate of 67 % can be estimated from 650 children with a precision of 3.7 % (half-width of the Wilson-score 95 % CI).

Child care staff: 86 % of approx. 180 'eligible' child care staff = approx. 155 child care staff

A participation rate of 86 % can be estimated from 180 child care staff with a precision of 5.3 % (half-width of the Wilson-score 95 % CI).

The maximum number of samples to be expected for a screening period of 12 weeks (participation of all potentially eligible 830 individuals in Participant group 1):

Approx. 20,000 mouthwash samples and approx. 20,000 documented antigen rapid tests.

Case number estimation for the qualitative interviews

For the interviews, we expect approximately 5 parent/child care staff participants per study group (Group 1, 2, or 3) to be recruited from all participating childcare centers. Overall we expect to conduct 30 interviews with parents/guardians (interviews at the beginning and end of the measures combined). Candidates from all facilities will be recruited for the child care staff interviews. Here we expect approximately 5 for the first and 5 for the second final interview (overall 10). The overall number of interviews is thus approx. 40.

8 QUALITY ASSURANCE AND CONTROL

The participant data will be collected in accordance with study-specific 'Standard Operating Procedures' in compliance with all legal data protection requirements, and only by authorized study personnel specifically trained for the study.

Quality assurance and control

Data collection and acquisition:

For quality assurance of data collection from questionnaires, paper-based data are either acquired twice and compared, or acquired once with post-testing of a sample (10 % of the data). This is done according to a pre-determined procedure. Electronic plausibility checks are performed on the raw data before electronic processing. Core data of electronically recorded questionnaires are already checked for completeness and plausibility during input by means of programmed plausibility checks. Electronic reminders are provided if data is not received.

Respiratory samples:

Parents will receive training material (printed and downloadable from the study website) and demonstrations (online training) on how to properly perform and read antigen rapid tests. The aim is to reduce or avoid false-positive antigen rapid test reports. An email contact address and study hotline will be provided for inquiries.

In the case of missing information on the antigen rapid test, a clarification with the parents/child care staff will be made via the study center (planned or unplanned absence from the daycare center). In case of a positive antigen rapid test, all participants will be offered an additional PCR test (quality assurance to exclude false-positive antigen rapid tests).

In case of absence of missing mouthwash samples, a clarification with the parents/child care staff will be made via the study center (planned or unplanned absence from the daycare center). If the volume of mouthwash samples is repeatedly too high or too low, participants will be contacted by telephone to prevent future sampling errors.

The collection, processing and reporting of laboratory samples is subject to the relevant regulations of the institutions involved.

Evaluation and publication:

Statistical analysis is performed according to a pre-determined analysis plan by university/university hospital staff who are qualified to do so.

Study results will be submitted for publication to a medical journal with external peer review procedure.

Data protection

All study data will be kept strictly confidential and will only be accessible to those directly involved in the study at the University or the University Hospital Wuerzburg. Data protection also has to be ensured at the participating external facilities through relevant regulations.

For data protection reasons, only pseudonymized data (by assigning a study number to each participant) will be electronically recorded and processed (with the exception of laboratory samples until the findings have been concluded). No data are collected in questionnaires and in the recording of semi-structured interviews that could directly identify a participant (such as name, date of birth, address).

For the merging of the laboratory results and the questionnaire data in pseudonymized form, a password-protected exchange drive will be set up on the (firewall-protected) server of the university/university hospital. Only those persons directly responsible for the study will have access to the server.

The data required for the collection and evaluation are saved in two separate systems. Each participant is assigned a pseudonym so that on the surveys only the pseudonym and no personally identifying data is used. Through an assignment list and using a pseudonym the personally identifying data can be assigned to the research data. During the period of this assignment, the research data are considered "personal data" and data protection laws must be observed accordingly. To this purpose, a database is specially programmed by the central data processing unit (IKE-B). It will store the contact data and e-mail addresses and the associated pseudonym. This database is stored in an access-restricted folder on a network drive of the Medical Informatics Service Center (SMI) of Wuerzburg University Hospital (UKW). The data saved here will at no point be merged with the research data. All personal data in this database are accessible only to those project staff members responsible for sending the e-mails in the project. These staff members are either bound to medical confidentiality and/or are obliged to maintain data secrecy in accordance with the EU -GDPR as well as regional laws. They will not be passed on to third parties under any circumstances. All personal data will be deleted after completion of the study.

8.1 Data flow diagram / responsibilities

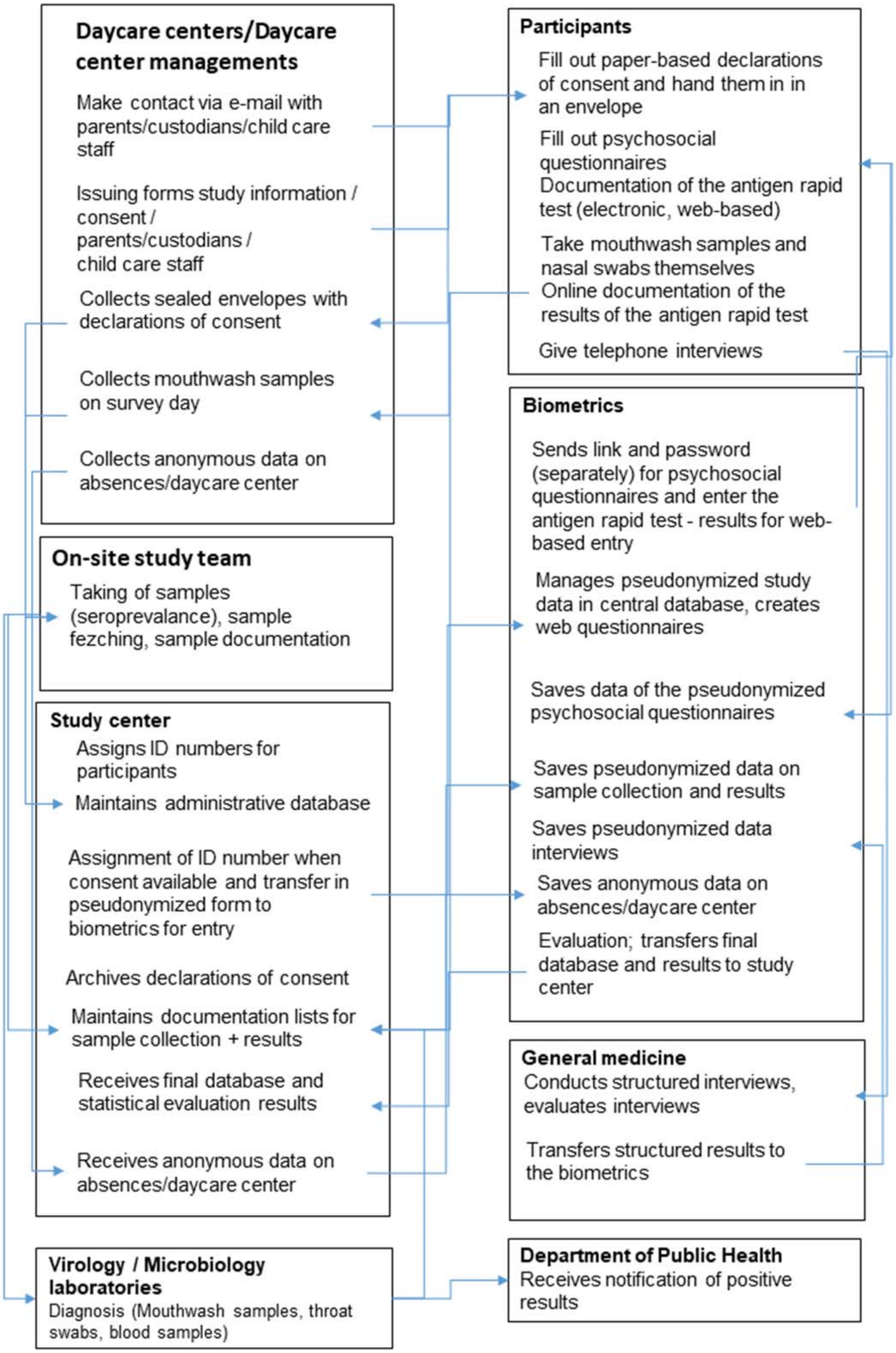

9 PUBLICATION OF THE RESULTS

Timely publication of the results complete completion of the study in a medical journal is aimed for.

The LGL receives status and project reports:

- Status report April 2021 ('Manual')
- Status report May 2021
- Status report June 2021
- Project report July/August 2021 (”meaningful”)
- Final report Sep 2021 (provisional; main evaluation)
- Final report Dec 2021 (final evaluation)

Plus there are weekly short reports in regards to:

- N included children (only during recruitment phase)
- N documented tests per week
- Cumulative since project start:

a) Positive tests (pool/individual tests mouthwash, documented antigen rapid tests)

b) If appropriate, correct-positive (after PCR confirmation of antigen rapid tests)

c) if appropriate, wrong-negative tests after PCR confirmation (only possible for participant Group 1 tested in parallel)

10 TEST PERSONS INSURANCE

In terms of sampling, the antigen rapid non-professional tests and mouthwash sampling are done by parents. These are samples with very low risk profiles, accordingly there is no test subject insurance for these. Sampling by study staff (throat swabs, blood samples) are study-related, non-medically justified 'interventions' with a low risk profile. Before the start of sampling, a ‘test persons insurance for clinical trials not subject to compulsory insurance (according to AMG/MPG)' should be concluded for samples taken by study staff. There is no framework agreement of the legal department of the UKW with the HDI Gerling insurance company For the individual subject, the corresponding sum insured is expected to be a maximum of EUR 250,000.00. For all insured events in the study a maximum of EUR 5,000,000.00 (see General Conditions of Insurance for Clinical Trials Not Subject to Compulsory Insurance/Annual Contract, AVB-Prob/NV-JV, Form. 404 U205).

11 ETHICAL BASIS

11.1 Ethics vote

An ethics vote is obtained from the Ethics Committee at the Medical Faculty of the University of Wuerzburg. In the predecessor study Wue-KiTa-CoV, the principle procedure of mouthwash submission and all accompanying investigations (surveys, seroprevalence testing) was already reviewed under ethical aspects and approved.

11.2 Test persons information and consent for sample collection

The information on Wue-KiTa-CoV 2.0 will be provided in writing in the form of an information sheet on the study. This also covers voluntary participation, the possibility of withdrawing consent (at any time), and any risks. In addition, verbal clarification and information will be provided prior to sample collection (blood samples for seroprevalence testing only; all other sample collection will be done by parents; training for this will be provided through online information sessions and videos on the study website). For previous participants of Wue-KiTa-CoV, renewed clarification and consent for Wue-KiTa-CoV 2.0 takes place.

The written consent from both parents/custodians of participating daycare center children, or from daycare center child care staff on a consent form is required.

If required, additional forms will be provided in English and Arabic.

11.3 Consent to take part in the study

Except in the case of sole custody by one parent, the written consent of both parents/custodians of participating daycare center children or the written consent of daycare center child care staff on a consent form after verbal and written information is required.

The following explicit consents will be obtained in addition to consent for study participation including online surveys:

- For mouthwash sampling or antigen rapid testing or parallel testing of both procedures, with choice of participant group (Group 1, 2 or 3), including willingness for documentation.
- For fingertip pricks to check for SARS-CoV-2 antibodies
- In the case of former participants in the predecessor study Wue-KiTa-CoV, consent to the transfer of information/data from the predecessor study to Wue-KiTa-CoV 2.0

After selecting a sample, additional consents are obtained:

- On semi-structured interviews (sample of participants)

If required, additional forms will be provided in English and Arabic.

11.4 Usage, storage and transfer of the data

All data on test persons will be collected and used only for the purpose stated in the study protocol. Only authorized study personnel acquires and manages data in compliance with all valid data protection regulations. The procedure for compliance with data protection corresponds to the procedure for Wue-KiTa-CoV. This procedure has been approved by the data protection officer of the University of Wuerzburg.

Transport of study documents/samples from participating institutions to the study center will be performed only by authorized personnel according to predetermined SOPs.

In electronic form, only pseudonymized data are collected and stored. The storage/processing of data is carried out exclusively using the technical facilities of the University / University Hospital. This takes place on device that have appropriate password protection and on their premises, in order to ensure the security of the data.

Data will only be passed on / exchanged between named, directly involved study managers and offices at the University of Wuerzburg and the University Hospital of Wuerzburg (UKW) via a specially set up, password-protected exchange drive. No study data will be passed on to third parties.

Only aggregated data, which do not allow any conclusions to be drawn about the persons/households involved are published.

All study documents are kept and archived in accordance with legal requirements, and are destroyed after the prescribed period.

12 LITERATURE

1. Bertoncelli D, Guidarini M, Della Greca A, et al. COVID19: potential cardiovascular issues in pediatric patients. *Acta bio-medica: Atenei Parmensis.* 2020;91(2):177-183.
2. Robert Koch Institut. SARS-CoV-2 Steckbrief zur Coronavirus-Krankheit-2019 (COVID-19) [SARS-CoV-2 Coronavirus disease fact sheet 2019 (COVID-19)]. https://www.rki.de/DE/Content/InfAZ/N/Neuartiges_Coronavirus/Steckbrief.html#doc13776792bodyText2. Published 2020. Accessed 2020-07-22.
3. Streng A, Hartmann K, Armann J, Berner R, Liese JG. [COVID-19 in hospitalized children and adolescents]. *Monatsschr Kinderheilkd.* 2020:1-12.
4. World Health Organisation. Coronavirus disease pandemic (COVID-19). https://www.euro.who.int/de/health-topics/health-emergencies/coronavirus-covid-19/novel-coronavirus-2019-ncov. Published 2020. Accessed.
5. Deutsche Akademie für Kinder- und Jugendmedizin. [German Academy of Medicine for Children and Adolescents] Stellungnahme der Deutschen Akademie für Kinder- und Jugendmedizin e.V. zu weiteren Einschränkungen der Lebensbedingungen von Kindern und Jugendlichen in der Pandemie mit dem neuen Coronavirus (SARS-CoV-2). [Statement by the German Academy of Medicine for Children and Adolescents n further restrictions on the living conditions of children and adolescents in the pandemic with the new coronavirus (SARS-CoV-2)] https://www.dakj.de/stellungnahmen/stellungnahme-der-deutschen-akademie-fuer-kinder-und-jugendmedizin-e-v-zu-weiteren-einschraenkungen-der-lebensbedingungen-von-kindern-und-jugendlichen-in-der-pandemie-mit-dem-neuen-coronavirus-sar/. Published 2020. Accessed 2020-07-18.
6. Schober T, Rack-Hoch A, Kern A, von Both U, Hübner J. Coronakrise: Kinder haben das Recht auf Bildung. [Covid crisis: Children have the right to education.] *Dtsch Arztebl International.* 2020;117(19):990-994.
7. Panahi L, Amiri M, Pouy S. Clinical Characteristics of COVID-19 Infection in Newborns and Pediatrics: A Systematic Review. *Archives of academic emergency medicine.* 2020;8(1):e50.
8. Ludvigsson JF. Systematic review of COVID-19 in children shows milder cases and a better prognosis than adults. *Acta Paediatr.* 2020;109(6):1088-1095.
9. Alunno A, Carubbi F, Rodríguez-Carrio J. Storm, typhoon, cyclone or hurricane in patients with COVID-19? Beware of the same storm that has a different origin. *RMD open.* 2020;6(1).
10. Verdoni L, Mazza A, Gervasoni A, et al. An outbreak of severe Kawasaki-like disease at the Italian epicentre of the SARS-CoV-2 epidemic: an observational cohort study. *Lancet.* 2020;395(10239):1771-1778.
11. Garazzino S, Montagnani C, Donà D, et al. Multicentre Italian study of SARS-CoV-2 infection in children and adolescents, preliminary data as at 10 April 2020. *Euro surveillance : bulletin Europeen sur les maladies transmissibles = European communicable disease bulletin.* 2020;25(18).
12. Robert Koch Institut. COVID-19: Fallzahlen in Deutschland und weltweit. [Case numbers in Germany and worldwide.] https://www.rki.de/DE/Content/InfAZ/N/Neuartiges_Coronavirus/Fallzahlen.html. Published 2020. Accessed 2020-07-22.
13. Danis K, Epaulard O, Bénet T, et al. Cluster of coronavirus disease 2019 (Covid-19) in the French Alps, 2020. *Clin Infect Dis.* 2020.
14. National Centre for Immunisation Research and Surveillance (NCIRS). COVID-19 in schools – the experience in NSW. http:// ncirs. org. au/ sites/default/ files/ 2020- 04/ NCIRS% 20NSW% 20Schools%20COVID_ Summary_ FINAL% 20public_ 26% 20April%202020. pdf. Published 2020. Accessed.
15. Gudbjartsson DF, Helgason A, Jonsson H, et al. Spread of SARS-CoV-2 in the Icelandic Population. *New England Journal of Medicine.* 2020.
16. Lavezzo E, Franchin E, Ciavarella C, et al. Suppression of COVID-19 outbreak in the municipality of Vo, Italy. *medRxiv.* 2020.
17. Mizumoto K, Omori R, Nishiura H. Age specificity of cases and attack rate of novel coronavirus disease (COVID-19). *medRxiv.* 2020.

18. Viner RM, Russell SJ, Croker H, et al. School closure and management practices during coronavirus outbreaks including COVID-19: a rapid systematic review. *The Lancet Child & adolescent health.* 2020;4(5):397-404.
19. Bi Q, Wu Y, Mei S, et al. Epidemiology and transmission of COVID-19 in 391 cases and 1286 of their close contacts in Shenzhen, China: a retrospective cohort study. *The Lancet Infectious diseases.* 2020.
20. Fontanet A, Tondeur L, Madec Y, et al. Cluster of COVID-19 in northern France: A retrospective closed cohort study. *medRxiv.* 2020.
21. Terry C. Jones BM, Talitha Veith, Marta Zuchowski, Jörg Hofmann, Angela Stein, Anke Edelmann, Victor Max Corman, Christian Drosten. An analysis of SARS-CoV-2 viral load by patient age. https://zoonosen.charite.de/fileadmin/user_upload/microsites/m_cc05/virologie-ccm/dateien_upload/Weitere_Dateien/analysis-of-SARS-CoV-2-viral-load-by-patient-age.pdf. Published 2020. Accessed.
22. Brauner JM, Mindermann S, Sharma M, et al. The effectivity and perceived burden of nonpharmaceutical interventions against COVID-19 transmission: a modelling study with 41 countries. *medRxiv.* 2020.
23. Achenbach TM. *The Achenbach system of empirically based assessment (ASEBA): Development, findings, theory, and applications.* University of Vermont, Research Center for Children, Youth, & Families; 2009.
24. EuroQol. EuroQol--a new facility for the measurement of health-related quality of life. *Health policy (Amsterdam, Netherlands).* 1990;16(3):199-208.
25. Löwe B, Wahl I, Rose M, et al. A 4-item measure of depression and anxiety: validation and standardization of the Patient Health Questionnaire-4 (PHQ-4) in the general population. *Journal of affective disorders.* 2010;122(1-2):86-95.
26. Kuckartz U. *Qualitative Inhaltsanalyse: Methoden, Praxis, Computerunterstützung. [Qualitative content analysis: Methods, practice, computer support.]* Weinheim: Beltz-Juventa; 2012.
27. Moser A, Korstjens I. Series: Practical guidance to qualitative research. Part 3: Sampling, data collection and analysis. *European Journal of General Practice.* 2018;24(1):9-18.
28. Guest G, Bunce A, Johnson L. How Many Interviews Are Enough?: An Experiment with Data Saturation and Variability. *Field Methods.* 2006;18(1):59-82.
29. World Health Organization. *WHO guidelines on drawing blood: best practices in phlebotomy.* World Health Organization; 2010.

13 APPENDIX

13.1 Participant information

- *Information for parents/custodians*
- *Information for child care staff*

13.2 Declaration of consent

- *Consent for parents/custodians*
- *Consent for child care staff*

13.3 Survey tools

Survey on all participants

- *Initial survey of basal demographic data (consent, online interview Week 1)*
- *Psychosocial questionnaires (online interviews Week 1, Week 12)*
- *Children interviews (online interviews Week 1, Week 12)*
- *Recording of intended/performed sample collections (weekly online interviews)*

Other surveys (in preparation)

- *Structured interviews (on the basis of a guideline; survey only for random sample)*
- *Recording of cost factors (material, personnel and required time)*
- *Daycare center interview (including number of children, group size, SARS-CoV-2-related hygiene measures; weekly aggregated figures on absences of daycare center children and child care staff).*

13.4 Further information material

(in preparation)

- Postings in the daycare centers
- Information flyer for parents
- Power point presentation for information events
- Study website with videos (instruction for sample collection by parents)
