# Supplementary material for: Acceptance of Different Self-sampling Methods for Semiweekly SARS-CoV-2 Testing in Asymptomatic Children and Childcare Workers at German Day Care Centers: A Nonrandomized Controlled Trial
Source: JAMA Netw Open. 2022 Sep 15;5(9):e2231798. doi: 10.1001/jamanetworkopen.2022.31798 (PMC9478779; doi:10.1001/jamanetworkopen.2022.31798)
Supplement: Supplement 2. — eAppendix 1. Supplemental Methods eAppendix 2. Supplemental Results eTable 1. Sociodemographic Characteristics of Participating Children and Parents, Attitudes Toward the Pandemic/Experience of Testing/Perception of Health Burden of the Pandemic, Week 1 eTable 2. Sociodemographic Characteristics of Participating Childcare Workers, Attitudes Toward the Pandemic/Experience of Self-testing/Perception of Health Burden of the Pandemic, Week 1 eTable 3. Comparison of Children's Health Status/Readiness to Participate and Parents' Perceptions of Respiratory Surveillance Measures/Attitudes Toward the Pandemic at Weeks 1 and 12 eTable 4. Comparison of Perceptions of Respiratory Surveillance Measures and Attitudes Toward the Pandemic of Participating Childcare Workers at Weeks 1 and 12 eTable 5. Overall Number and Rates of Saliva Testing per Week and per Month Throughout the Study Period for Children and CCWs eTable 6. Factors Influencing Children's Unsuccessful Long-term Participation eTable 7. Factors Influencing Successful Participation of Childcare Workers eFigure 1. Flow Diagram eFigure 2. Age-Adjusted Incidences in the City and District of Wuerzburg for the DCC-Relevant Age Group (2-6 years; orange) and Corresponding Incidences Among Children in all 69 DCCs (blue) During the Study Period eReferences. [file jamanetwopen-e2231798-s002.pdf]

Supplemental Online Content

Engels G, Forster J, Streng A, et al; Wue-KiTa-CoV 2.0-Study Group. Acceptance of different self-sampling methods for semiweekly SARS-CoV-2 testing in asymptomatic children and childcare workers at German day care centers: a nonrandomized controlled trial. *JAMA Netw Open.* 2022;5(9):e2231798. doi:10.1001/jamanetworkopen.2022.31798

eAppendix 1. Supplemental Methods

eAppendix 2. Supplemental Results

eTable 1. Sociodemographic Characteristics of Participating Children and Parents, Attitudes Toward the Pandemic/Experience of Testing/Perception of Health Burden of the Pandemic, Week 1

eTable 2. Sociodemographic Characteristics of Participating Childcare Workers, Attitudes Toward the Pandemic/Experience of Self-testing/Perception of Health Burden of the Pandemic, Week 1

eTable 3. Comparison of Children's Health Status/Readiness to Participate and Parents' Perceptions of Respiratory Surveillance Measures/Attitudes Toward the Pandemic at Weeks 1 and 12

eTable 4. Comparison of Perceptions of Respiratory Surveillance Measures and Attitudes Toward the Pandemic of Participating Childcare Workers at Weeks 1 and 12

eTable 5. Factors Influencing Children's Unsuccessful Long-term Participation

eTable 6. Factors Influencing Successful Participation of Childcare Workers

eFigure 1. Flow Diagram

eFigure 2. Age-Adjusted Incidences in the City and District of Wuerzburg for the DCC-Relevant Age Group (2-6 Years; Orange) and Corresponding Incidences Among Children in all 69 DCCs (Blue) During the Study Period

eReferences.

© 2022 Engels G et al. *JAMA Network Open.*

eAppendix 1. Supplemental Methods

Material and testing procedures

SARS-CoV-2 PCR. Saliva specimens were transferred to the central laboratory and analysed in pools of a maximum of 8 samples (100µl of each specimen). A retention sample was stored. SARS-CoV-2 nucleic acid testing was performed using CE certified tests (Cobas 6800 [Roche], Alinity M [Abott], NeuMoDx [Qiagen], BDmax [Becton Dickinson]. In the event of a positive or unclear pool result, the retention sample was analysed. Positive results were reported on the day of sampling.

SARS-CoV-2 antigen rapid self-tests. AST (AESKU.RAPID SARS-CoV-2 Rapid Test, Wendelsheim, Germany) kits and instructions were distributed to the participants in group 1 and group 3. The AgRTs were carried out at home using nasal atrium swabs performed in the morning by parents on their children or self-administered by CCWs and documented by parents/CCWs using an online tool developed for the study. Participants were instructed to stay at home and contact the study site for additional PCR testing in the event of a positive test result.

SARS-CoV-2 antibody testing. Before and after the period of continuous testing, study participants were tested for SARS-CoV-2 IgG antibody status. For children who had participated in our previous surveillance study[1] and for whom serological testing results were available from the end of the prior study, those results were included in the present study and additional serological testing was offered only at week 12. A finger-prick test was performed at the DCCs by medical personnel and analysed by point-of-care testing (Panbio COVID-19 IgG/IgM Rapid Test, Abbott, North Chicago, USA). In the event of a positive point-of-care test result, full serology testing was offered to all participants using SERION ELISA agile SARS-CoV-2 (Institut Virion/Serion, Wuerzburg, Germany, target: SARS-CoV-2 spike protein) and Elecsys Anti-SARS-CoV-2 (Hoffmann-La Roche AG, Basel, Switzerland, target: SARS-CoV-2 nucleocapsid)[2]. All IgG positive samples from finger-prick or venous samples were considered indicative of a previous SARS-CoV-2 infection.

Questionnaires and interviews

The questionnaires used included the Children with Special Health Care Needs (CSHCN) - Screener[3], the subscale "anxious/depressed" of the German version of the Child Behaviour Checklist 1·5-5[4] and the Patient Health Questionnaire-4 (PHQ-4). For a more detailed description, we refer to Forster et al[1].

Statistical analysis

© 2022 Engels G et al. *JAMA Network Open.*

Univariable and multivariable logistic regression analysis

To account for data dependencies within families, univariable and multivariable generalized estimation equations (GEE) with random effects for families were used in the analyses to determine factors associated with unsuccessful participation of children. Two models were built. Model 1 included allocation to study group and those baseline characteristics that were significant at a significance level of 10% in univariable analyses as well as baseline characteristics that were considered as potential predictors from a contextual perspective. Model 2 combined baseline characteristics with the attitude towards SARS-CoV-2 (sum score of variables measuring "anxiety about COVID" [maximum sum score 12, cut off value >6]). Successful participation was pre-defined as at least 60% of all scheduled respiratory samples being taken (excluding sick days, holidays etc). Similar to the method used in the analyses of the children's data, multivariable logistic regression was used to model factors associated with unsuccessful participation of CCWs.

Sample size discussion

The maximum achievable sample size was limited by the number of children and CCWs in the pre-selected nine DCCs. We assumed a number of 650 eligible children and an initial participation rate of 67%, which could be estimated with a precision of 3.7% (half-width of the Score-Wilson 95%-CI). Assuming a number of 180 CCWs and an initial acceptance rate of 86%, the acceptance rate could be estimated with a precision of 5.3%.

Modelling

We adjusted incidences reported for the city and district of Wuerzburg to the DCC-relevant age group 2 – 6 years and compared these age-adjusted incidences with the incidences among children in all 69 DCCs during the study period. As can be seen in **eFigure 2**, the incidence rates for the city and district of Wuerzburg represent an upper limit to the incidences reported for children attending the DCCs, an observation which can be interpreted as the effect of restricting access of symptomatic children to DCCs. Thus, the incidence rates for the city and district of Wuerzburg can be interpreted as a worst-case-scenario for DCC incidences and can be used as a basis for deriving a reference point at which testing becomes reasonable. We assume that the probability of the introduction of a primary infection is homogenous across children and depends on the age-adjusted incidence. For modelling the probability

© 2022 Engels G et al. *JAMA Network Open.*

that at least one primary infection occurs, we use a disease occurrence model based on a binomial distribution[5]:

$$P(X > 0) = 1 - P(X = 0) = 1 - \left(1 - \frac{p}{7 \cdot 100{,}000}\right)^{N \cdot d}$$

where N is the cohort size of the DCC, p the age-adjusted 7-day incidence and d the duration in which the occurrence of infections is to be modelled. It should be noted that we are only interested in primary infections and do not apply this model to transmission effects inside a DCC. **Figure 3** illustrates how the age-adjusted incidence affects the probability that at least one positive child is introduced as a primary infection case in a DCC. For example, for a DCC size of 50, 75 or 100 children, if the age-adjusted incidence is below 143, 95 and 71, respectively, the probability that at least one primary infected child will enter the DCC during the week ($d = 5$) is at most 5%.

© 2022 Engels G et al. *JAMA Network Open.*

eAppendix 2. Supplemental Results

Sociodemographic characteristics of participants and initial attitude towards the pandemic

Children/parents

Full data on participating children and parents are provided in **eTable 1.**

Of the participating parents, 78.0% (252/323) had completed school at university entrance level. The most frequent household size was 4 persons (median; IQR 3-4). The majority of the responding parents were in employment, either part-time (46.1%; 147/319) or full-time (30.1%; 96/319); in 78.5% (237/302) of all participating households, all adults were in employment. Of all participating households, 19.5% (63/323) included at least one person with a chronic health condition**.**

Initially, 24% of the parents felt strongly affected by their fear of transmitting a potential SARS-CoV-2 infection to other persons and 16% feared that they might develop a severe course of COVID-19 in the event of an infection. Only 6% stated that they had critical/very critical views towards the planned DCC surveillance of their children; 12% declared that they feared negative consequences of the planned DCC surveillance for their family. Regarding their attitudes towards vaccinations in general, 94% of the parents considered vaccinations as fairly/very important.

There were only few significant differences between the three study groups. In group 1, the number of children per household was slightly larger (p=.016) than in group 2 or group 3, the parents' willingness to have their children vaccinated against SARS-CoV-2 was higher (66.5% vs. 45.7% and 50.0%; p=.005), a higher proportion considered testing of children as rather/very useful (group 1 90.9% vs. groups 2/3 78.9%/80.4%, p=.001) and their attitude towards the planned measures in the DCC was more often positive ("rather/very positive" group 1 88.2% vs. groups 2/3 65.7%/66.6%; p<.001).

CCWs

Full data on participating CCWs are provided in **eTable 2.**

Of responding CCWs, 54.5% (60/110 with available information) had completed school at university entrance level and 57.8% (63/109) lived in households with 1-2 persons. Approximately one fifth (21.8%; 24/110) of CCWs reported a chronic health condition for themselves and 18.3% (20/109) for another household member. Questionnaires for week 1 were available for 112 of 139 CCWs. Of these 112

© 2022 Engels G et al. *JAMA Network Open.*

CCWs, 57% felt strongly affected by the fear of transmitting a potential SARS-CoV-2 infection to others and 30% reported fear of developing a severe course of COVID-19 themselves. Government-mandated restrictive measures were classed as fairly/very important by 68% of participating CCWs. Only 10% stated fairly strong or strong fear of potential negative effects of the planned surveillance measures.

Change of attitude and vaccination status during the course of the study

Parents

From week 1 to week 12, the proportion of parents vaccinated against SARS-CoV-2 increased from 37% to 80% (p<.001). Correspondingly, the proportion of parents considering SARS-CoV-2 as dangerous for themselves and their families (p=.013) or for society (p<.001) decreased. Compared to week 1, significantly fewer parents felt limited in their personal lives (p<.001) or concerned regarding potential negative impacts of the testing measures on their families (p<.001). They were also less fearful regarding their own health, the risk of SARS-CoV-2 infection or severe disease and the risk of infecting others (all p=.005 or lower). In contrast, there was no change in parents' opinions regarding the need for restrictive measures by the state (p=.508) or the impact of the respiratory surveillance on their sense of safety regarding SARS-CoV-2 infections (p=.332).

CCWs

In week 12, 70.3% (45/64) of participating CCWs reported that they were vaccinated against SARS-CoV-2. Compared to week 1, at week 12 fewer CCWs considered SARS-CoV-2 a danger for themselves/their families (p=.001; week 1: 62.9% [39/62]; week 12: 37.1% [23/62]) or for society (p=.039; week 1: 79% [49/62]; week 12: 66.1% [41/62]) and fewer CCWs felt restricted in their personal lives due to the pandemic (p=.011; week 1: 66.1% [41/62]; week 12: 41.9% [26/62]). By week 12, a higher proportion of CCWs did not feel affected by worries of becoming infected with SARS-CoV-2 (p=0.010; week 1: 16.1% [10/62]; week 12: 27.4% [17/62]), of developing a severe course of COVID-19 disease (p=.041; week 1: 21.7% [13/60]; week 12: 30% [18/60]), of receiving inadequate treatment for the disease (p=.040; week 1: 35.5% [22/62]; week 12: 53.2% [33/62]) or of infecting other people (p<.001; week 1: 6.5% [4/62]; week 12: 17.7% [11/62]). Of all participating CCWs with available data, 19.2% (20/104) initially showed an elevated PHQ4 score for anxiety / depression; in 40% (4/10) of those

© 2022 Engels G et al. *JAMA Network Open.*

CCWs with initially elevated PHQ4 values and available data for week 12, the PHQ4 score had decreased to normal values (not significant; p=.289).

© 2022 Engels G et al. *JAMA Network Open.*

eTable 1. Sociodemographic Characteristics of Participating Children and Parents, Attitudes Toward the Pandemic/Experience of Testing/Perception of Health Burden of the Pandemic, Week 1

N=total, SAL=saliva (mouthwash) samples, AgRT=rapid antigen self-test, CSHCN=Children with Special Health Care Needs-Screener, CBCL=Child Behavior Checklist, PHQ-4=Patient Health Questionnaire 4.

Children/Parents	**All participants**		**Group 1 (SAL + AgRT)**		**Group 2 (SAL)**		**Group 3 (AgRT)**		
	**N**	**n (%) or Median IQR**	**N**	**n (%) or Median IQR**	**N**	**n (%) or Median IQR**	**N**	**n (%) or Median IQR**	**p-value**
**Socio-demographic data**									
Children									
Age, median (IQR)	452	4 (3-5)	215	4 (3-5)	172	4 (3-5)	65	3 (2-4)	<0.0001
Female, n(%)	452	213 (47.1)	215	94 (43.7)	172	88 (51.2)	65	31 (47.7)	0.3441
Chronic health conditions (any), n(%)	373	51 (13.7)	193	29 (15)	125	14 (11.2)	55	8 (14.5)	0.6119
German nationality, n(%)	364	353 (97)	187	184 (98.4)	123	116 (94.3)	54	53 (98.1)	0.1041
CSHCN above normal	369	26 (7)	190	16 (8.4)	124	4 (3.2)	55	6 (10.9)	0.1020
CBCL above normal	361	30 (8.3)	188	15 (8)	119	8 (6.7)	54	7 (13)	0.3763
Parents									
Age, median (IQR)	322	38 (34-40)	173	38 (36-41)	104	37 (34-40)	45	36 (32-39)	0.0500
Female, n(%)	323	237 (73.4)	173	125 (72.3)	104	77 (74)	46	35 (76.1)	0.8573
School education, n(%)	323		174		103		46		0.1865
None		1 (0.3)		0 (0.0)		1 (1.0)		0 (0.0)	
Lower secondary level / basic		20 (6.2)		10 (5.7)		6 (5.8)		4 (8.7)	
Lower secondary level/ advanced		50 (15.5)		21 (12.1)		17 (16.5)		12 (26.1)	
Upper secondary level (University entrance level)		252 (78)		143 (82.2)		79 (76.7)		30 (65.2)	
Employment status, n(%)	319		172		102		45		0.9617
Employed/ full-time		96 (30.1)		54 (31.4)		31 (30.4)		11 (24.4)	

© 2022 Engels G et al. *JAMA Network Open.*

Employed/ part-time		147 (46.1)		76 (44.2)		48 (47.1)		23 (51.1)	
Self-employed		14 (4.4)		8 (4.7)		5 (4.9)		1 (2.2)	
Unemployed		24 (7.5)		13 (7.6)		8 (7.8)		3 (6.7)	
Importance of childcare access in daily life, n(%)	325		174		105		46		0.1682
Not at all		2 (0.6)		2 (1.1)		0 (0.0)		0 (0.0)	
Somewhat		10 (3.1)		4 (2.3)		5 (4.8)		1 (2.2)	
Moderately		32 (9.8)		21 (12.1)		8 (7.6)		3 (6.5)	
Fairly		94 (28.9)		41 (23.6)		40 (38.1)		13 (28.3)	
Very		187 (57.5)		106 (60.9)		52 (49.5)		29 (63)	
Number of persons per household, median (IQR)	324	4 (3-4)	173	4 (4-4)	105	4 (3-4)	46	4 (3-4)	0.2415
Adults	321	2 (2-2)	171	2 (2-2)	104	2 (2-2)	46	2 (2-2)	0.0781
Children	322	2 (1-2)	172	2 (2-2)	104	2 (1-2)	46	2 (1-2)	0.0157
In households with more than one adult: all adults in employment	302	237 (78.5)	158	121 (76.6)	99	82 (82.8)	45	34 (75.6)	0.4332
At least one household member with a long-term or chronic illness	323	63 (19.5)	173	35 (20.2)	104	15 (14.4)	46	13 (28.3)	0.1343
**Attitudes towards/experiences with SARS-CoV-2 or vaccination**									
How dangerous do you think SARS-CoV-2 is for you/your family? n(%)	323		174		103		46		0.6571
Not at all		13 (4.0)		7 (4.0)		5 (4.9)		1 (2.2)	
Somewhat		33 (10.2)		20 (11.5)		11 (10.7)		2 (4.3)	
Moderately		161 (49.8)		80 (46)		53 (51.5)		28 (60.9)	
Fairly		90 (27.9)		53 (30.5)		27 (26.2)		10 (21.7)	
Very		26 (8.0)		14 (8.0)		7 (6.8)		5 (10.9)	
How dangerous do you think SARS-CoV-2 is for society? n(%)	324		174		104		46		0.1211
Not at all		4 (1.2)		1 (0.6)		3 (2.9)		0 (0.0)	

Somewhat		12 (3.7)		2 (1.1)		6 (5.8)		4 (8.7)	
Moderately		61 (18.8)		30 (17.2)		22 (21.2)		9 (19.6)	
Fairly		174 (53.7)		100 (57.5)		50 (48.1)		24 (52.2)	
Very		73 (22.5)		41 (23.6)		23 (22.1)		9 (19.6)	
I personally know someone (family/ friends/acquaintances) who tested positive for/was ill with/died of SARS-COV-2. n(%)	325	277 (85.2)	174	148 (85.1)	105	90 (85.7)	46	39 (84.8)	0.9846
How important do you think government-mandated restrictive measures to control SARS-CoV-2 are? n (%)	323		174		103		46		0.0698
Not at all		3 (0.9)		1 (0.6)		2 (1.9)		0 (0.0)	
Somewhat		12 (3.7)		4 (2.3)		6 (5.8)		2 (4.3)	
Moderately		51 (15.8)		19 (10.9)		23 (22.3)		9 (19.6)	
Fairly		124 (38.4)		68 (39.1)		35 (34.0)		21 (45.7)	
Very		133 (41.2)		82 (47.1)		37 (35.9)		14 (30.4)	
To what extent do you currently feel limited in your personal life by SARS-CoV-2? n (%)	324		174		104		46		0.4205
Not at all		2 (0.6)		1 (0.6)		1 (1.0)		0 (0.0)	
Somewhat		18 (5.6)		9 (5.2)		6 (5.8)		3 (6.5)	
Moderately		118 (36.4)		62 (35.6)		38 (36.5)		18 (39.1)	
Fairly		130 (40.1)		79 (45.4)		38 (36.5)		13 (28.3)	
Very		56 (17.3)		23 (13.2)		21 (20.2)		12 (26.1)	
Supposing the coronavirus vaccine was approved for children, would you generally be willing to have your child or children vaccinated?	324		173		105		46		0.0047
No		33 (10.2)		12 (6.9)		17 (16.2)		4 (8.7)	

© 2022 Engels G et al. *JAMA Network Open.*

Yes		186 (57.4)		115 (66.5)		48 (45.7)		23 (50.0)	
I do not know		105 (32.4)		46 (26.6)		40 (38.1)		19 (41.3)	
How important do you consider vaccinations in general? n (%)	325		174		105		46		0.094 6
Not at all		1 (0.3)		0 (0.0)		1 (1.0)		0 (0-0)	
Somewhat		4 (1.2)		2 (1.1)		2 (1.9)		0 (0.0)	
Moderately		16 (4.9)		4 (2.3)		9 (8.6)		3 (6.5)	
Fairly		50 (15.4)		21 (12.1)		21 (20)		8 (17.4)	
Very		254 (78.2)		147 (84.5)		72 (68.6)		35 (76.1)	
Are you willing to be vaccinated against the coronavirus?	325		174		105		46		0.038 1
No		12 (3.7)		6 (3.4)		4 (3.8)		2 (4.3)	
Yes		168 (51.7)		90 (51.7)		57 (54.3)		21 (45.7)	
I have already been vaccinated		120 (36.9)		68 (39.1)		31 (29.5)		21 (45.7)	
I have previously had a laboratory- confirmed coronavirus infection		7 (2.2)		6 (3.4)		1 (1.0)			
I do not know		18 (5.5)		4 (2.3)		12 (11.4)		2 (4.3)	
**Children's willingness to participate**									
How well did your child participate? (SAL) Scale 1-5 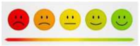 (only for children with at least 1 SAL sample in week 1)	190		116		74				0.242 3
1		1 (0.5)		1 (0.9)		0 (0.0)	.		
2		3 (1.6)		1 (0.9)		2 (2.7)	.		
3		14 (7.4)		6 (5.2)		8 (10.8)	.		
4		33 (17.4)		24 (20.7)		9 (12.2)	.		
5		139 (73.2)		84 (72.4)		55 (74.3)	.		
To what extent did your child like participating? (SAI)	213		133		80				0.847 3

© 2022 Engels G et al. *JAMA Network Open.*

1		5 (2.3)		4 (3)		1 (1.3)	.		
2		7 (3.3)		5 (3.8)		2 (2.5)	.		
3		20 (9.4)		12 (9.0)		8 (10.0)	.		
4		63 (29.6)		37 (27.8)		26 (32.5)	.		
5		118 (55.4)		75 (56.4)		43 (53.8)	.		
How well did your child participate? (AgRT) 1-5	158		117				41		0.0276
1		10 (6.3)		4 (3.4)	.			6 (14.6)	
2		28 (17.7)		17 (14.5)	.			11 (26.8)	
3		42 (26.6)		34 (29.1)	.			8 (19.5)	
4		36 (22.8)		29 (24.8)	.			7 (17.1)	
5		42 (26.6)		33 (28.2)	.			9 (22)	
To what extent did your child like participating? (AgRT) 1-5	175		128				47		0.9141
1		23 (13.1)		16 (12.5)	.			7 (14.9)	
2		47 (26.9)		33 (25.8)	.			14 (29.8)	
3		46 (26.3)		36 (28.1)	.			10 (21.3)	
4		37 (21.1)		27 (21.1)	.			10 (21.3)	
5		22 (12.6)		16 (12.5)	.			6 (12.8)	
**Assessment/Evaluation of the test measures**									
How useful do you think testing children for coronavirus infections is?	324		174		104		46		0.0009
Not at all		3 (0.9)		3 (1.7)					
Somewhat		4 (1.2)		0 (0.0)		1 (1.0)		3 (6.5)	
Moderately		40 (12.3)		13 (7.5)		21 (20.2)		6 (13.0)	
Fairly		150 (46.3)		81 (46.6)		47 (45.2)		22 (47.8)	
Very		127 (39.2)		77 (44.3)		35 (33.7)		15 (32.6)	

© 2022 Engels G et al. *JAMA Network Open.*

How would you rate the frequency of testing, 2 days per week?	325		174		105		46		0.5877
Not often enough		17 (5.2)		10 (5.7)		5 (4.8)		2 (4.3)	
Appropriate		284 (87.4)		152 (87.4)		94 (89.5)		38 (82.6)	
Too often		24 (7.4)		12 (6.9)		6 (5.7)		6 (13)	
How satisfied are you with doing the testing yourself?	324		173		105		46		0.0009
Not at all		5 (1.5)		2 (1.2)		0 (0.0)		3 (6.5)	
Somewhat		5 (1.5)		1 (0.6)		2 (1.9)		2 (4.3)	
Moderately		49 (15.1)		27 (15.6)		9 (8.6)		13 (28.3)	
Fairly		141 (43.5)		73 (42.2)		49 (46.7)		19 (41.3)	
Very		124 (38.3)		70 (40.5)		45 (42.9)		9 (19.6)	
How does testing affect your sense of safety -- that you are protecting yourself and your family from infection?	323		174		103		46		0.6378
Not at all		10 (3.1)		5 (2.9)		5 (4.9)		0 (0.0)	
Somewhat		21 (6.5)		14 (8)		4 (3.9)		3 (6.5)	
Moderately		70 (21.7)		34 (19.5)		26 (25.2)		10 (21.7)	
Fairly		164 (50.8)		87 (50.0)		51 (49.5)		26 (56.5)	
Very		58 (18)		34 (19.5)		17 (16.5)		7 (15.2)	
How stressful is the testing experience for yourself?	319		171		102		46		0.0132
Not at all		122 (38.2)		67 (39.2)		49 (48.0)		6 (13.0)	
Somewhat		130 (40.8)		68 (39.8)		35 (34.3)		27 (58.7)	
Moderately		51 (16.0)		29 (17.0)		13 (12.7)		9 (19.6)	
Fairly		14 (4.4)		6 (3.5)		5 (4.9)		3 (6.5)	
Very		2 (0.6)		1 (0.6)		0 (0.0)		1 (2.2)	

© 2022 Engels G et al. *JAMA Network Open.*

How stressful is the testing for your family overall?	321		172		103		46		0.2995
Not at all		123 (38.3)		63 (36.6)		47 (45.6)		13 (28.3)	
Somewhat		132 (41.1)		76 (44.2)		36 (35)		20 (43.5)	
Moderately		55 (17.1)		28 (16.3)		18 (17.5)		9 (19.6)	
Fairly		9 (2.8)		4 (2.3)		2 (1.9)		3 (6.5)	
Very		2 (0.6)		1 (0.6)		0 (0.0)		1 (2.2)	
How much does the testing affect the organization of your daily routine?	321		171		104		46		0.037
Not at all		117 (36.4)		50 (29.2)		52 (50)		15 (32.6)	
Somewhat		150 (46.7)		87 (50.9)		41 (39.4)		22 (47.8)	
Moderately		36 (11.2)		25 (14.6)		5 (4.8)		6 (13.0)	
Fairly		15 (4.7)		7 (4.1)		5 (4.8)		3 (6.5)	
Very		3 (0.9)		2 (1.2)		1 (1.0)		0 (0.0)	
To what extent do you fear negative consequences for yourself and your family as a result of the measures/testing?	322		173		103		46		0.3354
Not at all		110 (34.2)		64 (37.0)		37 (35.9)		9 (19.6)	
Somewhat		102 (31.7)		50 (28.9)		33 (32.0)		19 (41.3)	
Moderately		72 (22.4)		38 (22.0)		24 (23.3)		10 (21.7)	
Fairly		29 (9)		16 (9.2)		8 (7.8)		5 (10.9)	
Very		9 (2.8)		5 (2.9)		1 (1.0)		3 (6.5)	
What are your views on the planned measures for testing for coronavirus infections?	316		169		102		45		0.0002
Very critical		5 (1.6)		3 (1.8)		2 (2.0)		0 (0.0)	
Somewhat critical		15 (4.7)		6 (3.6)		7 (6.9)		2 (4.4)	
Neutral		50 (15.8)		11 (6.5)		26 (25.5)		13 (28.9)	

© 2022 Engels G et al. *JAMA Network Open.*

Fairly supportive		127 (40.2)		72 (42.6)		35 (34.3)		20 (44.4)	
Very supportive		119 (37.7)		77 (45.6)		32 (31.4)		10 (22.2)	
**Health burden on parents due to the pandemic (worries/fears)**									
Affected by worries about your health	324		174		104		46		0.6795
Not affected		149 (46.0)		84 (48.3)		45 (43.3)		20 (43.5)	
Slightly affected		150 (46.3)		76 (43.7)		53 (51.0)		21 (45.7)	
Strongly affected		25 (7.7)		14 (8.0)		6 (5.8)		5 (10.9)	
Affected by worries about contracting coronavirus	324		174		104		46		0.1105
Not affected		109 (33.6)		67 (38.5)		26 (25.0)		16 (34.8)	
Slightly affected		178 (54.9)		90 (51.7)		66 (63.5)		22 (47.8)	
Strongly affected		37 (11.4)		17 (9.8)		12 (11.5)		8 (17.4)	
Affected by worries of becoming seriously ill if infected with coronavirus	323		173		104		46		0.7043
Not affected		120 (37.2)		67 (38.7)		38 (36.5)		15 (32.6)	
Slightly affected		150 (46.4)		77 (44.5)		52 (50.0)		21 (45.7)	
Strongly affected		53 (16.4)		29 (16.8)		14 (13.5)		10 (21.7)	
Affected by concerns of insufficient capacities for timely treatment in the event of serious illness	322		172		104		46		0.2062
Not affected		178 (55.3)		100 (58.1)		57 (54.8)		21 (45.7)	
Slightly affected		106 (32.9)		54 (31.4)		37 (35.6)		15 (32.6)	
Strongly affected		38 (11.8)		18 (10.5)		10 (9.6)		10 (21.7)	
Affected by worries about infecting others with coronavirus	323		173		104		46		0.2352
Not affected		79 (24.5)		49 (28.3)		22 (21.2)		8 (17.4)	
Slightly affected		166 (51.4)		87 (50.3)		57 (54.8)		22 (47.8)	

© 2022 Engels G et al. *JAMA Network Open.*

Strongly affected		78 (24.1)		37 (21.4)		25 (24)		16 (34.8)	
PHQ-4: above normal	316	43 (13.6)	170	23 (13.5)	101	10 (9.9)	45	10 (22.2)	0.1339

© 2022 Engels G et al. *JAMA Network Open.*

eTable 2. Sociodemographic Characteristics of Participating Childcare Workers, Attitudes Toward the Pandemic/Experience of Self-testing/Perception of Health Burden of the Pandemic, Week 1

N=total, SAL=saliva (mouthwash) samples, AgRT=rapid antigen self-test, PHQ-4=Patient Health Questionnaire 4.

Childcare workers (CCWs)	All participants		*Group 1* (SAL + AgRT)		*Group 2* (SAL)		*Group 3* (AgRT)		
	**N**	**n (%) or Median IQR**	**N**	**n (%) or Median IQR**	**N**	**n (%) or Median IQR**	**N**	**n (%) or Median IQR**	**p-value**
**Socio-demographic data, Health**									
Age, median (IQR)	139	30, 25-46	96	30, 25-45	29	33, 24-52	14	30, 25-37	0.442
Female, n(%)	109	102 (93.6)	78	74 (94.9)	19	18 (94.7)	12	10 (83.3)	0.263
Education	110		79		20		11		0.327
None									
Lower secondary level / basic		9 (8.2)		7 (8.9)				2 (18.2)	
Lower secondary / advanced		39 (35.5)		25 (31.6)		11 (55.0)		3 (27.3)	
Upper secondary level (University entrance level)		60 (54.5)		45 (57.0)		9 (45.0)		6 (54.5)	
Other		2 (1.8)		2 (2.5)					
Chronic health condition (any), n(%)	110	24 (21.8)	79	17 (21.5)	20	4 (20.0)	11	3 (27.3)	0.866
How many people live in your household?	109		80		19		10		0.735
1		11 (10.1)		8 (10.0)		3 (15.8)			
2		52 (47.7)		36 (45.0)		11 (57.9)		5 (50.0)	
3		25 (22.9)		19 (23.8)		4 (21.1)		2 (20.0)	
4		17 (15.6)		13 (16.3)		1 (5.3)		3 (30.0)	
5		4 (3.7)		4 (5.0)					
Persons in household older than 18 years	105		78		19		8		0.542
0		1 (1.0)		1 (1.3)					
1		16 (15.2)		11 (14.1)		4 (21.1)		1 (12.5)	
2		67 (63.8)		49 (62.8)		13 (68.4)		5 (62.5)	
3		14 (13.3)		12 (15.4)		2 (10.5)			
4		5 (4.8)		3 (3.8)				2 (25.0)	
5		2 (1.9)		2 (2.6)					
Persons in household younger than 18 years	80		59		15		6		0.287
0		44 (55.0)		32 (54.2)		11 (73.3)		1 (16.7)	

© 2022 Engels G et al. *JAMA Network Open.*

1		26 (32.5)		19 (32.2)		3 (20.0)		4 (66.7)	
2		9 (11.3)		7 (11.9)		1 (6.7)		1 (16.7)	
3		1 (1.3)		1 (1.7)					
4									
5									
At least one household member with a long-term or chronic medical condition	109	20 (18.3)	79	16 (20.3)	20	3 (15.0)	10	1 (10.0)	0.775
**Attitudes towards/experiences with SARS-CoV-2 or vaccination**									
How dangerous do you think SARS-CoV-2 is for you/your family? n(%)	111		80		20		11		0.310
Not at all		3 (2.7)		3 (3.8)		0 (0.0)			
Somewhat		5 (4.5)		3 (3.8)		0 (0.0)		2 (18.2)	
Moderately		44 (39.6)		30 (37.5)		8 (40.0)		6 (54.5)	
Fairly		51 (45.9)		39 (48.8)		9 (45.0)		3 (27.3)	
Very		8 (7.2)		5 (6.3)		3 (15.0)		0 (0.0)	
How dangerous do you think SARS-CoV-2 is for society? n(%)	111		80		20		11		0.076
Not at all		0 (0.0)		0 (0.0)		0 (0.0)	11	0 (0.0)	
Somewhat		4 (3.6)		3 (3.8)		0 (0.0)	11	1 (9.1)	
Moderately		23 (20.7)		16 (20.0)		2 (10.0)	11	5 (45.5)	
Fairly		62 (55.9)		47 (58.8)		13 (65.0)	11	2 (18.2)	
Very		22 (19.8)		14 (17.5)		5 (25.0)	11	3 (27.3)	
I personally know someone (family/ friends/acquaintances) who tested positive for/was ill with/died of SARS-COV-2. n(%)	111	90 (81.1)	79	67 (84.8)	20	16 (80.0)	12	7 (58.3)	0.090
How important do you think government-mandated restrictive measures to control SARS-CoV-2 are? n (%)	111		80		20		11		0.319
Not at all		1 (0.9)		1 (1.3)		0 (0.0)		0 (0.0)	
Somewhat		5 (4.5)		4 (5.0)		0 (0.0)		1 (9.1)	
Moderately		29 (16.1)		18 (22.5)		6 (30.0)		5 (4.5)	
Fairly		31 (27.9)		21 (26.3)		6 (30.0)		19 (26.1)	
Very		45 (40.5)		36 (45.0)		8 (40.0)		31 (27.9)	

© 2022 Engels G et al. *JAMA Network Open.*

To what extent do you currently feel limited in your personal life by SARS-CoV-2?	111		80		20		11		0.212
Not at all		0 (0.0)		0 (0.0)		0 (0.0)		0 (0.0)	
Somewhat		7 (6.3)		5 (6.3)		1 (5.0)		1 (9.1)	
Moderately		33 (29.7)		27 (33.8)		6 (30.0)		0 (0.0)	
Fairly		43 (38.7)		31 (38.8)		7 (35.0)		5 (45.5)	
Very		28 (25.2)		17 (21.3)		6 (30.0)		5 (45.5)	
Supposing the coronavirus vaccine was approved for children, how important would you think it was that the children in your day care center were vaccinated?	109		79		19		11		0.361
Not at all		10 (9.2)		8 (10.1)		0 (0.0)		2 (18.2)	
Somewhat		11 (10.1)		7 (8.9)		1 (5.3)		3 (27.3)	
Moderately		18 (16.5)		12 (15.2)		4 (21.1)		2 (18.2)	
Fairly		36 (33.0)		26 (32.9)		8 (42.1)		2 (18.2)	
Very		34 (31.2)		26 (32.9)		6 (31.6)		2 (18.2)	
How important do you consider vaccinations in general?	110		79		20		11		0.054
Not at all		1 (0.9)		0 (0.0)		0 (0.0)		1 (9.1)	
Somewhat		4 (3.6)		3 (3.8)		0 (0.0)		1 (9.1)	
Moderately		13 (11.8)		9 (11.4)		1 (5.0)		3 (27.3)	
Fairly		34 (30.9)		27 (34.2)		4 (20.0)		3 (27.3)	
Very		58 (52.7)		40 (50.6)		15 (75.0)		3 (27.3)	
**Experience of self-testing**									
Do you perform self-testing with mouthwash (saliva samples) as part of this study? -yes	99	99 (100.0)	79	79 (100.0)	20	20 (100.0)			
How time-consuming is it for you to perform self-sampling of saliva?	99		79		20				0.340
Not at all		88 (88.9)		68 (86.1)		20 (100.0)			
Somewhat		8 (8.1)		8 (10.1)		0 (0.0)			
Moderately		3 (3.0)		3 (3.8)		0 (0.0)			
Fairly		0 (0.0)		0 (0.0)		0 (0.0)			
Very		0 (0.0)		0 (0.0)		0 (0.0)			

© 2022 Engels G et al. *JAMA Network Open.*

How difficult is it to collect the saliva sample without assistance?	99		79		20				>0.999
Not at all		97 (98.0)		77 (97.5)		20 (100.0)			
Somewhat		0 (0.0)		0 (0.0)		0 (0.0)			
Moderately		2 (2.0)		2 (2.5)		0 (0.0)			
Fairly		0 (0.0)		0 (0.0)		0 (0.0)			
Very		0 (0.0)		0 (0.0)		0 (0.0)			
As part of this study, do you test yourself with rapid antigen tests at home using a nasal swab? - yes	92	92 (100)	80	80 (100.0)			12	12 (100.0)	
How time-consuming is it to perform the AG Rapid test on yourself?	92		80				12		0.846
Not at all		36 (39.1)		30 (37.5)				6 (50.0)	
Somewhat		40 (43.5)		35 (43.8)				5 (41.7)	
Moderately		13 (14.1)		12 (15.0)				1 (8.3)	
Fairly		2 (2.2)		2 (2.5)				0 (0.0)	
Very		1 (1.1)		1 (1.3)				0 (0.0)	
How difficult is it to perform the AG Rapid Test without assistance?	91		79				12		0.040
Not at all		77 (84.6)		69 (87.3)				8 (66.7)	
Somewhat		11 (12.1)		9 (11.4)				2 (16.7)	
Moderately		3 (3.3)		1 (1.3)				2 (16.7)	
Fairly				0 (0.0)				0 (0.0)	
Very				0 (0.0)				0 (0.0)	
**Assessment/Evaluation of the test measures**									
How useful do you think it is for yourself and other daycare workers to perform self-testing for coronavirus infections?	112		80		20		12		0.435
Not at all	112	1 (0.9)	80	1 (1.3)	20	0 (0.0)	12	0 (0.0)	
Somewhat	112	3 (2.7)	80	2 (2.5)	20	0 (0.0)	12	1 (8.3)	
Moderately	112	5 (4.5)	80	4 (5.0)	20	1 (5.0)	12	0 (0.0)	
Fairly	112	30 (26.8)	80	20 (25.0)	20	4 (20.0)	12	6 (50.0)	
Very	112	73 (65.2)	80	53 (66.3)	20	15 (75.0)	12	5 (41.7)	
How useful do you think it is to test daycare children for	111		80		20		11		0.251

© 2022 Engels G et al. *JAMA Network Open.*

infections with the coronavirus?									
Not at all				0 (0.0)		0 (0.0)		0 (0.0)	
Somewhat		4 (3.6)		3 (3.8)		0 (0.0)		1 (9.1)	
Moderately		17 (15.3)		14 (17.5)		1 (5.0)		2 (18.2)	
Fairly		40 (36.0)		26 (32.5)		8 (40.0)		6 (54.5)	
Very		50 (45.0)		37 (46.3)		11 (55.0)		2 (18.2)	
How would you rate the frequency of testing, 2 days per week?	112		80		20		12		0.946
Not often enough		8 (7.1)		7 (8.8)		1 (5.0)		0 (0.0)	
Appropriate		101 (90.2)		70 (87.5)		19 (95.0)		12 (100.0)	
Too often		3 (2.7)		3 (3.8)		0 (0.0)		0 (0.0)	
How does testing affect your sense of safety - that you are protecting yourself and your family from infection?	112		80		20		12		0.718
Not at all		2 (1.8)		2 (2.5)		0 (0.0)		0 (0.0)	
Somewhat		7 (6.3)		6 (7.5)		0 (0.0)		1 (8.3)	
Moderately		21 (18.8)		12 (15.0)		6 (30.0)		3 (25.0)	
Fairly		60 (53.6)		43 (53.8)		10 (50.0)		7 (58.3)	
Very		22 (19.6)		17 (21.3)		4 (20.0)		1 (8.3)	
How stressful is the testing experience for yourself?	112		80		20		12		0.598
Not at all		80 (71.4)		57 (71.3)		16 (80.0)		7 (58.3)	
Somewhat		23 (20.5)		16 (20.0)		4 (20.0)		3 (25.0)	
Moderately		7 (6.3)		5 (6.3)		0 (0.0)		2 (16.7)	
Fairly		2 (1.8)		2 (2.5)		0 (0.0)		0 (0.0)	
Very		0 (0.0)		0 (0.0)		0 (0.0)		0 (0.0)	
How stressful is the testing for your family overall?	110		78		20		12		0.106
Not at all		84 (76.4)		60 (76.9)		17 (85.0)		7 (58.3)	
Somewhat		20 (18.2)		15 (19.2)		3 (15.0)		2 (16.7)	
Moderately		5 (4.5)		2 (2.6)		0 (0.0)		3 (25.0)	
Fairly		1 (0.9)		1 (1.3)		0 (0.0)		0 (0.0)	
Very		0 (0.0)		0 (0.0)		0 (0.0)		0 (0.0)	
How much does the testing affect the organization of your daily routine?	112		80		20		12		0.026
Not at all		58 (51.8)		39 (48.8)		15 (75.0)		4 (33.3)	
Somewhat		45 (40.2)		36 (45.0)		5 (25.0)		4 (33.3)	

© 2022 Engels G et al. *JAMA Network Open.*

Moderately		6 (5.4)		3 (3.8)		0 (0.0)		3 (25.0)	
Fairly		3 (2.7)		2 (2.5)		0 (0.0)		1 (8.3)	
Very		0 (0.0)		0 (0.0)		0 (0.0)		0 (0.0)	
To what extent do you fear negative consequences for yourself and your family as a result of the measures/testing?	110		78		20		12		0.103
Not at all		52 (47.3)		40 (51.3)		9 (45.0)		3 (25.0)	
Somewhat		26 (23.6)		19 (24.4)		6 (30.0)		1 (8.3)	
Moderately		21 (19.1)		13 (16.7)		3 (15.0)		5 (41.7)	
Fairly		4 (3.6)		2 (2.6)		0 (0.0)		2 (16.7)	
Very		7 (6.4)		4 (5.1)		2 (10.0)		1 (8.3)	
What are your views on the planned measures for testing for Coronavirus at baseline?	105		76		19		10		0.746
Very critical		0 (0.0)		0 (0.0)		0 (0.0)		0 (0.0)	
Somewhat critical		6 (5.7)		5 (6.6)		0 (0.0)		1 (10.0)	
Neutral		14 (13.3)		9 (11.8)		3 (15.8)		2 (20.0)	
Fairly supportive		38 (36.2)		26 (34.2)		8 (42.1)		4 (40.0)	
Very supportive		47 (44.8)		36 (47.4)		8 (42.1)		3 (30.0)	
**Health burden on caregivers due to the pandemic (worries/fears)**									
Affected by worries about your health	110		80		20		10		0.560
Not affected		40 (36.4)		29 (36.3)		8 (40.0)		3 (30.0)	
Slightly affected		53 (48.2)		38 (47.5)		8 (40.0)		7 (70.0)	
Strongly affected		17 (15.5)		13 (16.3)		4 (20.0)		0 (0.0)	
Affected by worries about contracting coronavirus	110		80		20		10		0.707
Not affected		20 (18.2)		13 (16.3)		5 (25.0)		2 (20.0)	
Slightly affected		64 (58.2)		49 (61.3)		9 (45.0)		6 (60.0)	
Strongly affected		26 (23.6)		18 (22.5)		6 (30.0)		2 (20.0)	
Affected by worries of becoming seriously ill if infected with coronavirus	109		79		20		10		0.290
Not affected		28 (25.7)		17 (21.5)		7 (35.0)		4 (40.0)	
Slightly affected		48 (44.0)		37 (46.8)		6 (30.0)		5 (50.0)	
Strongly affected		33 (30.3)		25 (31.6)		7 (35.0)		1 (10.0)	
Affected by concerns of insufficient capacities for timely	110		80		20		10		0.191

treatment in the event of serious illness									
Not affected		41 (37.3)		29 (36.2)		5 (25.0)		7 (70.0)	
Slightly affected		39 (35.5)		30 (37.5)		8 (40.0)		1 (10.0)	
Strongly affected		30 (27.3)		21 (26.3)		7 (35.0)		2 (20.0)	
Affected by worries about infecting others with coronavirus	110		80		20		10		0.992
Not affected		10 (9.1)		7 (8.8)		2 (10.0)		1 (10.0)	
Slightly affected		37 (33.6)		28 (35.0)		6 (30.0)		3 (30.0)	
Strongly affected		63 (57.3)		45 (56.3)		12 (60.0)		6 (60.0)	
PHQ-4 above normal	104	20 (19.2)	76	11 (14.5)	18	4 (22.2)	10	5 (50.0)	0.030

© 2022 Engels G et al. *JAMA Network Open.*

eTable 3. Comparison of Children's Health Status/Readiness to Participate and Parents' Perceptions of Respiratory Surveillance Measures/Attitudes Toward the Pandemic at Weeks 1 and 12

N=total, SAL=saliva (mouth rinsing fluid) samples, AgRT= antigen rapid self-test, CBCL=Child Behavior Checklist, PHQ-4=Patient Health Questionnaire 4.

	**All Participants**				**Group 1 (SAL + AgRT)**				**Group 2 (SAL)**				**Group 3 (AgRT)**			
	**N**	**Week 1**	**Week 12**	**p-value**	**N**	**Week 1**	**Week 12**	**p-value**	**N**	**Week 1**	**Week 12**	**p-value**	**N**	**Week 1**	**Week 12**	**p-value**
**Children**																
CBCL indicative of problematic behavior- yes	245	20 (8.2)	15 (6.1)	0.359	143	12 (8.4)	8 (5.6)	0.344	68	4 (5.9)	5 (7.4)	>0.999	34	4 (11.8)	2 (5.9)	0.625
How well did your child participate from your point of view? (Saliva sample)	112			0.745	77			>0.999	35			>0.999				
Poorly		12 (10.7)	10 (8.9)			6 (7.8)	5 (6.5)		35	6 (17.1)	5 (14.3)					
Well		100 (89.3)	102 (91.1)			71 (92.2)	72 (93.5)		35	29 (82.9)	30 (85.7)					
To what extent did your child like participating from your point of view? (Saliva sample)	141			0.169	99			0.481	42			0.289				
Did not like it		17 (12.1)	25 (17.7)			11 (11.1)	15 (15.2)			6 (14.3)	10 (23.8)					
Liked it		124 (87.9)	116 (82.3)			88 (88.9)	84 (84.8)			36 (85.7)	32 (76.2)					
How well did your child participate from your point of view? (Rapid test)	93			0.701	69			0.664					24			>0.999
Poorly		47 (50.5)	50 (53.8)			31 (44.9)	34 (49.3)							16 (66.7)	16 (66.7)	
Well		46 (49.5)	43 (46.2)			38 (55.1)	35 (50.7)							8 (33.3)	8 (33.3)	
To what extent did your child like participating from your point of view? (Rapid test)	125			0.073	95			0.136					30			0.508

© 2022 Engels G et al. *JAMA Network Open.*

	All Participants				Group 1 (SAL + AgRT)				Group 2 (SAL)				Group 3 (AgRT)			
	N	Week 1	Week 12	p-value	N	Week 1	Week 12	p-value	N	Week 1	Week 12	p-value	N	Week 1	Week 12	p-value
Did not like it		80 (64.0)	92 (73.6)			59 (62.1)	68 (71.6)							21 (70)	24 (80)	
Liked it		45 (36.0)	33 (26.4)			36 (37.9)	27 (28.4)							9 (30)	6 (20)	
**Parents**																
How dangerous do you think the coronavirus is for you and your family?	223			0.013	136			0.043	59			0.388	28			0.687
Not dangerous (1-3)		140 (62.8)	158 (70.9)			82 (60.3)	94 (69.1)			40 (67.8)	44 (74.6)			18 (64.3)	20 (71.4)	
Dangerous (4-5)		83 (37.2)	65 (29.1)			54 (39.7)	42 (30.9)			19 (32.2)	15 (25.4)			10 (35.7)	8 (28.6)	
How dangerous do you think the coronavirus is for society?	223			<0.001	136			0.019	59			0.006	28			0.727
Not dangerous (1-3)		48 (21.5)	73 (32.7)			25 (18.4)	38 (27.9)			13 (22)	23 (39)			10 (35.7)	12 (42.9)	
Dangerous (4-5)		175 (78.5)	150 (67.3)			111 (81.6)	98 (72.1)			46 (78)	36 (61)			18 (64.3)	16 (57.1)	
I personally know someone (family/ friends/acquaintances) who tested positive for/was ill with /died of SARS-COV-2	223	192 (86.1)	178 (79.8)	0.009	136	116 (85.3)	114 (83.8)	0.774	59	52 (88.1)	44 (74.6)	0.008	28	24 (85.7)	20 (71.4)	0.219
How important do you think government-mandated restrictive measures to control SARS-CoV-2 are?	223			0.508	136			0.625	59			>0.999	28			>0.999
Not important (1-2)		9 (4)	6 (2.7)			4 (2.9)	2 (1.5)			3 (5.1)	3 (5.1)			2 (7.1)	1 (3.6)	
Important (3-5)		214 (96)	217 (97.3)			132 (97.1)	134 (98.5)			56 (94.9)	56 (94.9)			26 (92.9)	27 (96.4)	
To what extent do you currently feel limited in your personal life by the coronavirus?	223			<0.001	136			<0.001	59			<0.001	28			0.002
Not limited (1-3)		98 (43.9)	184 (82.5)			60 (44.1)	116 (85.3)			26 (44.1)	46 (78)			12 (42.9)	22 (78.6)	
Limited (4-5)		125 (56.1)	39 (17.5)			76 (55.9)	20 (14.7)			33 (55.9)	13 (22)			16 (57.1)	6 (21.4)	

© 2022 Engels G et al. *JAMA Network Open.*

	All Participants				Group 1 (SAL + AgRT)				Group 2 (SAL)				Group 3 (AgRT)			
	N	Week 1	Week 12	p-value	N	Week 1	Week 12	p-value	N	Week 1	Week 12	p-value	N	Week 1	Week 12	p-value
Not important (1-2)		3 (1.3)	2 (0.9)			2 (1.5)				1 (1.7)	1 (1.7)			0 (0.0)	1 (3.6)	
Important (3-5)		220 (98.7)	221 (99.1)			134 (98.5)	136 (100)			58 (98.3)	58 (98.3)			28 (100)	27 (96.4)	
Are you willing to be vaccinated against coronavirus?	224			<0.001	136			<0.001	60			<0.001	28			n.a.
No		6 (2.7)	6 (2.7)			4 (2.9)	2 (1.5)			1 (1.7)	4 (6.7)			1 (3.6)	0 (0.0)	
Yes		120 (53.6)	48 (21.4)			73 (53.7)	28 (20.6)			34 (56.7)	15 (25)			13 (46.4)	5 (17.9)	
I have already been vaccinated		82 (36.6)	161 (71.9)			53 (39)	103 (75.7)			16 (26.7)	37 (61.7)			13 (46.4)	21 (75)	
I have previously had a laboratory-confirmed coronavirus infection		3 (1.3)	1 (0.4)			3 (2.2)	1 (0.7)							0 (0.0)	0 (0.0)	
I do not know		13 (5.8)	8 (3.6)			3 (2.2)	2 (1.5)			9 (15)	4 (6.7)			1 (3.6)	2 (7.1)	
How useful do you think the tests are?	221			>0.999	135			0.289	59			0.375	27			>0.999
Not useful (1-3)		29 (13.1)	30 (13.6)			11 (8.1)	13 (9.6)			12 (20.3)	8 (13.6)			6 (22.2)	9 (33.3)	
Useful (4-5)		192 (86.9)	191 (86.4)			124 (91.9)	122 (90.4)			47 (79.7)	51 (86.4)			21 (77.8)	18 (66.7)	
How would you rate the frequency of testing, 2 days per week?	223			0.352	135			0.905	60			n.a.	28			n.a.
Not often enough		10 (4.5)	6 (2.7)			6 (4.4)	6 (4.4)			2 (3.3)				2 (7.1)	0 (0.0)	
Appropriate		200 (89.7)	203 (91)			121 (89.6)	122 (90.4)			55 (91.7)	56 (93.3)			24 (85.7)	25 (89.3)	
Too often		13 (5.8)	14 (6.3)			8 (5.9)	7 (5.2)			3 (5)	4 (6.7)			2 (7.1)	3 (10.7)	
How satisfied are you with doing the testing yourself?	222			0.382	134			0.845	60			0.146	28			0.508
Not satisfied (1-3)		42 (18.9)	49 (22.1)			24 (17.9)	22 (16.4)			8 (13.3)	14 (23.3)			10 (35.7)	13 (46.4)	

© 2022 Engels G et al. *JAMA Network Open.*

	All Participants				Group 1 (SAL + AgRT)				Group 2 (SAL)				Group 3 (AgRT)			
	N	Week 1	Week 12	p-value	N	Week 1	Week 12	p-value	N	Week 1	Week 12	p-value	N	Week 1	Week 12	p-value
Satisfied (4-5)		180 (81.1)	173 (77.9)			110 (82.1)	112 (83.6)			52 (86.7)	46 (76.7)			18 (64.3)	15 (53.6)	
How does testing affect your sense of security -- that you are protecting yourself and your family from infection?	222			0.332	135			>0.999	59			>0.999	28			0.109
No effect (1-3)		66 (29.7)	74 (33.3)			40 (29.6)	41 (30.4)			19 (32.2)	20 (33.9)			7 (25)	13 (46.4)	
It has an effect (4-5)		156 (70.3)	148 (66.7)			95 (70.4)	94 (69.6)			40 (67.8)	39 (66.1)			21 (75)	15 (53.6)	
How stressful is the testing experience for yourself?	218			>0.999	131			0.687	59			0.375	28			0.625
Not stressful (1-3)		209 (95.9)	208 (95.4)			127 (96.9)	125 (95.4)			55 (93.2)	58 (98.3)			27 (96.4)	25 (89.3)	
Stressful (4-5)		9 (4.1)	10 (4.6)			4 (3.1)	6 (4.6)			4 (6.8)	1 (1.7)			1 (3.6)	3 (10.7)	
How stressful is the testing for your family overall?	219			0.057	132			0.180	59			>0.999	28			n.a.
Not stressful (1-3)		216 (98.6)	208 (95)			130 (98.5)	125 (94.7)			58 (98.3)	58 (98.3)			28 (100)	25 (89.3)	
Stressful (4-5)		3 (1.4)	11 (5)			2 (1.5)	7 (5.3)			1 (1.7)	1 (1.7)			0 (0.0)	3 (10.7)	
How much does the testing affect the organization of your daily routine?	220			0.774	132			0.219	60			0.625	28			>0.999
No impairement (1-3)		210 (95.5)	208 (94.5)			127 (96.2)	123 (93.2)			56 (93.3)	58 (96.7)			27 (96.4)	27 (96.4)	
Impairement (4-5)		10 (4.5)	12 (5.5)			5 (3.8)	9 (6.8)			4 (6.7)	2 (3.3)			1 (3.6)	1 (3.6)	
To what extent do you fear negative consequences for yourself and your family as a result of the measures/testing?	218			<0.001	132			0.004	58			0.687	28			0.031
No fear (1-3)		191 (87.6)	212 (97.2)			116 (87.9)	129 (97.7)			54 (93.1)	56 (96.6)			21 (75)	27 (96.4)	

© 2022 Engels G et al. *JAMA Network Open.*

	All Participants				Group 1 (SAL + AgRT)				Group 2 (SAL)				Group 3 (AgRT)			
	**N**	**Week 1**	**Week 12**	**p-value**	**N**	**Week 1**	**Week 12**	**p-value**	**N**	**Week 1**	**Week 12**	**p-value**	**N**	**Week 1**	**Week 12**	**p-value**
Fear (4-5)		27 (12.4)	6 (2.8)			16 (12.1)	3 (2.3)			4 (6.9)	2 (3.4)			7 (25)	1 (3.6)	
What are your views on the planned measures for testing for coronavirus infections	217			0.344	131			>0.999	59			0.625	27			n.a.
Critical (1-2)		10 (4.6)	6 (2.8)			5 (3.8)	4 (3.1)			4 (6.8)	2 (3.4)			1 (3.7)	0 (0.0)	
Supportive (3-5)		207 (95.4)	211 (97.2)			126 (96.2)	127 (96.9)			55 (93.2)	57 (96.6)			26 (96.3)	27 (100)	
Affected by worries about your health	222			0.001	135			0.006	59			n.a.	28			0.572
Not affected		92 (41.4)	114 (51.4)			57 (42.2)	75 (55.6)			25 (42.4)	28 (47.5)			10 (35.7)	11 (39.3)	
Slightly affected		111 (50)	103 (46.4)			67 (49.6)	56 (41.5)			30 (50.8)	31 (52.5)			14 (50)	16 (57.1)	
Strongly affected		19 (8.6)	5 (2.3)			11 (8.1)	4 (3)			4 (6.8)				4 (14.3)	1 (3.6)	
Affected by worries about contracting coronavirus	222			0.005	135			0.095	59			0.020	28			0.406
Not affected		68 (30.6)	91 (41)			47 (34.8)	58 (43)			12 (20.3)	24 (40.7)			9 (32.1)	9 (32.1)	
Slightly affected		125 (56.3)	116 (52.3)			73 (54.1)	70 (51.9)			41 (69.5)	31 (52.5)			11 (39.3)	15 (53.6)	
Strongly affected		29 (13.1)	15 (6.8)			15 (11.1)	7 (5.2)			6 (10.2)	4 (6.8)			8 (28.6)	4 (14.3)	
Affected by worries of becoming seriously ill if infected with coronavirus	220			<0.001	133			0.004	59			0.066	28			0.162
Not affected		77 (35)	102 (46.4)			48 (36.1)	61 (45.9)			21 (35.6)	30 (50.8)			8 (28.6)	11 (39.3)	
Slightly affected		102 (46.4)	99 (45)			62 (46.6)	63 (47.4)			28 (47.5)	22 (37.3)			12 (42.9)	14 (50)	
Strongly affected		41 (18.6)	19 (8.6)			23 (17.3)	9 (6.8)			10 (16.9)	7 (11.9)			8 (28.6)	3 (10.7)	
Affected by concerns of insufficient capacities for timely treatment in the event of serious illness	221			<0.001	134			<0.001	59			0.134	28			0.072

© 2022 Engels G et al. *JAMA Network Open.*

	All Participants				Group 1 (SAL + AgRT)				Group 2 (SAL)				Group 3 (AgRT)			
	N	Week 1	Week 12	p-value	N	Week 1	Week 12	p-value	N	Week 1	Week 12	p-value	N	Week 1	Week 12	p-value
Not affected		117 (52.9)	155 (70.1)			76 (56.7)	104 (77.6)			30 (50.8)	38 (64.4)			11 (39.3)	13 (46.4)	
Slightly affected		73 (33)	58 (26.2)			43 (32.1)	27 (20.1)			22 (37.3)	18 (30.5)			8 (28.6)	13 (46.4)	
Strongly affected		31 (14)	8 (3.6)			15 (11.2)	3 (2.2)			7 (11.9)	3 (5.1)			9 (32.1)	2 (7.1)	
Affected by worries about infecting others with coronavirus	221			0.001	134			0.013	59			0.154	28			0.168
Not affected		53 (24)	70 (31.7)			37 (27.6)	46 (34.3)			11 (18.6)	19 (32.2)			5 (17.9)	5 (17.9)	
Slightly affected		112 (50.7)	120 (54.3)			67 (50)	73 (54.5)			34 (57.6)	31 (52.5)			11 (39.3)	16 (57.1)	
Strongly affected		56 (25.3)	31 (14.0)			30 (22.4)	15 (11.2)			14 (23.7)	9 (15.3)			12 (42.9)	7 (25.0)	
PHQ-4 above normal	215	31 (14.4)	22 (10.2)	0.150	130	17 (13.1)	16 (12.3)	>0.999	57	6 (10.5)	4 (7.0)	0.625	28	8 (28.6)	2 (7.1)	0.031

© 2022 Engels G et al. *JAMA Network Open.*

eTable 4. Comparison of Perceptions of Respiratory Surveillance Measures and Attitudes Toward the Pandemic of Participating Childcare Workers at Weeks 1 and 12

N=total, SAL=saliva (mouth rinsing fluid) samples, AgRT= antigen rapid self-test, PHQ-4=Patient Health Questionnaire 4.

	All Participants				Group 1 (SAL + AgRT)				Group 2 (SAL)				Group 3 (AgRT)			
	**N**	**Week 1**	**Week 12**	**p-value**	**N**	**Week 1**	**Week 12**	**p-value**	**N**	**Week 1**	**Week 12**	**p-value**	**N**	**Week 1**	**Week 12**	**p-value**
How dangerous do you think the coronavirus is for you and your family?	62			0.001	43			0.001	12			>0.999	7			>0.999
Not dangerous (1-3)		23 (37.1)	39 (62.9)			14 (32.6)	28 (65.1)			4 (33.3)	5 (41.7)			5 (71.4)	6 (85.7)	
Dangerous (4-5)		39 (62.9)	23 (37.1)			29 (67.4)	15 (34.9)			8 (66.7)	7 (58.3)			2 (28.6)	1 (14.3)	
How dangerous do you think the coronavirus is for society?	62			0.039	43			0.180	12			n.a.	7			0.500
Not dangerous (1-3)		13 (21.0)	21 (33.9)			10 (23.3)	15 (34.9)				1 (8.3)			3 (42.9)	5 (71.4)	
Dangerous (4-5)		49 (79.0)	41 (66.1)			33 (76.7)	28 (65.1)			12 (100)	11 (91.7)			4 (57.1)	2 (28.6)	
I personally know someone (family/ friends/acquaintances) who tested positive for/was ill with/died of SARS-CoV-2	62	52 (83.9)	50 (80.6)	0.687	43	38 (88.4)	37 (86.0)	>0.999	12	10 (83.3)	10 (83.3)	>0.999	7	4 (57.1)	3 (42.9)	>0.999
How important do you think government-mandated restrictive measures to control SARS-CoV-2 are?	62			>0.999	43			n.a.	12			n.a.	7			>0.999
Not important (1-2)		1 (1.6)	1 (1.6)											1 (14.3)	1 (14.3)	
Important (3-5)		61 (98.4)	61 (98.4)			43 (100)	43 (100)			12 (100)	12 (100)			6 (85.7)	6 (85.7)	
To what extent do you currently feel limited in your personal life by the coronavirus?	62			0.011	43			0.052	12			0.687	7			
Not limited (1-3)		21 (33.9)	36 (58.1)			16 (37.2)	26 (60.5)			5 (41.7)	7 (58.3)				3 (42.9)	

© 2022 Engels G et al. *JAMA Network Open.*

	All Participants				Group 1 (SAL + AgRT)				Group 2 (SAL)				Group 3 (AgRT)			
	**N**	**Week 1**	**Week 12**	**p-value**	**N**	**Week 1**	**Week 12**	**p-value**	**N**	**Week 1**	**Week 12**	**p-value**	**N**	**Week 1**	**Week 12**	**p-value**
Limited (4-5)		41 (66.1)	26 (41.9)			27 (62.8)	17 (39.5)			7 (58.3)	5 (41.7)			7 (100)	4 (57.1)	
Supposing the coronavirus vaccination was approved for children, how important would you think it was that the children in your daycare were vaccinated against the Coronavirus?	61			0.065	42			0.375	12			0.625	7			0.500
Not important (1-2)		12 (19.7)	19 (31.1)			8 (19.0)	11 (26.2)			1 (8.3)	3 (25.0)			3 (42.9)	5 (71.4)	
Important (3-5)		49 (80.3)	42 (68.9)			34 (81.0)	31 (73.8)			11 (91.7)	9 (75.0)			4 (57.1)	2 (28.6)	
How important do you think vaccinations are in general?	61			>0.999	42			n.a.	12			n.a.	7			n.a.
Not important (1-2)		1 (1.6)	1 (1.6)				1 (2.4)							1 (14.3)		
Important (3-5)		60 (98.4)	60 (98.4)			42 (100)	41 (97.6)			12 (100)	12 (100)			6 (85.7)	7 (100)	
How time-consuming is it to take your own saliva sample?	54				42				12							
Not time-consuming (1-3)		54 (100)	54 (100)			42 (100)	42 (100)			12 (100)	12 (100)					
Time-consuming (4-5)																
How difficult is it to collect the saliva sample without assistance?	54				42				12							
Not difficult (1-3)		54 (100)	54 (100)			42 (100)	42 (100)			12 (100)	12 (100)					
Difficult (4-5)																
How time-consuming is it to perform the AG Rapid test on yourself?	50				43								7			
Not time-consuming (1-3)		47 (94.0)	50 (100)			40 (93.0)	43 (100)							7 (100)	7 (100)	
Time-consuming (4-5)		3 (6.0)				3 (7.0)										

© 2022 Engels G et al. *JAMA Network Open.*

	All Participants				Group 1 (SAL + AgRT)				Group 2 (SAL)				Group 3 (AgRT)			
	**N**	**Week 1**	**Week 12**	**p-value**	**N**	**Week 1**	**Week 12**	**p-value**	**N**	**Week 1**	**Week 12**	**p-value**	**N**	**Week 1**	**Week 12**	**p-value**
How difficult is it to take the AG Rapid Test without assistance?	50				43								7			
Not difficult (1-3)		50 (100)	50 (100)			43 (100)	43 (100)							7 (100)	7 (100)	
Difficult (4-5)																
How would you rate the frequency of testing, 2 days per week?	62			0.107	43			0.055	12			n.a.	7			n.a.
Not often enough		6 (9.7)	3 (4.8)			6 (14.0)	2 (4.7)				1 (8.3)					
Appropriate		54 (87.1)	53 (85.5)			35 (81.4)	36 (83.7)			12 (100)	11 (91.7)			7 (100)	6 (85.7)	
Too often		2 (3.2)	6 (9.7)			2 (4.7)	5 (11.6)								1 (14.3)	
How does testing affect your sense of security -- that you are protecting yourself and your family from infection?	61			0.388	43			0.508	12			n.a.	6			>0.999
No effect (1-3)		5 (8.2)	9 (14.8)			4 (9.3)	7 (16.3)				1 (8.3)			1 (16.7)	1 (16.7)	
It has an effect (4-5)		56 (91.8)	52 (85.2)			39 (90.7)	36 (83.7)			12 (100)	11 (91.7)			5 (83.3)	5 (83.3)	
How stressful is the testing experience for yourself?	62			>0.999	43			>0.999	12			n.a.	7			>0.999
Not stressful (1-3)		56 (90.3)	56 (90.3)			38 (88.4)	39 (90.7)			12 (100)	12 (100)			6 (85.7)	5 (71.4)	
Stressful (4-5)		6 (9.7)	6 (9.7)			5 (11.6)	4 (9.3)							1 (14.3)	2 (28.6)	
How stressful is the testing for your family overall?	60			>0.999	42			>0.999	11			n.a.	7			>0.999
Not stressful (1-3)		56 (93.3)	55 (91.7)			39 (92.9)	39 (92.9)			11 (100)	11 (100)			6 (85.7)	5 (71.4)	
Stressful (4-5)		4 (6.7)	5 (8.3)			3 (7.1)	3 (7.1)							1 (14.3)	2 (28.6)	
How much does the testing affect the organization of your daily routine?	62			>0.999	43			>0.999	12			n.a.	7			>0.999
No impairement (1-3)		59 (95.2)	59 (95.2)			41 (95.3)	42 (97.7)			12 (100)	12 (100)			6 (85.7)	5 (71.4)	

© 2022 Engels G et al. *JAMA Network Open.*

	All Participants				Group 1 (SAL + AgRT)				Group 2 (SAL)				Group 3 (AgRT)			
	**N**	**Week 1**	**Week 12**	**p-value**	**N**	**Week 1**	**Week 12**	**p-value**	**N**	**Week 1**	**Week 12**	**p-value**	**N**	**Week 1**	**Week 12**	**p-value**
Impairment (4-5)		3 (4.8)	3 (4.8)			2 (4.7)	1 (2.3)							1 (14.3)	2 (28.6)	
To what extent do you fear negative consequences for yourself and your family as a result of the measures/testing?	60			0.219	41			n.a.	12			n.a.	7			>0.999
No fear (1-3)		55 (91.7)	59 (98.3)			39 (95.1)	41 (100)			11 (91.7)	12 (100)			5 (71.4)	6 (85.7)	
Fear (4-5)		5 (8.3)	1 (1.7)			2 (4.9)				1 (8.3)				2 (28.6)	1 (14.3)	
What are your views on the planned measures for testing for coronavirus infections?	58			>0.999	40			>0.999	12			n.a.	6			>0.999
Critical (1-2)		3 (5.2)	4 (6.9)			2 (5.0)	2 (5.0)				1 (8.3)			1 (16.7)	1 (16.7)	
Supportive (3-5)		55 (94.8)	54 (93.1)			38 (95.0)	38 (95.0)			12 (100)	11 (91.7)			5 (83.3)	5 (83.3)	
Affected by worries about your health	62			0.284	43			0.198	12			0.423	7			>0.999
Not affected		21 (33.9)	23 (37.1)			14 (32.6)	17 (39.5)			5 (41.7)	3 (25.0)			2 (28.6)	3 (42.9)	
Slightly affected		32 (51.6)	35 (56.5)			22 (51.2)	23 (53.5)			5 (41.7)	8 (66.7)			5 (71.4)	4 (57.1)	
Strongly affected		9 (14.5)	4 (6.5)			7 (16.3)	3 (7.0)			2 (16.7)	1 (8.3)					
Affected by worries about contracting coronavirus	62			0.010	43			0.023	12			0.513	7			0.368
Not affected		10 (16.1)	17 (27.4)			6 (14.0)	10 (23.3)			2 (16.7)	4 (33.3)			2 (28.6)	3 (42.9)	
Slightly affected		34 (54.8)	38 (61.3)			24 (55.8)	29 (67.4)			7 (58.3)	6 (50.0)			3 (42.9)	3 (42.9)	
Strongly affected		18 (29.0)	7 (11.3)			13 (30.2)	4 (9.3)			3 (25.0)	2 (16.7)			2 (28.6)	1 (14.3)	
Affected by worries of becoming seriously ill if infected with coronavirus	60			0.041	42			0.035	12			0.572	6			n.a.
Not affected		13 (21.7)	18 (30.0)			6 (14.3)	11 (26.2)			5 (41.7)	5 (41.7)			2 (33.3)	2 (33.3)	
Slightly affected		26 (43.3)	30 (50.0)			20 (47.6)	22 (52.4)			3 (25.0)	4 (33.3)			3 (50.0)	4 (66.7)	

© 2022 Engels G et al. *JAMA Network Open.*

	All Participants				Group 1 (SAL + AgRT)				Group 2 (SAL)				Group 3 (AgRT)			
	**N**	**Week 1**	**Week 12**	**p-value**	**N**	**Week 1**	**Week 12**	**p-value**	**N**	**Week 1**	**Week 12**	**p-value**	**N**	**Week 1**	**Week 12**	**p-value**
Strongly affected		21 (35.0)	12 (20.0)			16 (38.1)	9 (21.4)			4 (33.3)	3 (25.0)			1 (16.7)		
Affected by concerns of insufficient capacities for timely treatment in the event of serious illness	62			0.040	43			0.014	12			0.846	7			0.223
Not affected		22 (35.5)	33 (53.2)			15 (34.9)	28 (65.1)			3 (25.0)	3 (25.0)			4 (57.1)	2 (28.6)	
Slightly affected		24 (38.7)	21 (33.9)			18 (41.9)	11 (26.6)			5 (41.7)	6 (50.0)			1 (14.3)	4 (57.1)	
Strongly affected		16 (25.8)	8 (12.9)			10 (23.3)	4 (9.3)			4 (33.3)	3 (25.0)			2 (28.6)	1 (14.3)	
Affected by worries about infecting others with coronavirus	62			<0.001	43			0.002	12			n.a.	7			0.157
Not affected		4 (6.5)	11 (17.7)			3 (7.0)	9 (20.9)			4 (33.3)	1 (8.3)			1 (14.3)	1 (14.3)	
Slightly affected		21 (33.9)	32 (51.6)			15 (34.9)	23 (53.5)				5 (41.7)			2 (28.6)	4 (57.1)	
Strongly affected		37 (59.7)	19 (30.6)			25 (58.1)	11 (25.6)			8 (66.7)	6 (50.0)			4 (57.1)	2 (28.6)	
PHQ-4 above Normal	59	10 (16.9)	6 (10.2)	0.289	41	5 (12.2)	2 (4.9)	0.375	11	3 (27.3)	3 (27.3)	>0.999	7	2 (28.6)	1 (14.3)	>0.999

© 2022 Engels G et al. *JAMA Network Open.*

eTable 5. Factors Influencing Children's Unsuccessful Long-term Participation

(n=335 with questionnaire responses; 114 of which unsuccessful, 221 successful; univariable and multivariable analyses).

Likelihood of unsuccessful childrens‘ participation in respiratory surveillance. (Drop-out or fewer than 60% of scheduled tests performed for participant)						
	**Univariable analyses***		**Multivariable analyses***			
			**Model 1**		**Model 2**	
	**OR (95%-CI)**	**p-value**	**OR (95%-CI)**	**p-value**	**OR (95%-CI)**	**p-value**
**Group organization, socio-demographic data**						
Group		0.0084		0.0607		0.0758
Group 1 (Reference) (saliva sample +AG-Rapid test)	1.00		1.00		1.00	
Group 2 (saliva sample only)	0.57 (0.33.0.99)		0.51 (0.28-0.91)		0.50 (0.28-0.91)	
Group 3 (AG-Rapid test only)	2.01 (1.08-3.76)		1.27 (0.59-2.74)		1.21 (0.53-2.74)	
Child Age (ref: 2 years) per increase of 1 unit	0.58 (0.42-0.80)	0.0005	0.61 (0.43-0.88)	0.0088	0.60 (0.41-0.88)	0.0092
Child with chronic health condition	0.68 (0.37-1.25)	0.1965	0.73 (0.41-1.29)	0.2944	0.75 (0.44-1.30)	0.3381
Age of parents (ref=28 years) per 1 unit increase	0.16 (0.04-0.62)	0.0095	0.26 (0.06-1.16)	0.0801	0.27 (0.06-1.19)	0.0860
Employment situation (full time vs. other)	1.21 (0.73-2.03)	0.4662	1.34(0.77-2.33)	0.2979	1.33 (0.76.-2.30)	0.3187
**Attitudes towards SARS-COV-2**						
Assessment of SARS-CoV-2 as dangerous to self or family (Ref: not dangerous).	0.85 (0.43-1.67)	0.6304			0.85 (0.41-1.78)	0.6281

*generalized estimation equation with random effects (cluster= parents).

Model 1: inclusion of sociodemographic variables and group membership only if significant at the 10% level in univariable analysis (Chi² test), plus inclusion of sociodemographic variables considered as contextual predictors.

Model 2: as Model 1, plus inclusion of parents’ attitudes.

© 2022 Engels G et al. *JAMA Network Open.*

eTable 6. Factors Influencing Successful Participation of Childcare Workers

(n=109 with questionnaire responses, 67 of which successful, 42 unsuccessful; univariable and multivariable analyses).

Likelihood of unsuccessful CCWs' participation in respiratory surveillance. (Drop-out, or fewer than 60% of scheduled tests performed for participant)						
	**Univariable analyses**		**Multivariable analyses***			
			**Model 1**		**Model 2**	
	**OR (95%-CI)**	**p-Wert**	**OR (95%-CI)**	**p-Wert**	**OR (95%-CI)**	**p-Wert**
**Study group, socio-demographic data**				0.089		0.107
Group 1 (Reference) (saliva sample +AG-Rapid self-test)	1.00		1.00		1.00	
Group 2 (saliva sample only)	0.23 (0.06-0.84)	0.027	0.22 (0.06-0.86)	0.029	0.23 (0.06-0.93)	0.039
Group 3 (AG-Rapid self test only)	1.08 (0.30-3.83)	0.907	0.98 (0.26-3.69)	0.980	0.60 (0.14-2.53)	0.490
Age (per 1 unit increase)	0.95 (0.92-0.98)	0.002	0.95 (0.92-0.99)	0.005	0.95 (0.92-0.99)	0.011
Chronic health condition	0.45 (0.16-1.26)	0.13	0.54 (0.18-1.60)	0.266	0.67 (0.21-2.08)	0.486
**Attitudes/Opinions**						
Assessment of SARS-CoV-2 as dangerous to self or family (Ref: not dangerous).	0.28 (0.12-0.63)	0.002			0.37 (0.15-0.92)	0.033
Attitude towards vaccinations in general (Ref: not important)	0.14 (0.02-1.34)	0.088			0.225 (0.02-2.63)	0.234

*Model 1: inclusion of sociodemographic variables / group membership only if significant at the 10% level in univariable analysis (Chi² test).

Model 2: as Model 1, plus inclusion of participants' attitudes/opinions.

© 2022 Engels G et al. *JAMA Network Open.*

eFigure 1. Flow Diagram

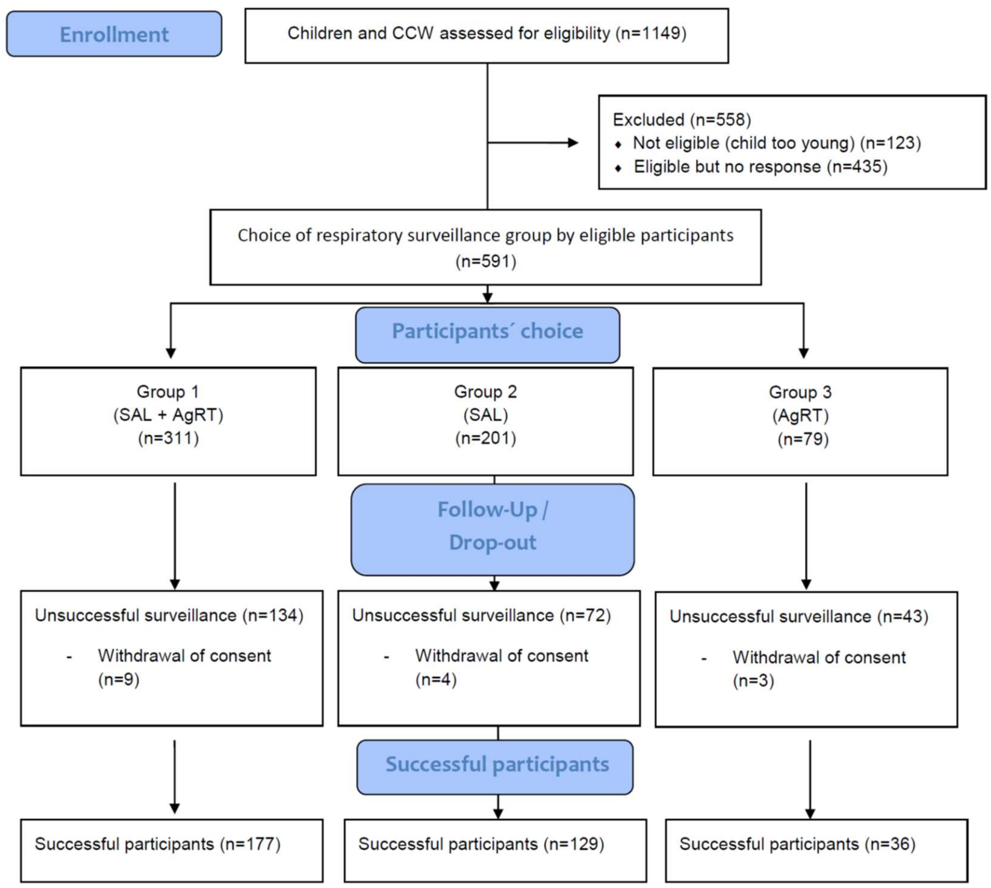

All data in the Flow chart refer to the 12-week surveillance period. CCW= child care workers, SAL=saliva testing, AgRT= antigen rapid self-test

© 2022 Engels G et al. *JAMA Network Open.*

eFigure 2. Age-Adjusted Incidences in the City and District of Wuerzburg for the DCC-Relevant Age Group (2-6 years; orange) and Corresponding Incidences Among Children in all 69 DCCs (blue) During the Study Period

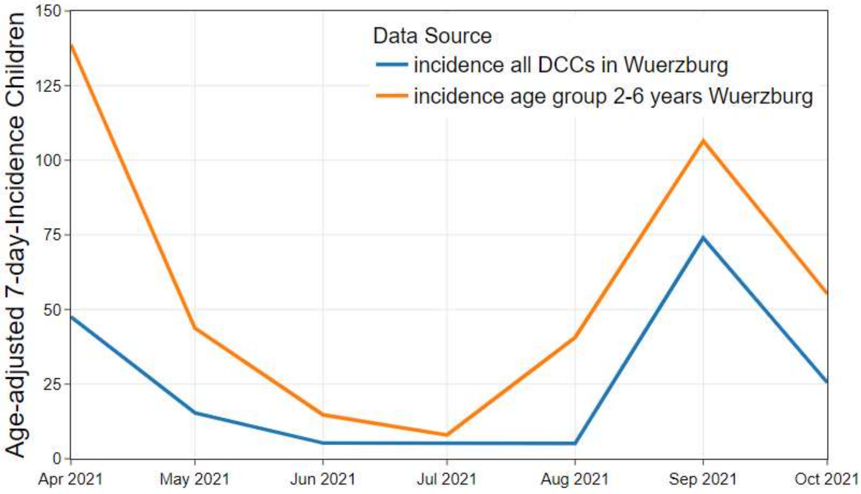

© 2022 Engels G et al. *JAMA Network Open.*

eReferences.

1. Forster J, Streng A, Rudolph P, et al. Feasibility of SARS-CoV-2 Surveillance Testing Among Children and Childcare Workers at German Day Care Centers: A Nonrandomized Controlled Trial. *JAMA Network Open*. 2022;5(1):e2142057-e2142057. doi:10.1001/jamanetworkopen.2021.42057
2. Krone M, Gutling J, Wagener J, et al. Performance of Three SARS-CoV-2 Immunoassays, Three Rapid Lateral Flow Tests, and a Novel Bead-Based Affinity Surrogate Test for the Detection of SARS-CoV-2 Antibodies in Human Serum. *J Clin Microbiol*. Jul 19 2021;59(8):e0031921. doi:10.1128/JCM.00319-21
3. Bethell CD, Read D, Stein RE, Blumberg SJ, Wells N, Newacheck PW. Identifying children with special health care needs: development and evaluation of a short screening instrument. *Ambul Pediatr*. Jan-Feb 2002;2(1):38-48. doi:10.1367/1539-4409(2002)002<0038:icwshc>2.0.co;2
4. Achenbach TM. *Achenbach system of empirically based assessment (ASEBA) : development, findings, theory, and applications*. University of Vermont, Research Center of Children, Youth & Families; 2009.
5. Flanders WD, Kleinbaum DG. Basic models for disease occurrence in epidemiology. *Int J Epidemiol*. Feb 1995;24(1):1-7. doi:10.1093/ije/24.1.1

© 2022 Engels G et al. *JAMA Network Open.*
