# Supplementary material for: Acceptance of Different Self-sampling Methods for Semiweekly SARS-CoV-2 Testing in Asymptomatic Children and Childcare Workers at German Day Care Centers: A Nonrandomized Controlled Trial
Source: JAMA Netw Open. 2022 Sep 15;5(9):e2231798. doi: 10.1001/jamanetworkopen.2022.31798 (PMC9478779; doi:10.1001/jamanetworkopen.2022.31798)
Supplement: Supplement 3. — Nonauthor Collaborators [file jamanetwopen-e2231798-s003.pdf]

Supplemental Online Content: Nonauthor Collaborators

*Indicates required information. Only first name, last name, and suffix will appear in PubMed.

***Group Name(s): Wue-KiTa-CoV 2.0-Study Group**							
***First Name and Middle Initial(s)**	***Last Name**	***Suffix (eg, Jr, III)**	Academic Degrees	Institution	Location (city, state/province, country)	Role or Contribution, eg, chair, principal investigator	Group (if more than 1 Group listed in the byline) and/or Subgroup (eg, Steering Committee)
Annsophie	Amman	-		Institute for Hygiene and Microbiology, University of Wuerzburg	Würzburg, Germany	Field activities, data entry	
Anastasia	Besenfelder	-		Institute for Hygiene and Microbiology, University of Wuerzburg	Würzburg, Germany	Technical support	
Leonie	Bode	-		Institute for Hygiene and Microbiology, University of Wuerzburg	Würzburg, Germany	Field activities, data entry	
Inga	Enders	-		Institute for Hygiene and Microbiology, University of Wuerzburg	Würzburg, Germany	Field activities, data entry	
Thiemo	Frank	-		Institute for Hygiene and Microbiology, University of Wuerzburg	Würzburg, Germany	Field activities, data entry	
Kathrin	Frei	-		Institute for Hygiene and Microbiology, University of Wuerzburg	Würzburg, Germany	Technical support	
Greta	Habersack	-		Institute for Hygiene and Microbiology, University of Wuerzburg	Würzburg, Germany	Field activities, data entry	
Viktoria	Hamway	-		Institute for Hygiene and Microbiology, University of Wuerzburg	Würzburg, Germany	Field activities, data entry	
Theresa	Höferth	-		Institute for Hygiene and Microbiology, University of Wuerzburg	Würzburg, Germany	Technical support	

Supplemental Online Content: Nonauthor Collaborators

*Indicates required information. Only first name, last name, and suffix will appear in PubMed.

***First Name and Middle Initial(s)**	***Last Name**	***Suffix (eg, Jr, III)**	Academic Degrees	Institution	Location (city, state/province, country)	Role or Contribution, eg, chair, principal investigator	Group (if more than 1 Group listed in the byline) and/or Subgroup (eg, Steering Committee)
Thiên-Tri	Lâm	-	MD	Institute for Hygiene and Microbiology, University of Wuerzburg	Würzburg, Germany	Advice on coordination	
Sabrina	Mündlein	-		Institute for Hygiene and Microbiology, University of Wuerzburg	Würzburg, Germany	Technical support	
Konstanze	Rauch	-		Institute for Hygiene and Microbiology, University of Wuerzburg	Würzburg, Germany	Technical support	
Christoph	Schoen		MD, PhD, MSc	Institute for Hygiene and Microbiology, University of Wuerzburg	Würzburg, Germany	Advice on coordination	
Timo	Spannagel	-		Institute for Hygiene and Microbiology, University of Wuerzburg	Würzburg, Germany	Field activities, data entry	
Fabian	Rothbauer	-		Institute for Hygiene and Microbiology, University of Wuerzburg	Würzburg, Germany	Field activities, data entry	
Marianna	Abert	-		Department of Pediatrics, University Hospital Wuerzburg	Würzburg, Germany	Field activities	
Julia	Bley	-	MD	Department of Pediatrics, University Hospital Wuerzburg	Würzburg, Germany	Field activities	
Tatjana	Durnev	-		Department of Pediatrics, University Hospital Wuerzburg	Würzburg, Germany	Communication with study participants	
Carolin	Glatzle	-		Department of Pediatrics, University Hospital Wuerzburg	Würzburg, Germany	Field activities	
Antonia	Hardung	-		Department of Pediatrics, University Hospital Wuerzburg	Würzburg, Germany	Field activities	
Katharina	Karch	-		Department of Pediatrics, University Hospital Wuerzburg	Würzburg, Germany	Field activities	

Supplemental Online Content: Nonauthor Collaborators

*Indicates required information. Only first name, last name, and suffix will appear in PubMed.

*First Name and Middle Initial(s)	*Last Name	*Suffix (eg, Jr, III)	Academic Degrees	Institution	Location (city, state/province, country)	Role or Contribution, eg, chair, principal investigator	Group (if more than 1 Group listed in the byline) and/or Subgroup (eg, Steering Committee)
Sebastian	Kollert	-		Department of Pediatrics, University Hospital Wuerzburg	Würzburg, Germany	Field activities, data entry	
Tanja	Mastorakis	-		Department of Pediatrics, University Hospital Wuerzburg	Würzburg, Germany	Field activities	
Lena	Schregelmann	-		Department of Pediatrics, University Hospital Wuerzburg	Würzburg, Germany	Field activities	
Victoria	Sokalski	-		Department of Pediatrics, University Hospital Wuerzburg	Würzburg, Germany	Field activities	
Eva-Lotta	Tanzberger	-		Department of Pediatrics, University Hospital Wuerzburg	Würzburg, Germany	Data entry	
Anne	Thieme	-	MD	Department of Pediatrics, University Hospital Wuerzburg	Würzburg, Germany	Field activities	
Denise	Yilmaz	-		Department of Pediatrics, University Hospital Wuerzburg	Würzburg, Germany	Field activities, data entry	
Stefan	Zimmerling	-		Department of Pediatrics, University Hospital Wuerzburg	Würzburg, Germany	Field activities	
Pauline	Nehm	-		Hematological Laboratory, University Hospital Wuerzburg	Würzburg, Germany	Field activities	
Max	Siegl	-		Hematological Laboratory, University Hospital Wuerzburg	Würzburg, Germany	Field activities	
Brigitte	Wehner	-		Hematological Laboratory, University Hospital Wuerzburg	Würzburg, Germany	Field activities	
Kerstin	Knies	-	PhD	Institute for Virology and Immunobiology, University of Wuerzburg	Würzburg, Germany	Technical support	
Rebecca	Richter	-		Institute for Virology and Immunobiology, University of Wuerzburg	Würzburg, Germany	Technical support	
Hülya	Düber	-	PhD	City of Wuerzburg	Würzburg, Germany	Advice on coordination	

Supplemental Online Content: Nonauthor Collaborators

*Indicates required information. Only first name, last name, and suffix will appear in PubMed.

***First Name and Middle Initial(s)**	***Last Name**	***Suffix (eg, Jr, III)**	Academic Degrees	Institution	Location (city, state/province, country)	Role or Contribution, eg, chair, principal investigator	Group (if more than 1 Group listed in the byline) and/or Subgroup (eg, Steering Committee)
Monika	Kraft	-		City of Wuerzburg	Würzburg, Germany	Advice on coordination	
Christian	Schuchardt	-		City of Wuerzburg	Würzburg, Germany	Advice on coordination	
Barbara	Finkenberg	-	MD	Health Department City and District Wuerzburg	Würzburg, Germany	Advice on coordination	
Johann	Löw	-	MD	Health Department City and District Wuerzburg	Würzburg, Germany	Advice on coordination	
