# Supplementary material for: Acceptance of Different Self-sampling Methods for Semiweekly SARS-CoV-2 Testing in Asymptomatic Children and Childcare Workers at German Day Care Centers: A Nonrandomized Controlled Trial
Source: JAMA Netw Open. 2022 Sep 15;5(9):e2231798. doi: 10.1001/jamanetworkopen.2022.31798 (PMC9478779; doi:10.1001/jamanetworkopen.2022.31798)
Supplement: Supplement 4. — Data Sharing Statement [file jamanetwopen-e2231798-s004.pdf]

Data Sharing Statement

Engels. Acceptance of Different Self-sampling Methods for Semiweekly SARS-CoV-2 Testing in Asymptomatic Children and Childcare Workers at German Day Care Centers. *JAMA Netw Open*. Published September 15, 2022. doi:10.1001/jamanetworkopen.2022.31798

Data
Data available: Yes
Data types: Participant data with identifiers
How to access data: liese_j@ukw.de (corresponding author)
When available: With publication

Supporting Documents
Document types: None

Additional Information
Who can access the data: Researchers whose proposed use of the data has been approved
Types of analyses: Research in the context infectious diseases spreading in child day care
Mechanisms of data availability: After approval of a proposal by the corresponding author
